# Some mechanistic aspects regarding the Suzuki–Miyaura reaction between selected *ortho*-substituted phenylboronic acids and 3,4,5-tribromo-2,6-dimethylpyridine

**DOI:** 10.3762/bjoc.14.214

**Published:** 2018-09-11

**Authors:** Piotr Pomarański, Piotr Roszkowski, Jan K Maurin, Armand Budzianowski, Zbigniew Czarnocki

**Affiliations:** 1 Faculty of Chemistry, University of Warsaw, Pasteura 1, 02-093 Warsaw, Poland; 2 National Medicines Institute, Chełmska 30/34, 00-725 Warsaw, Poland; 3 National Centre for Nuclear Research, 05-400 Otwock-Świerk, Poland

**Keywords:** arylpyridines, atropisomerism, cross-coupling, palladium, Suzuki–Miyaura reaction

## Abstract

**Background:** Atropisomers are very interesting stereoisomers having axial chirality resulting from restricted rotation around single bonds and are found in various classes of compounds. *ortho-*Substituted arylpyridines are an important group of them. A regio- and atropselective Suzuki–Miyaura cross-coupling reaction on 3,4,5-tribromo-2,6-dimethylpyridine was studied.

**Results:** Reactions with various amounts of *ortho*-substituted phenylboronic acids with 3,4,5-tribromo-2,6-dimethylpyridine gave a series of mono- di- and triarylpyridine derivatives which allowed to draw conclusions about the order of substitution. Also, the observed selectivity in the case of *ortho*-methoxyphenylboronic acid suggested an additional metal *O*-chelation effect in the transition state, apparently not present in the *ortho*-chloro analogues. The rotational barrier in selected atropisomers was determined on the basis of HT NMR and thermal epimerisation experiments. The structure of most presented atropisomeric derivatives of 2,6-dimethylpyridine was confirmed by single-crystal X-ray analysis. Racemic chiral, differently substituted atropisomers were also examined by ^1^H NMR spectroscopy in the presence of a chiral solvating agent.

**Conclusion:** This regio- and atropselectivity may be generally applicable to other arylpyridine systems. A regio- and atropselective Suzuki–Miyaura cross-coupling process has been observed, giving an efficient access to a class of atropisomeric compounds. An opposite selectivity using a differently *ortho*-substituted phenylbornic acid was observed.

## Introduction

Axially chiral biaryls not only subsist in many classes of natural and bioactive compounds [1–2] but also are an essential stereochemical element of many popular, commercially available chiral catalysts [3]. Several *ortho*-substituted arylpyridine derivatives belong to a very important class of axially chiral compounds which have gained interest due to their role as chiral ligands in cross-coupling reactions, or being interesting molecules with important electrochemical, photochemical, including light-activated unidirectional motion properties [4–9]. It is therefore not surprising that important advances have been made in the synthesis of various classes of axially chiral organic compounds over the past decade [10–13].

In recent years, many successful attempts to regioselective [14–17], chemoselective [18–21] or atropselective [1,22–26] synthesis of biaryls were presented, often taking advantage of the popular and useful Suzuki–Miyaura cross-coupling reaction. Genazzani and co-workers described a rapid strategy for the synthesis of potent combretastatin analogues based on the two-step regioselective Suzuki cross-coupling [14]. Beaudry and co-workers used non-symmetric dibromobenzenes in the regioselective Suzuki reactions with phenyl- and selected *para*-substituted boronic acids to obtain the desired coupling products with good selectivity and yield [15]. Recently, the synthesis of some differently *para*- and *meta*-substituted derivatives of 2,6-diaryl-3-(trifluoromethyl)pyridine by regioselective Suzuki–Miyaura reactions was also described [16]. In this case chloropyridines, significantly less-reactive in palladium cross-coupling reaction, were used as substrates allowing the formation of the desired products. Another regioselective Suzuki–Miyaura cross-coupling reaction on 3’,5’-dibromopyridinium *N*-(2’-azinyl)aminides afforded a series of 3-aryl(or heteroaryl)-5’-bromopyridinium *N*-(2’-pyrazinyl)aminides [17]. In 2014, a regiocontrolled polyarylation of pyridine was presented by Doebelin and co-workers [27].

Rotationally restricted biaryls are stereochemically and structurally important elements of a rapidly growing class of catalysts, natural products and chiral auxiliaries. Therefore, the atropselective synthesis is an important synthetic approach pursued by many research groups [22–26]. In 2015 the first phosphoric acid-catalysed asymmetric reactions of 2-naphthols with quinone analogues were described, allowing an access to a class of sterically demanding chiral biaryldiols with excellent enantioselectivities [23]. Recently, highly atropselective synthesis of arylpyrroles by the catalytic asymmetric Paal–Knorr reaction for the synthesis of enantiomerically pure arylpyrroles was presented by Tan [25]. Also as an alternative to palladium couplings, Tanaka presented an atropselective synthesis of axially chiral all-benzenoid biaryls by gold-catalysed intramolecular hydroarylation of alkynones to give the desired atropisomeric product with a good ee value of 70% [26].

Conventional approaches to the synthesis of biaryl compounds having axial chirality entails a direct, atropselective aryl–aryl bond formation step under asymmetric induction provided by internal or external factors. Also, the optical activation in the synthesis of biaryls may involve the separation of diastereomeric derivatives, or more elegantly, may be done by enantioselective transformation [22]. In such cases the stereoselectivity may depend on additional chelation effects [28–34]. Buchwald and co-workers reported an efficient stereoselective synthesis of axially chiral biarylamides by Pd–O bond formation during the oxidative addition step [28–29]. Also other research groups have shown beneficial impact of the additional palladium chelation on the products distribution [31–34].

We also observed an additional chelation effect (*N*-chelation) in the case of 4-amino-3,5-diaryl-2,6-dimethylpyridine derivatives [30].

The above mentioned examples illustrate the importance of the factors governing the selectivity in the Suzuki–Miyaura reactions and encouraged us to study the factors that are important for the mechanism of the formation of selected 3,4,5-triarylated pyridines.

## Results and Discussion

**Figure 1:**
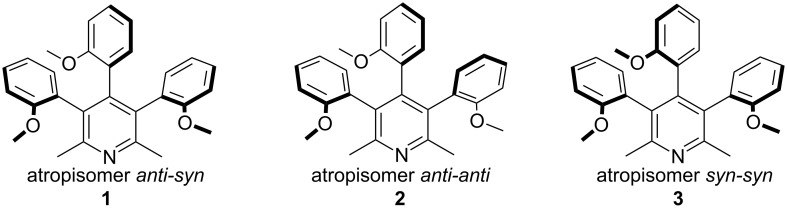
Structures of stereoisomers of 3,4,5-tris(2-methoxyphenyl)-2,6-dimethylpyridines determined by X-ray analysis [38].

In the last few years we have been interested in the phenomenon of atropisomerism occurring in *ortho*-substituted di-, tri- and pentaarylpyridine derivatives [35–38]. The presence of this phenomenon was found in several compounds and also in byproducts formed during the synthesis of analogs of amphetamine prepared by the Leuckart method. The treatment of 3,4,5-tribromo-2,6-dimethylpyridine with 2-methoxyphenylboronic acid under Suzuki–Miyaura reaction conditions gave the mixture of three atropisomeric stereoisomers of 3,4,5-tri-(2-methoxyphenyl)-2,6-dimethylpyridines which were separated by column chromatography and characterized by NMR spectroscopy and X-ray crystallography (Figure 1) [38]. Surprisingly, the least thermodynamically stable atropisomer *syn*-*syn-***3** was isolated as a main product and the proportion of isomers **1**:**2**:**3** was ca. 8:42:50.

This observation stimulated us to further investigate the mechanism of the palladium cross-coupling between **4** with **5** (Table 1). For this purpose we performed a more detailed study of the sequence of coupling with 3,4,5-tribromo-2,6-lutidine. Therefore, a series of Suzuki–Miyaura cross-coupling reactions under different reaction conditions were performed with an amount of boronic acid being systematically reduced. The cross-coupling reaction of 3,4,5-tribromo-2,6-dimethylpyridines (**4**) with limited amount of *ortho*-methoxyphenylboronic acid (**5**, from 1 to 3 equivalents) gave a mixture of atropisomeric, differently mono- and disubstituted 2,6-lutidine derivatives. Fortunately, the mixture of these derivatives proved to be relatively easy to separate due to sufficiently different retention factors on TLC allowing their effective separation by column chromatography. All compounds exhibited characteristic signals in ^1^H NMR which facilitates their identification in the mixtures. The structure of products **6**–**9** were unambiguously determined by the single-crystal X-ray methods (see Supporting Information File 1 for details).

**Table 1:** Optimization for the synthesis **6**–**9**. Compound **10** was not detected in the mixture.

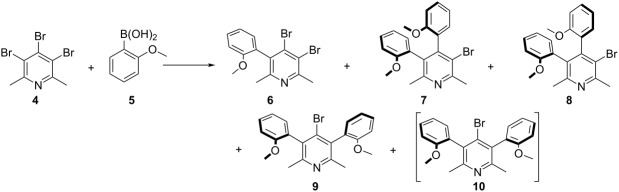										

Entry	**5** (equiv)	Solvent	Catalyst^a^	Base (equiv)	Temp. (°C)	Time (min)	Yield (%)^b^			

							**6**	**7**	**8**	**9**

1	1	toluene	Pd(OAc)_2_, SPhos	K_3_PO_4_ (3)	50	60	26	28	13	15
2	1	toluene	Pd(OAc)_2_, SPhos	K_3_PO_4_ (3)	50	120	22	29	13	17
3	1	toluene	Pd(OAc)_2_, SPhos	K_3_PO_4_ (3)	70	60	30	24	18	16
4	1	toluene	Pd(OAc)_2_, SPhos	K_3_PO_4_ (3)	90	60	23	27	18	15
5	1	toluene	Pd(OAc)_2_, SPhos	K_3_PO_4_ (3)	110	60	27	31	21	16
6	1	toluene	Pd(OAc)_2_, SPhos	K_3_PO_4_ (3)	70	10	24	35	15	21
7	1	toluene	Pd(OAc)_2_, SPhos	K_3_PO_4_ (3)	90	10	26	32	17	15
8	1	toluene	Pd(OAc)_2_, SPhos	K_3_PO_4_ (3)	90	2	17	20	8	11
9	1	toluene	Pd(OAc)_2_, SPhos	Na_2_CO_3_ (3)	90	60	22	29	15	14
10	1	toluene	Pd(OAc)_2_^c^	K_3_PO_4_ (3)	90	60	–	–	–	–
11	1	toluene	PdCl_2_[CH_3_CN]_2_^c^	K_3_PO_4_ (3)	90	60	–	–	–	–
12^d^	2	toluene	Pd(OAc)_2_, SPhos	K_3_PO_4_ (3)	90	60	16	28	19	15
13^e^	3	toluene	Pd(OAc)_2_, SPhos	K_3_PO_4_ (3)	90	60	13	30	18	11

^a^5.0 mol % for both components. ^b^Isolated yield. ^c^10 mol %. ^d^Additionally formation of 9% of **1**–**3**. ^e^Additionally formation of 16% of **1**–**3**.

The presented data revealed that the 1,2 substitution is preferred over the 1,3 substitution which is consistent with the NMR-based method of Handy and Zhang [39]. The most abundant product of coupling in the group of disubstituted compounds is (*syn*)-**7** (Table 1, entries 1–13) although it is thermodynamically unstable (kinetic product). Apparently, the selectivity in the case of the use of *ortho*-methoxyphenylboronic acid is also governed by an additional metal *O*-chelation effect in the transition state what causes that isomer (*anti*)-**8** is formed in a smaller amount. The oxygen atom may serve as an extra ligand. Therefore, the coordination by the methoxy group to palladium may cause changes in the geometry of the complex, reflected in the atropisomers distribution. A similar beneficial chelation effect was observed by Buchwald [28–29]. The origin of the enantioselectivity during the selectivity-determining step of the coupling of tolylboronic acid with naphthylphosphonate bromide was proposed by additional *O*-chelation. Previously, we also observed additional *N*-chelation during the synthesis of 4-amino-3,5-diaryl-2,6-dimethylpyridine derivatives [30].

The use of different cross-coupling conditions did not significantly change the product distribution of the coupling reaction (Table 1). Interestingly, only one mono-substituted derivative **6** was observed in the reaction mixture.

The atropisomer (*syn*)-**10** of compound (*anti*)-**9** was not detected in the reaction mixture but was obtained through atropisomerisation of (*anti*)-**9** in xylene at 140 °C by prolonged heating (8 h). Compounds **9** and **10** have very similar chromatographic properties and their separation was possible by column chromatography using a specialist high-purity grade silica gel type *H* (10–40 µm). The structure of (*syn*)-**10** was also confirmed by the single-crystal X-ray analysis (see Supporting Information File 1 for details).

We also carried out a series of the cross-coupling reactions using 3,4-dibromo-5-(2-methoxyphenyl)-2,6-dimethylpyridine (**6**) as a substrate in comparison to 3,4,5-tribromo-2,6-dimethylpyridine as substrate. The variation of temperature, reaction time and amount of boronic acid caused only minute changes in the distribution of atropisomers **1**–**3** with the same almost quantitative total yield (Table 2).

**Table 2:** Optimization of the synthesis of compounds **1**–**3**.

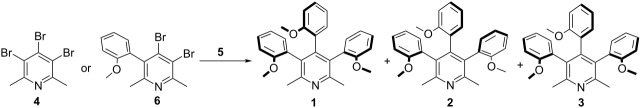										

Entry	Substrate	**5** (equiv)	Solvent	Catalyst^a^	Base (equiv)	Temp. (°C)	Time (min)	Yield (%)^b^		

								**1**	**2**	**3**

1	**4**	9	toluene	Pd(OAc)_2_, SPhos	K_3_PO_4_ (9)	90	60	47	40	10
2	**4**	9	toluene	Pd(OAc)_2_, SPhos	K_3_PO_4_ (9)	70	60	52	37	8
3	**4**	9	xylene	Pd(OAc)_2_, SPhos	K_3_PO_4_ (9)	120	60	40	43	17
4	**4**	12	toluene	Pd(OAc)_2_, SPhos	K_3_PO_4_ (9)	90	10	50	45	5
5	**4**	9	toluene	Pd_2_(PPh_3_)_4_, SPhos	K_3_PO_4_ (9)	90	10	52	44	4
6	**6**	9	toluene	Pd(OAc)_2_, SPhos	K_3_PO_4_ (3)	90	60	56	32	5
7	**6**	12	toluene	Pd(OAc)_2_, SPhos	K_3_PO_4_ (3)	90	60	55	29	3
8	**6**	9	xylene	Pd(OAc)_2_, SPhos	K_3_PO_4_ (3)	120	60	44	43	10
9	**6**	9	toluene	Pd(OAc)_2_, SPhos	K_3_PO_4_ (3)	60	60	50	41	5

^a^5.0 mol % for both components. ^b^Isolated yield.

**Figure 2:**
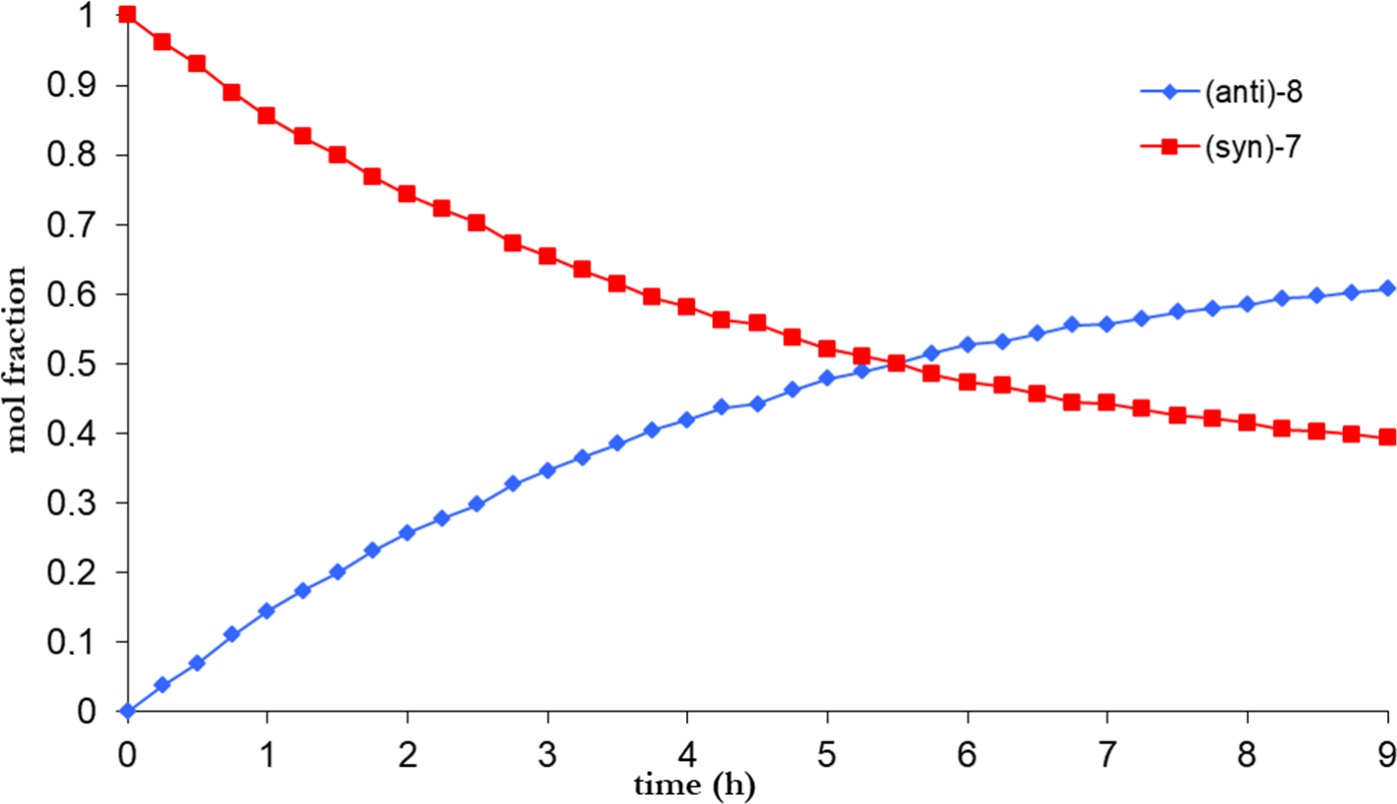
Graphical representation of kinetic, time-dependent ^1^H NMR analysis of (*syn*)-**7** (100 °C).

**Figure 3:**
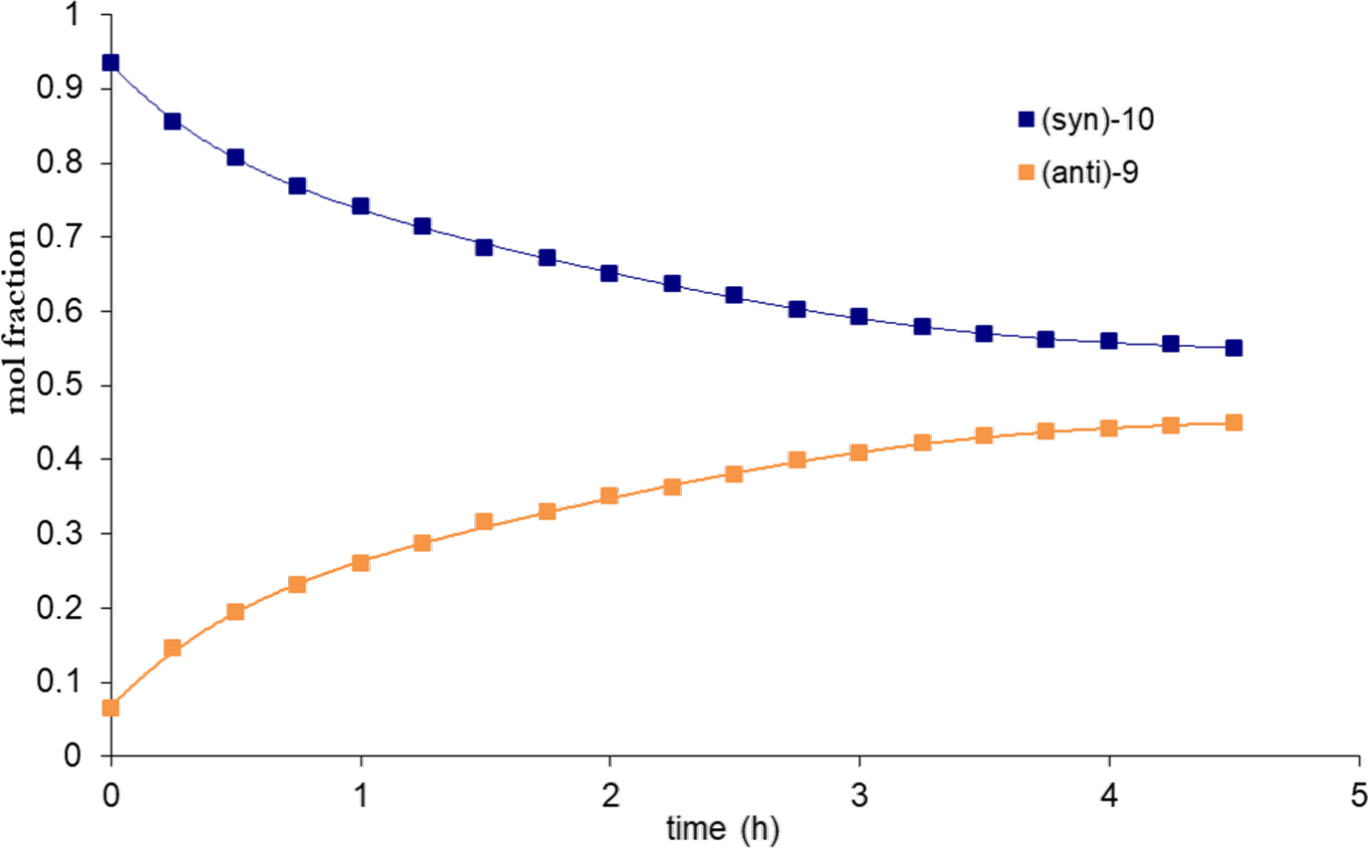
Graphical representation of kinetic, time-dependent ^1^H NMR analysis of (*syn*)-**10** (120 °C).

**Figure 4:**
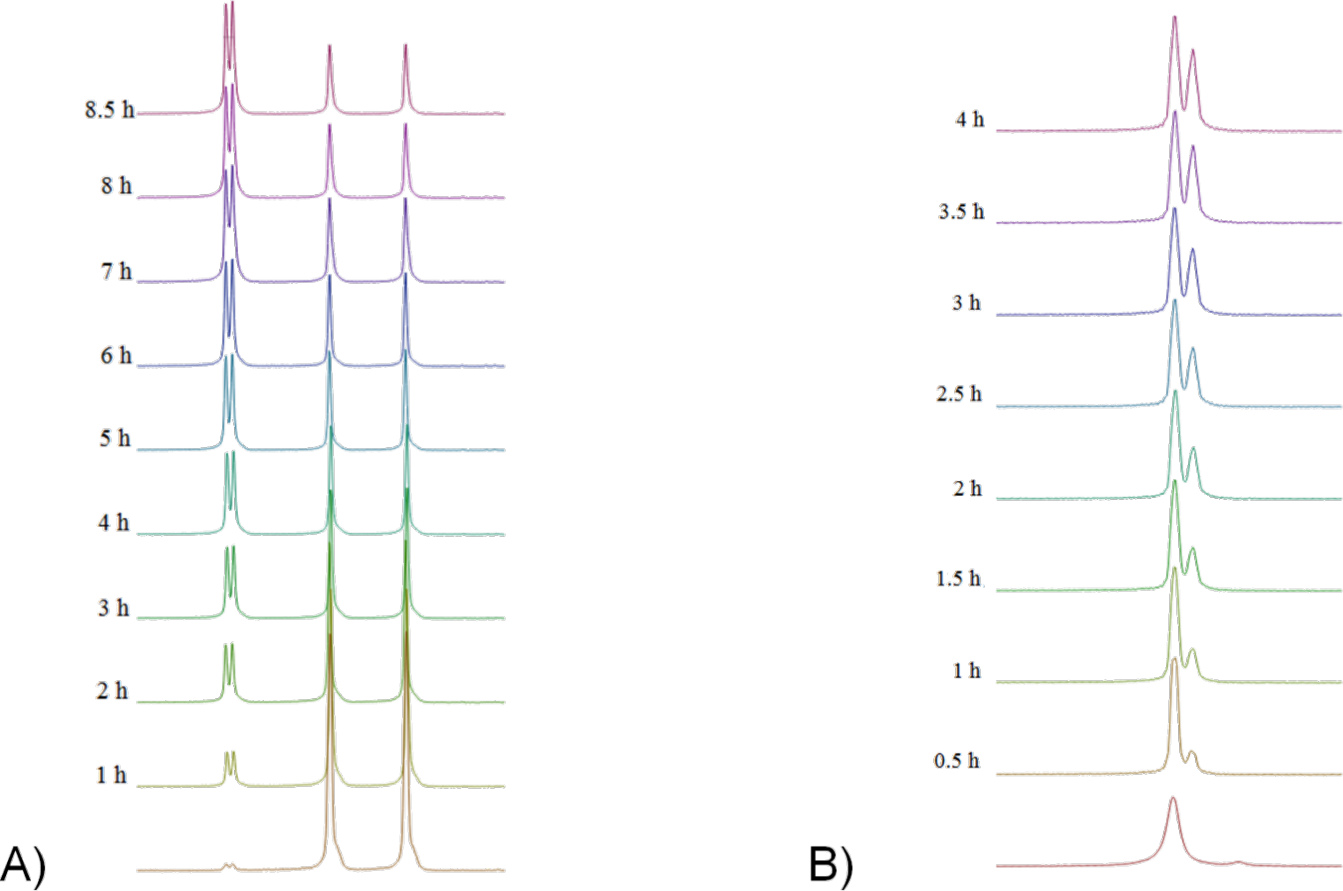
HT-NMR (300 MHz, DMSO-*d*_6_) spectra of A) (*syn*)-**7**. B) (*syn*)-**10**. Only the upfield (ca. 3.4–4 ppm) region of the spectrum is shown. In the figure are shown quantitative changes for signals from the methoxy group. Full spectra for individual atropisomers are shown in Supporting Information File 1.

Considering further applications of atropisomeric arylated pyridines in the design of new materials like molecular switches [4], it would be desirable to estimate their thermal stability. We therefore performed kinetic experiments in order to establish the value of the barrier to rotation in the atropisomerisation process by using a dynamic ^1^H NMR spectroscopy carried out on more unstable *syn* atropisomers, which are (*syn*)-**7** and (*syn*)-**10**. The experiments were performed at given temperature in deutered DMSO while observing the time dependence of the intensity of signal methoxy groups (Figures 2–4). For both isomers (*syn*)-**7** and (*anti*)-**8** we analyzed signals having chemical shifts of 3.50 ppm, 3.57 ppm and 3.64 ppm, respectively. In the case of derivative (*syn*)-**10** and (*anti*)-**9** the observed signals of the chemical shifts are at 3.76 and 3.74 ppm.

We are able to calculate the value of the equilibrium constant and then the activation energy of rotation (ΔG^#^) using the Eyring equation [40]. Therefore, we estimated the respective energy barriers to be as Δ*G*^#^**_7_**_-_**_8_** = 21.7 kcal/mol and Δ*G*^#^**_9_**_-_**_10_** = 23.4 kcal/mol (Table 3).

**Table 3:** Rotational barriers of compounds **7** and **10**.

Entry	Compound	[*syn*]_equiv_	[*anti*]_equiv_	*K*	*T* (°C)	Δ*G*^# a,b^ (kcal/mol)

1	**7**	0.39	0.61	1.54	100	21.7
2	**10**	0.55	0.45	0.81	120	23.4

a) Estimated margin of error ±0.19 kcal/mol. b) Δ*G*^#^ = *RT*[23.76 − ln(*K*/*T*)]

In order to verify our observations that may result from additional *O*-chelation of palladium we used a boronic acid which cannot coordinate the palladium atom and having an electron withdrawing group (for example *ortho*-chlorophenylboronic acid). We therefore performed a series of reactions of brominated 2,6-lutidine **4** with 9–12 equiv of *ortho*-chlorophenylboronic acid **11** in different coupling conditions to obtain three atropisomeric derivatives **12**–**14** in good total yield (Table 4). Compound **14** was easily separated from the reaction mixture by column chromatography (hexane/ethyl acetate as eluent). However, the separation of individual atropisomers **12** and **13** having almost the same *R*_f_ value was only possible by careful crystallization from methanol. The structure and configuration of the products **12**–**14** were established by NMR as well as the single-crystals X-ray analysis (see Supporting Information File 1 for details).

**Table 4:** Optimization of the reaction conditions for the synthesis of **12**–**14**.

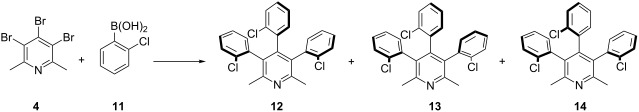									

Entry	**11** (equiv)	Solvent	Catalyst^a^	Base (equiv)	Temp. (°C)	Time (min)	Yield (%)^b^		

							**12**	**13**	**14**

1	9	toluene	Pd(OAc)_2_, SPhos	K_3_PO_4_ (9)	90	30	42	35	11
2	9	toluene	Pd(OAc)_2_, SPhos	K_3_PO_4_ (9)	70	30	40	33	12
3	9	toluene	Pd(OAc)_2_, SPhos	K_3_PO_4_ (9)	50	60	27	21	7
4	12	toluene	Pd(OAc)_2_, SPhos	K_3_PO_4_ (9)	90	10	45	39	13
5	12	toluene	Pd(OAc)_2_, SPhos	K_3_PO_4_ (9)	70	15	44	34	10
6	12	toluene	Pd(OAc)_2_, SPhos	K_3_PO_4_ (9)	50	30	25	20	6
7	12	xylene	Pd(OAc)_2_, SPhos	K_3_PO_4_ (9)	120	15	45	37	15

^a^5.0 mol % for both components. ^b^Isolated yield.

The data presented in Table 4 clearly indicate that the substituent change from methoxy to chlorine result in different atropisomers propagation. In the absence of additional *O*-chelation, the isomer with the lowest energy (*anti,syn*)-**12** is formed as the main product. According to theoretical predictions the thermodynamically unfavorable atropisomers (*syn,syn*)-**14** is formed with low yield. In order to get better insight into the order of introduction of aryl rings to the pyridine core, we performed analogous experiments as in the case of *ortho*-methoxy series, with a reduced amount of boronic acid **11** (Table 5). As result, only compounds **15**–**17** were detected in the reaction mixture. They were successfully isolated by column chromatography and their structure was established by spectroscopic and the single-crystal X-ray methods (see Supporting Information File 1 for details).

**Table 5:** Optimization of the reaction conditions for the synthesis of **15**–**17**.

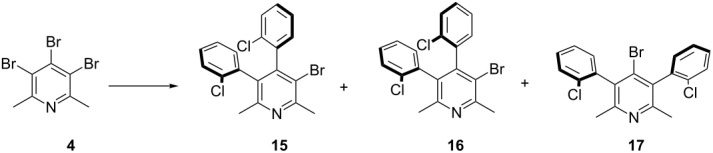									

Entry	**11** (equiv)	Solvent	Catalyst^a^	Base (equiv)	Temp. (°C)	Time (min)	Yield (%)^b^		

							**15**	**16**	**17**

1	4	toluene	Pd(OAc)_2_, SPhos	K_3_PO_4_ (3)	50	60	36	10	14
2	4	toluene	Pd(OAc)_2_, SPhos	K_3_PO_4_ (3)	50	120	40	13	18
3	4	toluene	Pd(OAc)_2_, SPhos	K_3_PO_4_ (3)	70	60	42	9	12
4	4	toluene	Pd(OAc)_2_, SPhos	K_3_PO_4_ (3)	90	60	45	9	13
5	4	toluene	Pd(OAc)_2_, SPhos	K_3_PO_4_ (3)	110	60	46	10	12
6	4	toluene	Pd(OAc)_2_, SPhos	K_3_PO_4_ (3)	70	10	48	7	10
7	4	toluene	Pd(OAc)_2_, SPhos	KF (3)	90	10	44	10	15
8	4	toluene	Pd(OAc)_2_, SPhos	Cs_2_CO_3_ (3)	90	60	34	6	9
9	4	toluene	Pd(OAc)_2_, SPhos	Na_2_CO_3_ (3)	90	60	46	10	16

^a^5.0 mol % for both components. ^b^Isolated yield.

Again, the 1,2 substitution was preferred over the 1,3 substitution (the same as for the coupling with *ortho*-methoxyphenylboronic acid). The most abundant product of coupling was compound (*anti*)-**15** which is thermodynamically the most stable. Different cross-coupling conditions did not affect the products profile (Table 5).

The obtained atropisomeric differently substituted derivatives of *ortho*-chlorophenyl-2,6-dimethylpyridines **12**–**17** turned out to be conformationally stable at room temperature as well as at higher temperatures (at 120 °C for 1 h in DMSO-*d*_6_ by NMR, also at 160 °C in diglyme by TLC) which indicated that the Δ*G*^#^ value for atropisomerization process is above 30 kcal/mol [40].

**Figure 5:**
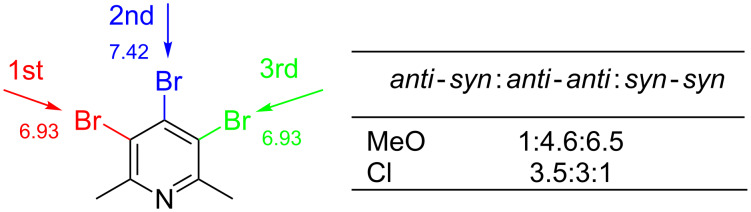
Summary of the results for coupling with *ortho*-substituted phenylboronic acid for triaryl products.

**Figure 6:**
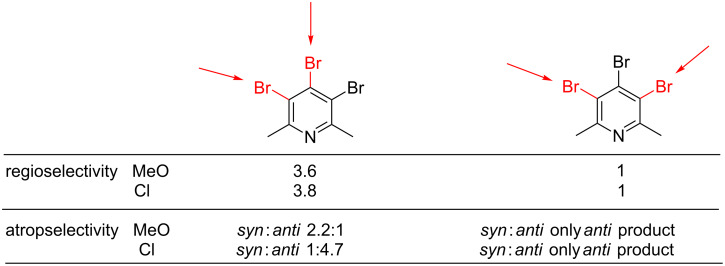
Summary of results for coupling with *ortho*-substituted phenylboronic acid for diaryl products.

It is already known that the palladium catalysed cross-coupling reaction usually occurs at the electronically more deficient and sterically less hindered position [39,41]. Positions C-3 (or C-5) and C-4 of pyridine **4** are not sterically and electronically similar. The coupling at C-4-position may be preferred due to electronic reasons, but is strongly disfavoured by steric repulsion (bigger van der Waals radius of the bromine atom compared to the methyl group). This may be the reason for the first arylation to occur at the C-3(5) position in compound **4** and the second one preferably at the C-4 position, both in *ortho*-methoxyphenyl and *ortho*-chlorophenyl series (Figure 5 and Figure 6). In the former one, however, the successive arylation is additionally affected by apparent *O*-chelation of the metal at the transition state which results in noticeable diastereoselectivity of this process, leading to a less thermodynamically stable isomer, e.g., (*syn*)-**7**. In the reaction with *ortho*-chlorophenylboronic acid, however, the metal chelation is not present and consequently, the thermodynamic product *anti*-**15** is preferentially formed. This observation may be useful in the design of the stereoselective synthesis of polyarylated systems.

All obtained *ortho*-methoxy-substituted derivatives of pyridine **2** and **6–9** as well as *ortho*-chloro-substituted pyridine derivatives **13**, **15**–**17** are chiral molecules and therefore a method for enantiomer discrimination was needed, especially in the case of the planned asymmetric synthesis of various substituted arylpyridine derivatives. Unfortunately, separation using chiral HPLC columns were unsuccessful and therefore, the racemic mixtures of these atropisomers were examined by ^1^H NMR spectroscopy with a chiral solvating agent ((+)-(*R*)-*tert*-butyl(phenyl)phosphinothioic acid) in order to visualize the presence of individual enantiomers (see Supporting Information File 1 for details).

**Figure 7:**
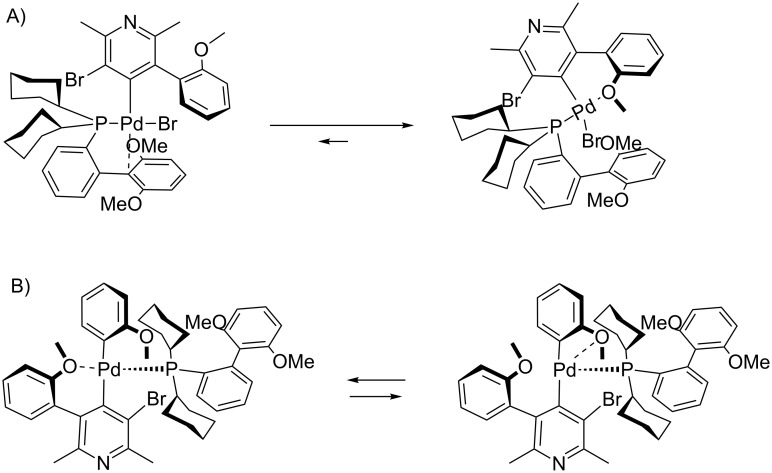
Proposed intermediates for the 1,2-addition of **5** with methoxy group. A) Oxidative addition step. B) Transmetalation step.

**Figure 8:**
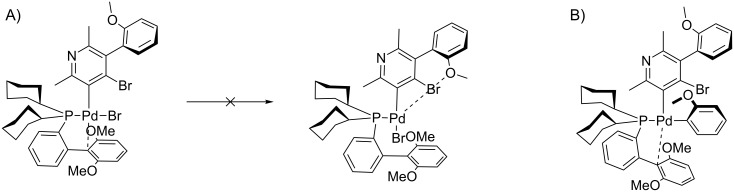
Proposed intermediates for the 1,3-addition with methoxy group. A) Oxidative addition step. B) Transmetalation step.

**Figure 9:**
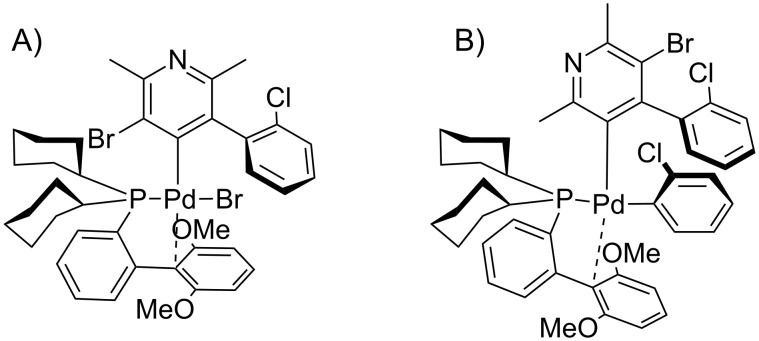
Proposed intermediates for the 1,2-addition with chlorine atom. A) Oxidative addition step. B) Transmetalation step.

**Figure 10:**
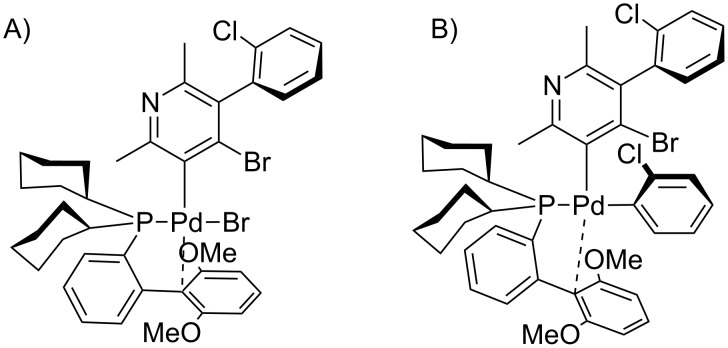
Proposed intermediates for the 1,3-addition with chlorine atom. A) Oxidative addition step. B) Transmetalation step.

Based on the current knowledge on the chemistry of palladium complexes with SPhos [42–44] we proposed a sequence in the arylation process and the substitution mechanism for the reaction of compounds **4** with **5** or **11** which rationalizes obtained results on the basis of *O*-chelation effects (shown in Figures 7–10).

## Conclusion

In conclusion, a regio- and atroposelective Suzuki–Miyaura cross-coupling process has been observed, giving an efficient access to a class of atropoisomeric compounds. The opposite selectivity using differently *ortho*-substituted phenylboronic acids was observed. Both electron-poor and electron-rich arylboronic acids were successfully employed. These results may be helpful in the construction of chiral atropisomeric derivatives of arylpyridine.

## Supporting Information

File 1 Copies of ^1^H and ^13^C NMR spectra; copies of ^1^H and ^13^C NMR spectra at high temperature and ORTEP diagrams of compounds **6**–**17**.
