# Supplementary material for: Some mechanistic aspects regarding the Suzuki–Miyaura reaction between selected ortho-substituted phenylboronic acids and 3,4,5-tribromo-2,6-dimethylpyridine
Source: Beilstein J Org Chem. 2018 Sep 11;14:2384–93. doi: 10.3762/bjoc.14.214 (PMC6142784; doi:10.3762/bjoc.14.214)
Supplement: File 1 — Copies of 1H and 13C NMR spectra; copies of 1H and 13C NMR spectra at high temperature and ORTEP diagrams of compounds 6–17. [file Beilstein_J_Org_Chem-14-2384-s001.pdf]

Some mechanistic aspects regarding the Suzuki–Miyaura reaction between selected *ortho*-substituted phenylboronic acids and 3,4,5-tribromo-2,6-dimethylpyridine

Piotr Pomarański[1], Piotr Roszkowski[1], Jan K. Maurin[2,3], Armand Budzianowski[3] and Zbigniew Czarnocki*[1]

Address: [1]Faculty of Chemistry, University of Warsaw, Pasteura 1, 02-093 Warsaw, Poland, [2]National Medicines Institute, Chełmska 30/34, 00-725 Warsaw, Poland and [3]National Centre for Nuclear Research, 05-400 Otwock-Świerk, Poland

Email: Zbigniew Czarnocki - czarnoz@chem.uw.edu.pl
*Corresponding author

Copies of ^{1}H and ^{13}C NMR spectra; copies of ^{1}H and ^{13}C NMR spectra at high temperature and ORTEP diagrams of compounds 6–17

Experimental section: S4

^{1}H NMR spectrum of **6** (300 MHz, $CDCl_3$) S8

^{13}C NMR spectrum of **6** (75 MHz, $CDCl_3$) S8

^{1}H NMR spectrum of **7** (300 MHz, $CDCl_3$) S9

^{13}C NMR spectrum of **7** (75 MHz, $CDCl_3$) S9

^{1}H NMR spectrum of **8** (300 MHz, $CDCl_3$) S10

^{13}C NMR spectrum of **8** (75 MHz, $CDCl_3$) S10

^{1}H NMR spectrum of **7** (300 MHz, DMSO)........ S11

^{1}H NMR spectrum of **8** (300 MHz, DMSO)........ S11

^{1}H NMR spectrum of **9** (300 MHz, $CDCl_3$) S12

^{13}C NMR spectrum of **9** (75 MHz, $CDCl_3$) S12

^{1}H NMR spectrum of **10** (300 MHz, $CDCl_3$) S13

^{13}C NMR spectrum of **10** (75 MHz, $CDCl_3$) S13

^{1}H NMR spectrum of **12** (300 MHz, $CDCl_3$) S14

^{13}C NMR spectrum of **12** (75 MHz, $CDCl_3$) S14

^{1}H NMR spectrum of **12** (300 MHz, DMSO) *T* = 25 °C.. S15

^{1}H NMR spectrum of **12** (300 MHz, DMSO) after 2 h heating at 120 °C, ... S15

^{1}H NMR spectrum of **13** (300 MHz, $CDCl_3$) S16

^{13}C NMR spectrum of **13** (75 MHz, $CDCl_3$) S16

^{1}H NMR spectrum of **13** (300 MHz, DMSO) *T* = 25 °C.. S17

^{1}H NMR spectrum of **13** (300 MHz, DMSO) after 2 h heating at 120 °C S17

^{1}H NMR spectrum of **14** (300 MHz, $CDCl_3$) S18

^{13}C NMR spectrum of **14** (75 MHz, $CDCl_3$) S18

^{1}H NMR spectrum of **14** (300 MHz, DMSO) *T* = 25 °C.. S19

^{1}H NMR spectrum of **14** (300 MHz, DMSO) after 2 h heating at 120°C S19

^{1}H NMR spectrum of **15** (300 MHz, $CDCl_3$) S20

^{13}C NMR spectrum of **15** (75 MHz, $CDCl_3$) S20

^{1}H NMR spectrum of **15** (300 MHz, DMSO) *T* = 25 °C.. S21

^{1}H NMR spectrum of **15** (300 MHz, DMSO) after 2 h heating at 120 °C S21

^{1}H NMR spectrum of **16** (300 MHz, $CDCl_3$) S22

^{13}C NMR spectrum of **16** (75 MHz, $CDCl_3$) S22

^{1}H NMR spectrum of **16** (300 MHz, DMSO) *T* = 25 °C.. S23

^{1}H NMR spectrum of **16** (300 MHz, DMSO) after 2 h heating at 120 °C S23

^{1}H NMR spectrum of **17** (300 MHz, $CDCl_3$) S24

^{13}C NMR spectrum of **17** (75 MHz, $CDCl_3$) S24

^{1}H NMR spectrum of **17** (300 MHz, DMSO) *T* = 25 °C.. S25

^{1}H NMR spectrum of **17** (300 MHz, DMSO) after 2 h heating at 120 °C S25

^{1}H NMR (CH_3 and OCH_3 protons) of racemic **2** in presence of (+)-(*R*)-tert-butyl(phenyl) phosphinothioic acid S26

^{1}H NMR (CH_3 and OCH_3 protons) of racemic **6** in presence of (+)-(*R*)-tert-butyl(phenyl)-phosphinothioic acid S27

^{1}H NMR (CH_3 and OCH_3 protons) of racemic **7** in presence of (+)-(*R*)-tert-butyl(phenyl)-phosphinothioic acid S27

^{1}H NMR (CH_3 and OCH_3 protons) of racemic **8** in presence of (+)-(*R*)-tert-butyl(phenyl)-phosphinothioic acid S28

^{1}H NMR (CH_3 protons) of racemic **9** in presence of (+)-(*R*)-*tert*-butyl(phenyl)phosphinothioic acid .. S28

^{1}H NMR (CH_3 protons) of racemic **13** in presence of (+)-(*R*)-*tert*-butyl(phenyl)phosphinothioic acid S29

^{1}H NMR (CH_3 protons) of racemic **15** in presence of (+)-(*R*)-*tert*-butyl(phenyl)phosphinothioic acid S29

^{1}H NMR (CH_3 protons) of racemic **16** in presence of (+)-(*R*)-*tert*-butyl(phenyl)phosphinothioic acid S30

^{1}H NMR (CH_3 protons) of racemic **17** in presence of (+)-(*R*)-*tert*-butyl(phenyl)phosphinothioic acid S30

ORTEP diagram of compound **6** S31

ORTEP diagram of compound **7** S31

ORTEP diagram of compound **9** S32

ORTEP diagram of compound **10** S32

ORTEP diagram of compound **12** S33

ORTEP diagram of compound **13** S33

ORTEP diagram of compound **14** S34

ORTEP diagram of compound **15** S34

ORTEP diagram of compound **16** S35

ORTEP diagram of compound **17** S35

Experimental section:

All reactions were carried out in oven-dried pressure tube under argon atmosphere in order to simultaneously exclude oxygen and water. Solvents for the reactions were commercially available and were used without further purification. THF or toluene were dried and distilled by standard methods. All solvents were purchased from Sigma Aldrich. TLC analyses were performed on Merck Kieslgel 60 F–254 plates. The visualization of plates was done under UV light or under iodine vapor. All chemicals employed, including ligands, pre-catalyst, boronic acids and bases, were purchased from commercial sources (Sigma Aldrich and Fluorochem) and used without further purification.

Melting points were determined on an Electrothermal Model IA 9200 apparatus and are uncorrected.

NMR spectra were recorded on a Bruker AVANCE 300 MHz spectrometer operating at 300 MHz for ^{1}H NMR and 75 MHz for ^{13}C NMR. The spectra were measured in $CDCl_3$ and are given as δ values (in ppm) relative to TMS. Peak characterization: s = singlet, brs = broad singlet, d = doublet, brd = broad doublet, t = triplet, dd = doublet of doublets, q = quartet, m = multiplet

HRMS spectra were collected on Quattro LC Micromass and LCT Micromass TOF HiRes apparatuses.

X-ray crystallographic data were collected using Xcalibur–R κ-axis single crystal diffractometer from Oxford Diffraction. Data were measured at room temperature using Cu Kα monochromatic radiation. After initial corrections the collected data were used to solve crystal structures and then to refine models. SHELXS–97 and SHELXL–97 software was applied for these purposes. Full structural data have been deposited with Cambridge Structural Data Centre under respective deposit numbers.

General procedure of Suzuki–Miyaura reaction:

A mixture of **4** (500 mg, 1.45 mmol), arylboronic acid (1.0–12.0 equiv), palladium catalyst (5.0 mol %), ligand (5.0 mol %) and base (9.0 equiv) in 10 mL of solvent was stirred at required temperature under argon atmosphere for required time. The reaction was monitored by TLC. After completion of the reaction, the mixture was cooled and quenched with cold water (5 mL). The organic phase was extracted with toluene (3 × 5 mL). Combined organic extracts were dried over anhydrous magnesium sulphate and concentrated under reduced pressure. The crude mixture was purified by flash column chromatography on silica gel (230−400 mesh) using ethyl acetate in hexane to obtain pure compound different-substituted compound in moderate or good yield.

(aR,aS)-3,4-Dibromo-5-(2-methoxyphenyl)-2,6-dimethylpyridine (**6**). White solid, yield 30%, mp 69 °C; R_f 0.57 (ethyl acetate : hexane 1:1); ^{1}H NMR (300 MHz, $CDCl_3$) δ= 2.23 (s, 3H, CH_3), 2.76 (s, 3H, CH_3), 3.76 (s, 3H, OCH_3), 6.98-7.08 (m, 3H, CH), 7.38-7.44 (m, 1H, CH); ^{13}C NMR (75 MHz, $CDCl_3$) δ= 23.7, 27.1, 55.8, 111.3, 121.0, 121.7, 128.5, 130.1, 130.5, 134.3, 138.1, 155.9, 156.4, 157.0; HRMS (ESI+) calcd. for $C_{14}H_{14}Br_2NO$ [M+H]+ 371.9422; found 371.9425. **6** * picrate: yellow solid, mp

168.9-169.8°C, crystallization from CH_2Cl_2/hexane. CCDC-1528450 contains the supplementary crystallographic data for this paper. These data can be obtained free of charge from The Cambridge Crystallographic Data Centre via www.ccdc.cam.ac.uk/data_request/cif.

(syn)-(aR,aS)-3-Bromo-4,5-bis(2-methoxyphenyl)-2,6-dimethylpyridine (**7**). White solid, yield 35%, mp 159 °C; R_f 0.45 (ethyl acetate : hexane 1:1); ^{1}H NMR (300 MHz, $CDCl_3$) δ=2.29 (s, 3H, CH_3), 2.75 (s, 3H, CH_3), 3.59 (s, 3H, OCH_3), 3.62 (s, 3H, OCH_3),6.68-6.89 (m, 6H, CH), 7.10-7.19 (m, 2H, CH); ^{1}H NMR (300 MHz, DMSO) δ= 2.13 (s, 3H, CH_3), 2.64 (s, 3H, CH_3), 3.50 (s, 3H, OCH_3), 3.57 (s, 3H, OCH_3), 6.68-6.92 (m, 6H, CH), 7.09-7.16 (m, 2H, CH); ^{13}C NMR (75 MHz, $CDCl_3$) δ= 23.0, 26.0, 55.0, 55.2, 110.0, 110.3, 119.5, 119.7, 121.0, 126.7, 127.9, 128.7, 129.1, 131.0, 131.6, 132.2, 147.7, 155.0, 155.6, 156.0, 156.5; HRMS (ESI+) calcd. for $C_{21}H_{21}BrNO_2$ [M+H]+ 398.0756; found 398.0739, crystallization from methanol. CCDC-1528451 contains the supplementary crystallographic data for this paper. These data can be obtained free of charge from The Cambridge Crystallographic Data Centre via www.ccdc.cam.ac.uk/data_request/cif.

(anti)-(aR,aS)-3-Bromo-4,5-bis(2-methoxyphenyl)-2,6-dimethylpyridine (**8**). White solid, yield 21%, mp 129.5 °C; R_f 0.5 (ethyl acetate : hexane 1:1); ^{1}H NMR (300 MHz, $CDCl_3$) δ= 2.24 (s, 3H, CH_3), 2.76 (s, 3H, CH_3), 3.67 (s, 3H, OCH_3), 3.69 (s, 3H, OCH_3), 6.68-6.77 (m, 4H, CH), 6.84-6.91 (m, 2H, CH), 7.08-7.17 (m, 2H, CH); ^{1}H NMR (300 MHz, DMSO) δ= 2.10 (s, 3H, CH_3), 2.63 (s, 3H, CH_3), 3.64 (s, 6H, OCH_3), 6.72-6.77 (m, 3H, CH), 6.81-6.86 (m, 3H, CH), 7.11-7.17 (m, 2H, CH); ^{13}C NMR (75 MHz, $CDCl_3$) δ= 23.0, 26.0, 55.2, 55.5, 110.1, 110.3, 119.9, 120.1, 121.0, 127.6, 128.9, 129.1, 129.3, 129.3, 130.2, 132.6, 148.3, 155.1, 155.4, 155.9, 156.3; HRMS (ESI+) calcd. for $C_{21}H_{21}BrNO_2$ [M+H]+ 398.0756; found 398.0739.

(anti)-(aR,aS)-4-Bromo-3,5-bis(2-methoxyphenyl)-2,6-dimethylpyridine (**9**). White solid, yield 21%, mp 187 °C; R_f 0.45 (ethyl acetate : hexane 1:1); ^{1}H NMR (300 MHz, $CDCl_3$) δ= 2.32 (s, 6H, CH_3), 3.78 (s, 6H, OCH_3), 6.98-7.07 (m, 4H, CH), 7.14-7.17 (m, 2H, CH), 7.37-7.43 (m, 2H, CH); ^{13}C NMR (75 MHz, $CDCl_3$) δ= 23.8, 55.6, 111.1, 120.7, 128.5, 129.5, 130.9, 132.2, 156.1, 156.5; HRMS (ESI+) calcd. for $C_{21}H_{21}BrNO_2$ [M+H]+ 398.0756; found 398.0739, crystallization from methanol. CCDC-1528454 contains the supplementary crystallographic data for this paper. These data can be obtained free of charge from The Cambridge Crystallographic Data Centre via www.ccdc.cam.ac.uk/data_request/cif.

(syn)-4-Bromo-3,5-bis(2-methoxyphenyl)-2,6-dimethylpyridine (**10**). White solid, mp 130 °C; R_f 0.45 (ethyl acetate : hexane 1:1); ^{1}H NMR (300 MHz, $CDCl_3$) δ= 2.33 (s, 6H, CH_3), 3.78 (s, 6H, OCH_3), 6.98-7.17 (m, 6H, CH), 7.37-7.43 (m, 2H, CH); ^{13}C NMR (75 MHz, $CDCl_3$) δ= 23.8, 55.7, 111.3, 120.7, 128.5, 129.5, 130.8, 132.3, 156.1, 156.6; HRMS (ESI+) calcd. for $C_{21}H_{21}BrNO_2$ [M+H]+ 398.0756; found

398.0739. **10** * picrate: yellow solid, mp 206.8-207.9°C, crystallization from dichloromethane/hexane. CCDC-1528457 contains the supplementary crystallographic data for this paper. These data can be obtained free of charge from The Cambridge Crystallographic Data Centre via www.ccdc.cam.ac.uk/data_request/cif.

(anti, syn)-3,4,5-Tris(2-chlorophenyl)-2,6-dimethylpyridine (**12**). White solid, yield 45%, mp 204.5 °C; R_f 0.36 (20% ethyl acetate in hexane); ^{1}H NMR (300 MHz, $CDCl_3$) δ= 2.34 (s, 6H, CH_3), 6.84-6.89 (m, 2H, CH), 6.95-6.98 (m, 1H, CH), 7.07-7.14 (m, 4H, CH), 7.22-7.27 (m, 3H, CH), 7.34-7.38 (m, 2H, CH); ^{13}C NMR (75 MHz, $CDCl_3$) δ= 23.2, 125.7, 126.4, 128.1, 128.8, 129.0, 129.0, 130.2, 130.8, 131.9, 133.5, 136.9, 137.1, 155.8; HRMS (ESI+) calcd. for $C_{25}H_{19}Cl_3N$ [M+H]+ 438.0583; found 438.0579. **12** * HCl: white solid, mp 233.5-235°C, crystallization from methanol/ethyl acetate CCDC-1528707 contains the supplementary crystallographic data for this paper. These data can be obtained free of charge from The Cambridge Crystallographic Data Centre via www.ccdc.cam.ac.uk/data_request/cif.

(anti, anti)-(aR,aS)-3,4,5-Tris(2-chlorophenyl)-2,6-dimethylpyridine (**13**). White solid, yield 39%, mp 226 °C; R_f 0.36 (20% ethyl acetate in hexane); ^{1}H NMR (300 MHz, $CDCl_3$) δ= 2.37 (s, 3H, CH_3), 2.45 (s, 3H, CH_3), 6.86-6.91 (m, 3H, CH), 6.93-6.98 (m, 2H, CH), 7.06-7.14 (m, 4H, CH), 7.25-7.29 (m, 2H, CH), 7.34-7.37 (m, 1H, CH); ^{13}C NMR (75 MHz, $CDCl_3$) δ= 23.2, 125.2, 125.8, 126.6, 128.5, 128.6, 128.9, 129.6, 130.6, 130.7, 130.8, 131.0, 132.7, 133.4, 134.4, 136.2, 136.6, 137.4, 146.3, 155.8, 156.1; HRMS (ESI+) calcd. for $C_{25}H_{19}Cl_3N$ [M+H]+ 438.0583; found 438.0579. **13** * HCl: white solid, mp 254-255.5°C, crystallization from dichloromethane/heptane. CCDC-1528679 contains the supplementary crystallographic data for this paper. These data can be obtained free of charge from The Cambridge Crystallographic Data Centre via www.ccdc.cam.ac.uk/data_request/cif.

(syn, syn)-3,4,5-Tris(2-chlorophenyl)-2,6-dimethylpyridine (**14**). White solid, yield 15%, mp 108 °C; R_f 0.17 (20% ethyl acetate in hexane); ^{1}H NMR (300 MHz, $CDCl_3$) δ= 2.42 (s, 6H, CH), 6.86-7.13 (m, 10H, CH), 7.30-7.33 (m, 2H, CH); ^{13}C NMR (75 MHz, $CDCl_3$) δ= 23.5, 124.6, 125.9, 127.7, 128.5, 128.8, 129.5, 129.8, 131.3, 132.7, 133.3, 133.8, 134.8, 135.7, 136.6, 146.7, 156.2; HRMS (ESI+) calcd. for $C_{25}H_{19}Cl_3N$ [M+H]+ 438.0583; found 438.0579, crystallization from ethyl acetate. CCDC-1528721 contains the supplementary crystallographic data for this paper. These data can be obtained free of charge from The Cambridge Crystallographic Data Centre via www.ccdc.cam.ac.uk/data_request/cif.

(anti)-(aR,aS)-3-Bromo-4,5-bis(2-chlorophenyl)-2,6-dimethylpyridine (**15**). White solid, yield 48%, mp 110.5 °C; R_f 0.42 (20% ethyl acetate in hexane); ^{1}H NMR (300 MHz, $CDCl_3$) δ= 2.62 (s, 3H, CH), 2.79 (s, 6H, CH), 7.07-7.18 (m, 6H, CH), 7.26-7.28 (m, 1H, CH), 7.29-7.30 (m, 1H, CH); ^{13}C NMR (75 MHz, $CDCl_3$) δ= 22.7, 25.8, 120.2,

126.6, 126.8, 128.9, 128.9, 129.0, 129.3, 126.4, 129.9, 132.4, 132.5, 133.3, 136.8, 138.0, 147.6, 155.0, 156.6; HRMS (ESI+) calcd. for $C_{19}H_{15}BrCl_2N$ [M+H]+ 407.9742; found 407.9748. **15** * picrate: yellow solid, mp 214-216°C, crystallization from dichloromethane/methanol. CCDC-1528655 contains the supplementary crystallographic data for this paper. These data can be obtained free of charge from The Cambridge Crystallographic Data Centre via www.ccdc.cam.ac.uk/data_request/cif.

(syn)-(aR,aS)-3-Bromo-4,5-bis(2-chlorophenyl)-2,6-dimethylpyridine (**16**). White solid, yield 13%, mp 154 °C; R_f 0.404 (20% ethyl acetate in hexane); ^{1}H NMR (300 MHz, $CDCl_3$) δ= 2.29 (s, 3H, CH_3), 2.80 (s, 3H, CH_3), 6.97-7.15 (m, 6H, CH), 7.25-7.31 (m, 2H, CH); ^{13}C NMR (75 MHz, $CDCl_3$) δ= 23.1, 26.2, 120.8, 125.9, 126.2, 129.2, 129.4, 129.7, 129.9, 131.5, 132.4, 132.7, 133.3, 134.3, 136.1, 137.4, 147.9, 155.4, 156.8; HRMS (ESI+) calcd. for $C_{19}H_{15}BrCl_2N$ [M+H]+ 407.9742; found 407.9748, crystallization from ethyl acetate. CCDC-1528653 contains the supplementary crystallographic data for this paper. These data can be obtained free of charge from The Cambridge Crystallographic Data Centre via www.ccdc.cam.ac.uk/data_request/cif.

(anti)-(aR,aS)-4-Bromo-3,5-bis(2-chlorophenyl)-2,6-dimethylpyridine (**17**). White solid, yield 18%, mp 229 °C; R_f 0.34 (20% ethyl acetate in hexane); ^{1}H NMR (300 MHz, $CDCl_3$) δ= 2.34 (s, 6H, CH_3), 7.24-7.28 (m, 2H, CH), 7.35-7.41 (m, 4H, CH), 7.49-7.54 (m, 2H, CH); ^{13}C NMR (75 MHz, $CDCl_3$) δ= 23.7, 127.2, 129.6, 129.7, 131.1, 133.1, 133.5, 136.5, 138.0, 156.5; HRMS (ESI+) calcd. for $C_{19}H_{15}BrCl_2N$ [M+H]+ 407.9742; found 407.9748, crystallization from ethyl acetate. CCDC-1528652 contains the supplementary crystallographic data for this paper. These data can be obtained free of charge from The Cambridge Crystallographic Data Centre via www.ccdc.cam.ac.uk/data_request/cif.

^{1}H NMR spectrum of **6** (300 MHz, $CDCl_3$)

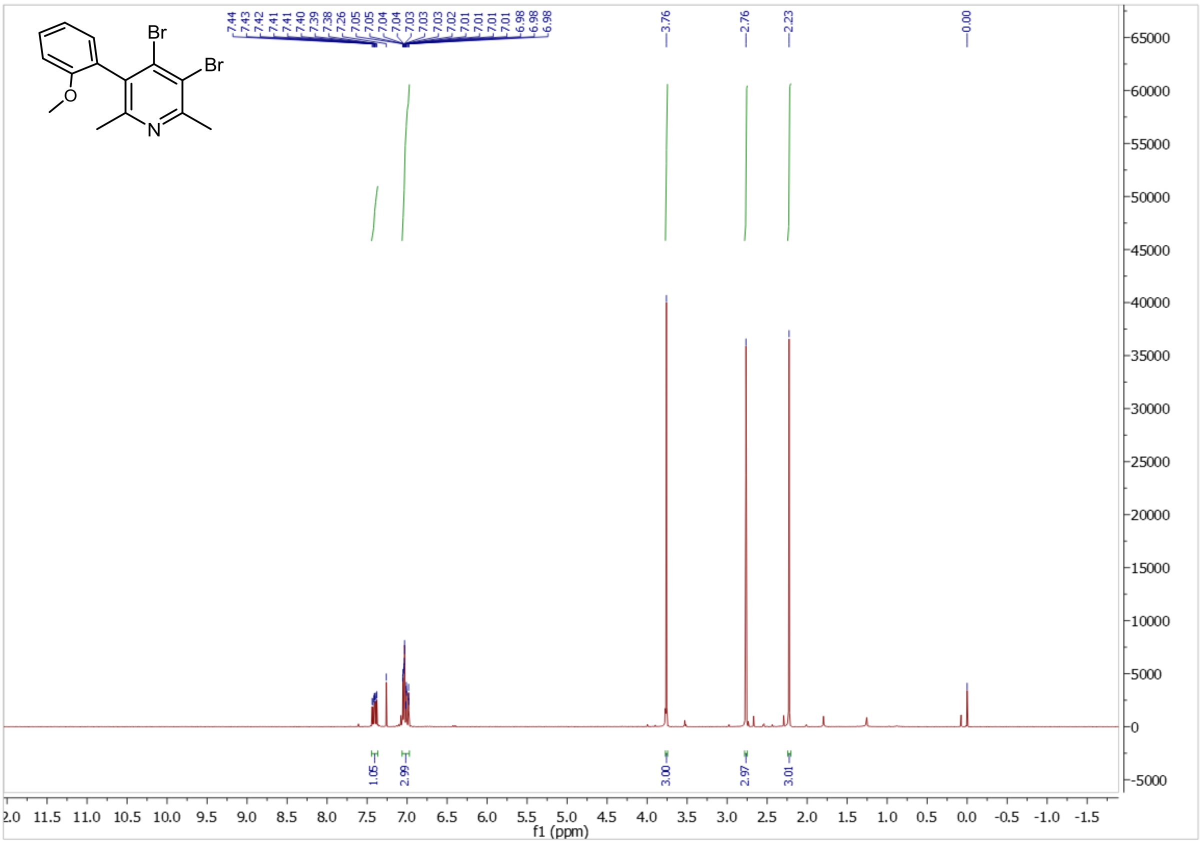

^{13}C NMR spectrum of **6** (75 MHz, $CDCl_3$)

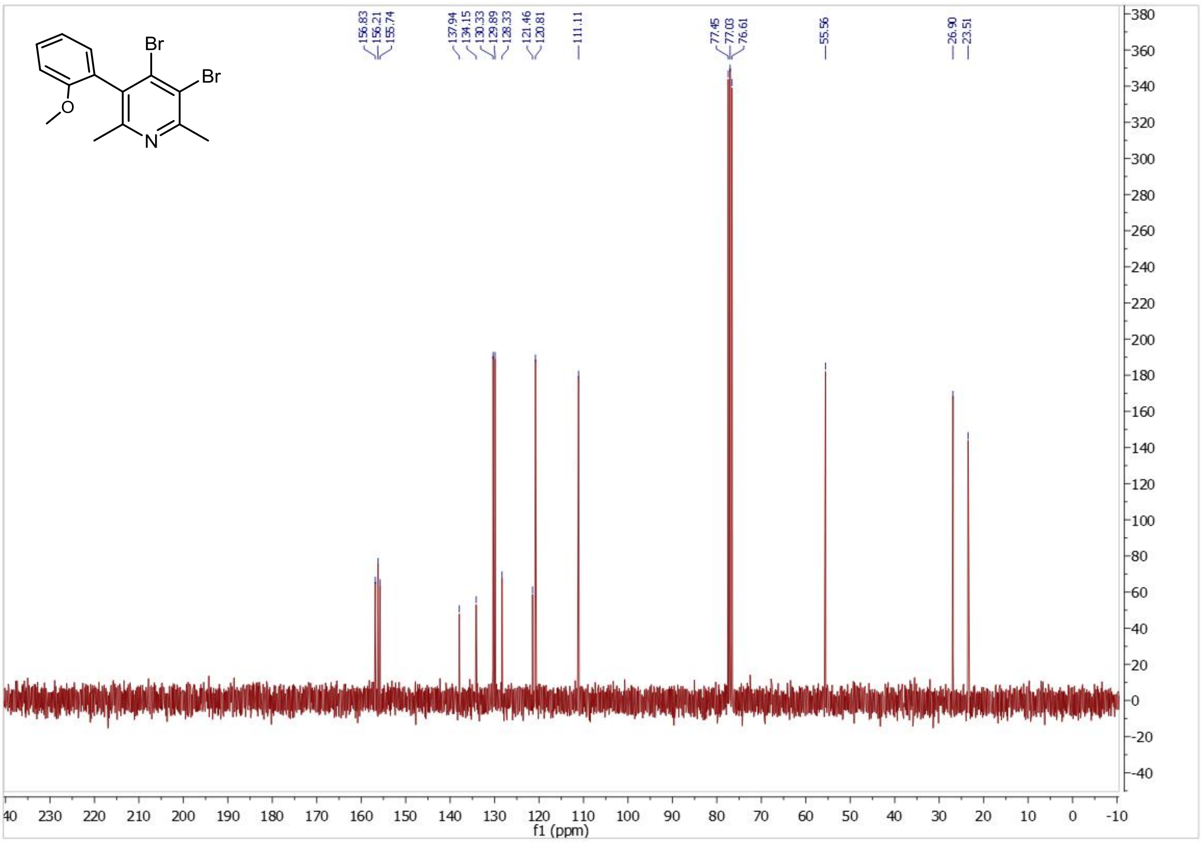

^{1}H NMR spectrum of **7** (300 MHz, $CDCl_3$)

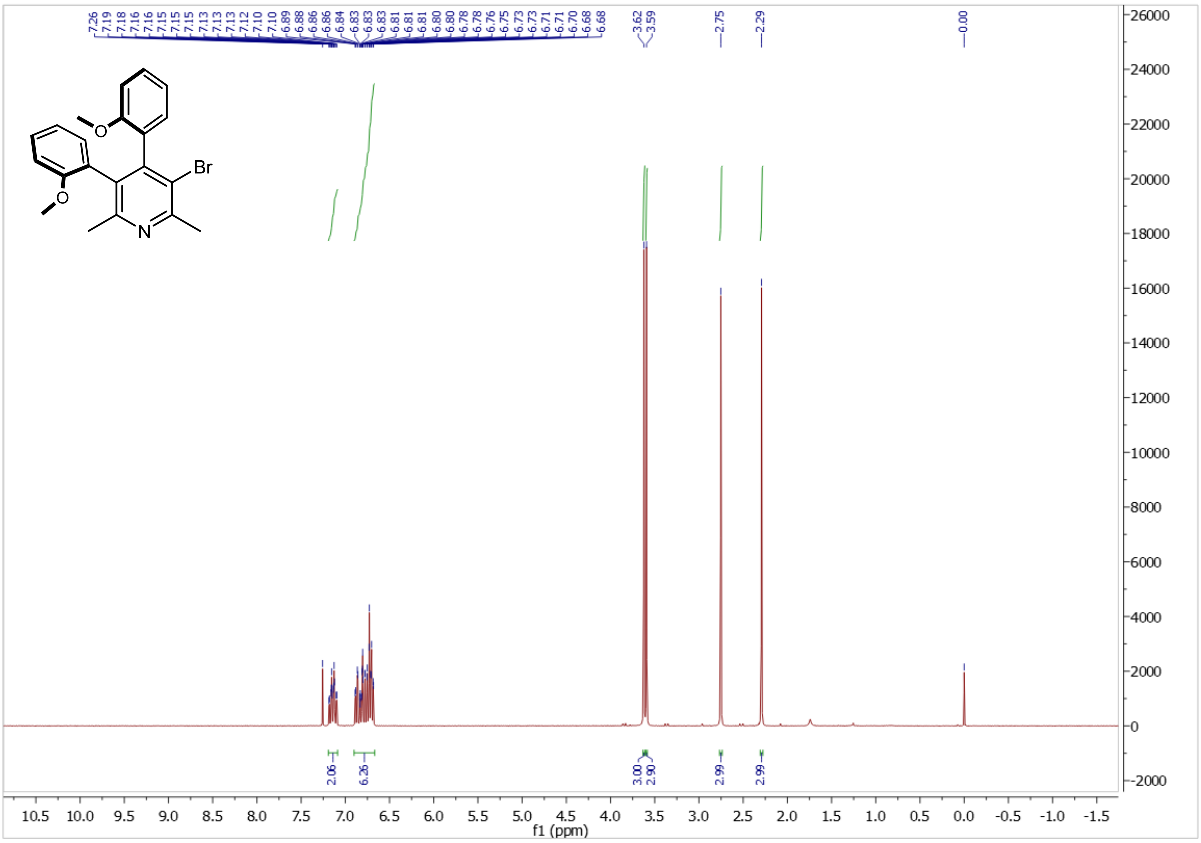

^{13}C NMR spectrum of **7** (75 MHz, $CDCl_3$)

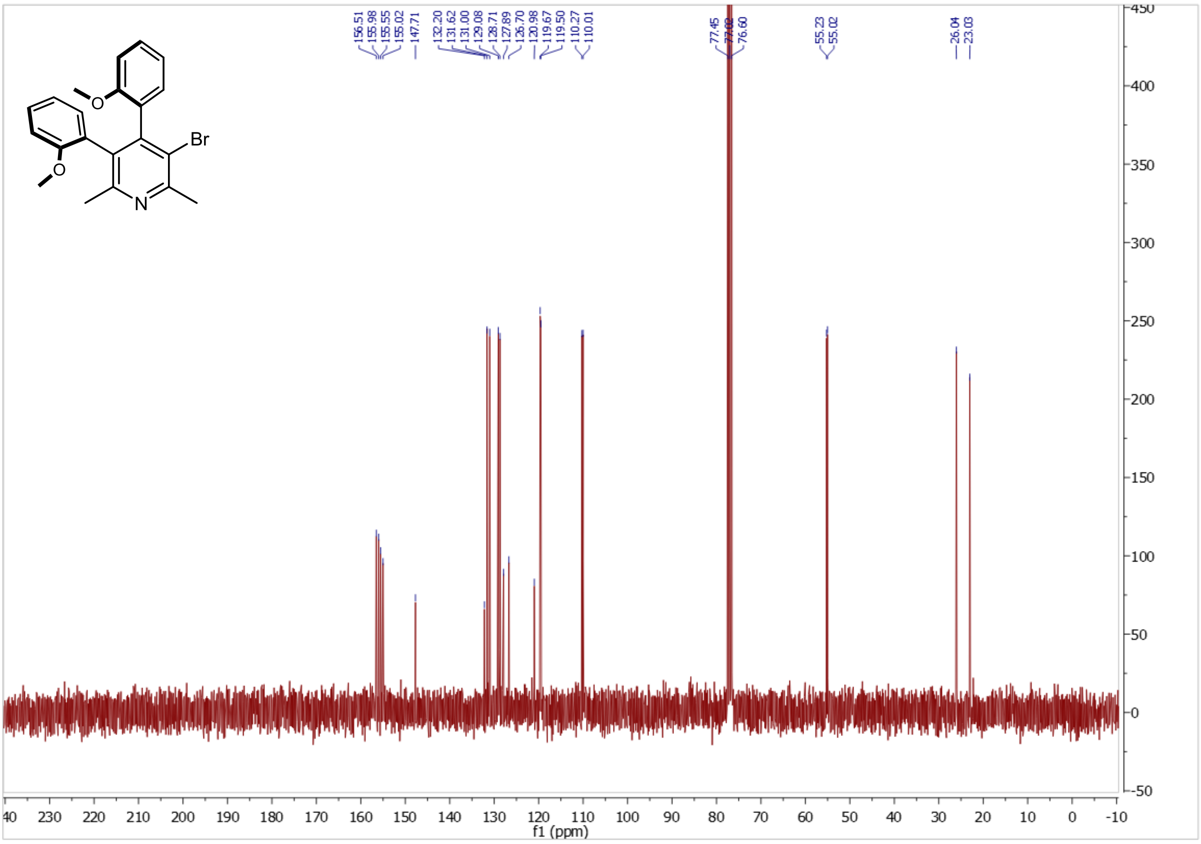

^{1}H NMR spectrum of **8** (300 MHz, $CDCl_3$)

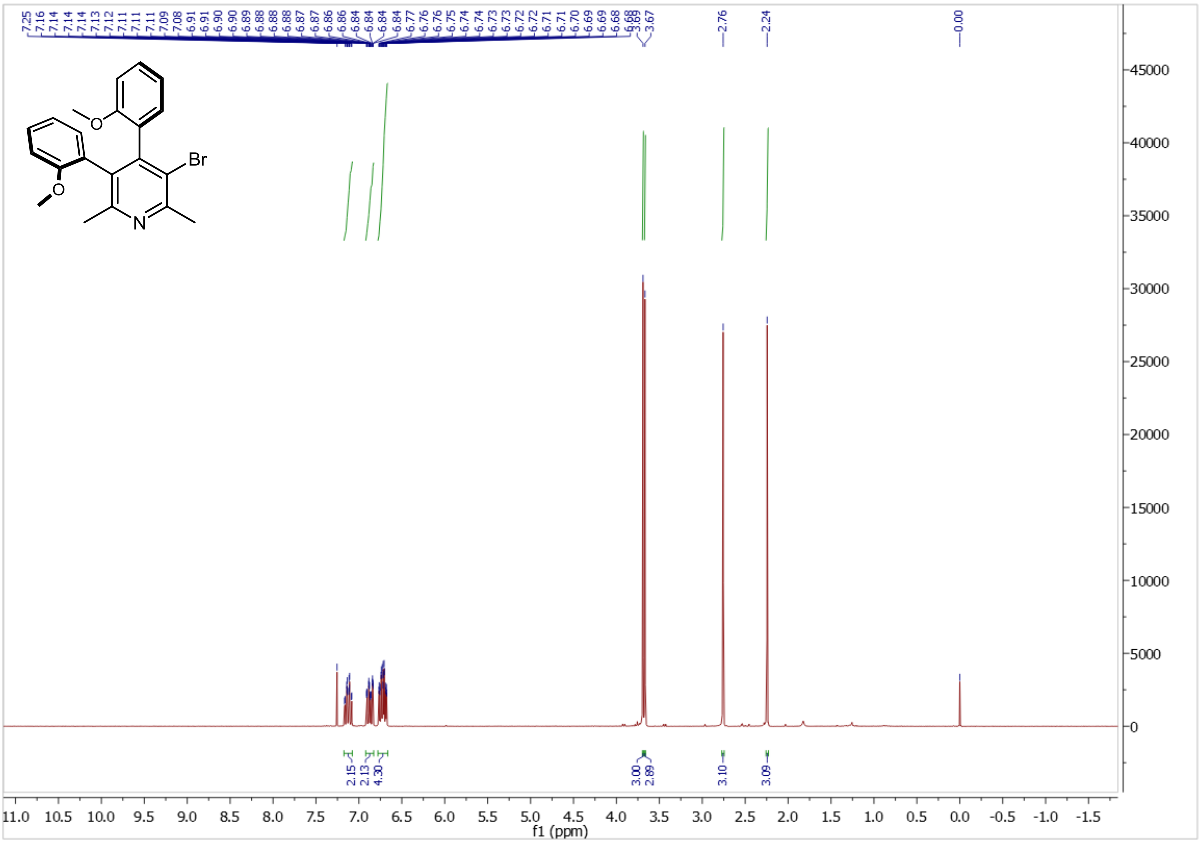

^{13}C NMR spectrum of **8** (75 MHz, $CDCl_3$)

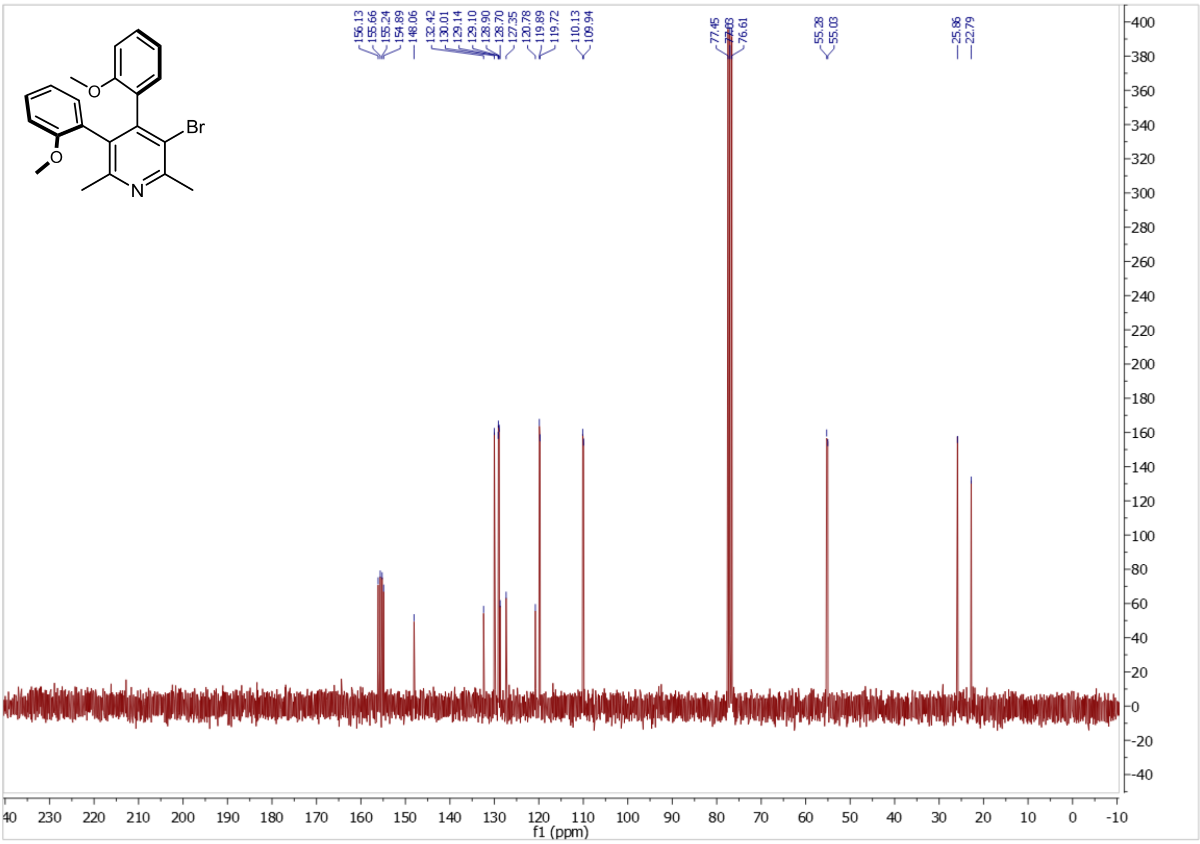

^{1}H NMR spectrum of **7** (300 MHz, DMSO)

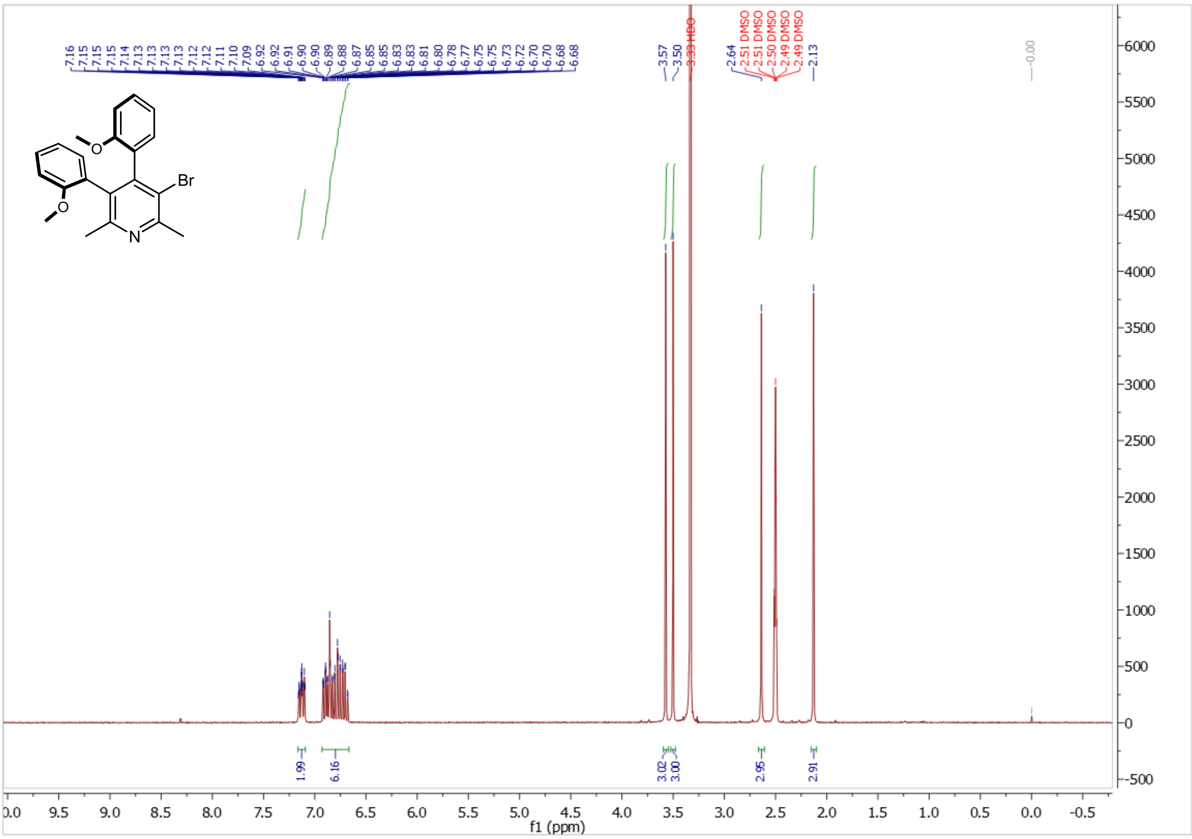

^{1}H NMR spectrum of **8** (300 MHz, DMSO)

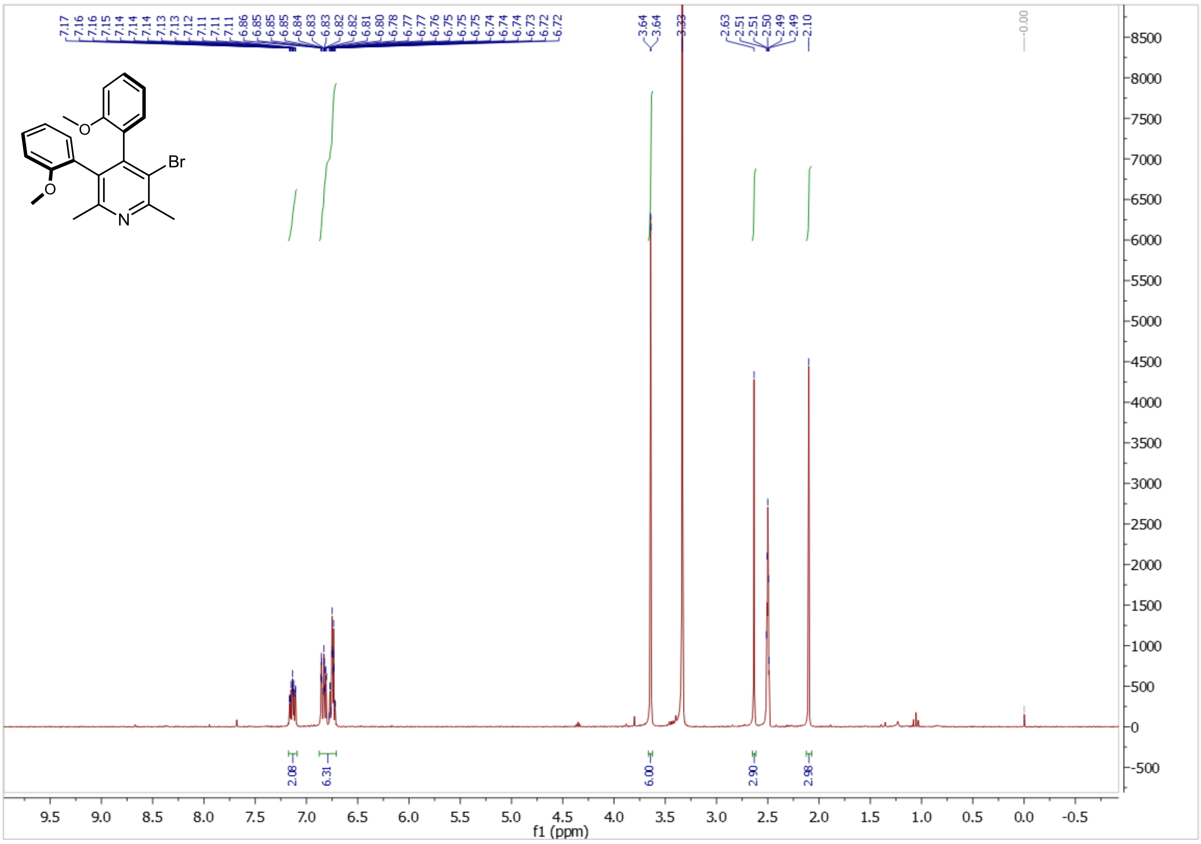

^{1}H NMR spectrum of **9** (300 MHz, $CDCl_3$)

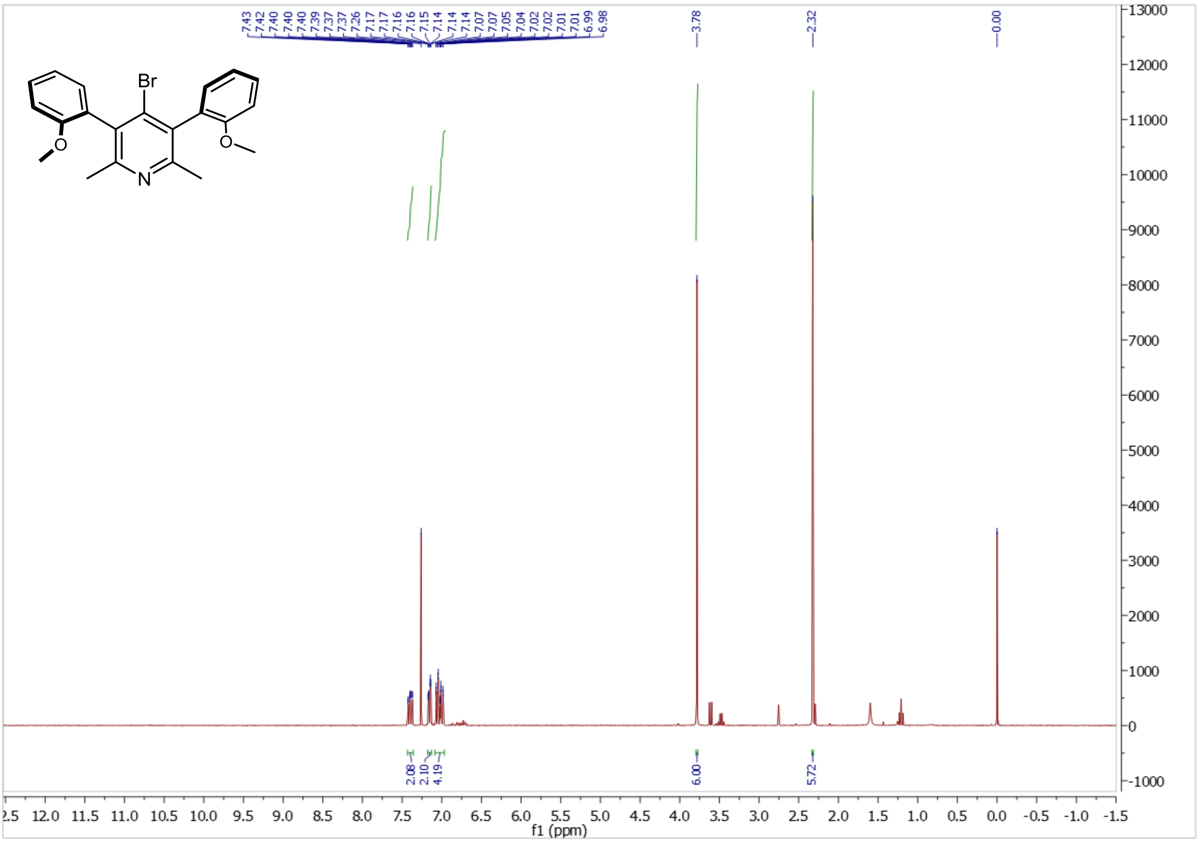

^{13}C NMR spectrum of **9** (75 MHz, $CDCl_3$)

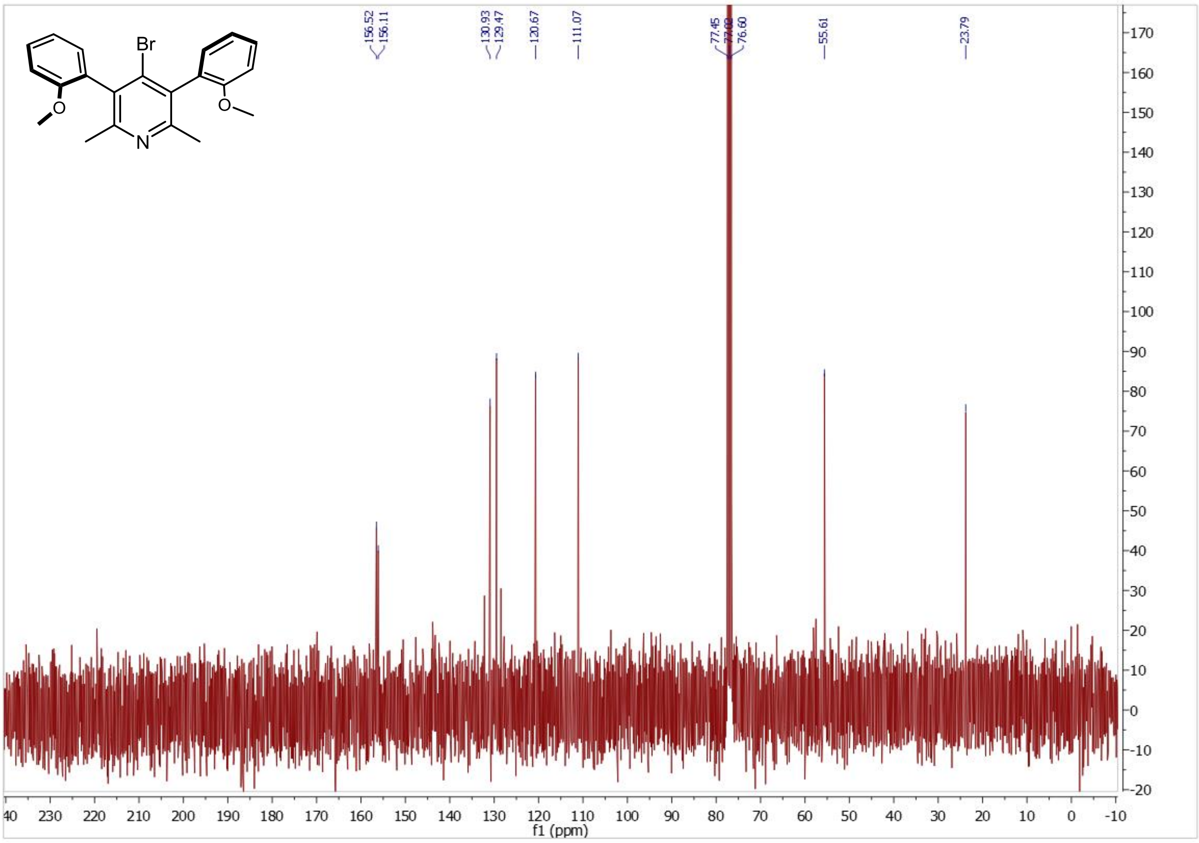

^{1}H NMR spectrum of **10** (300 MHz, $CDCl_3$)

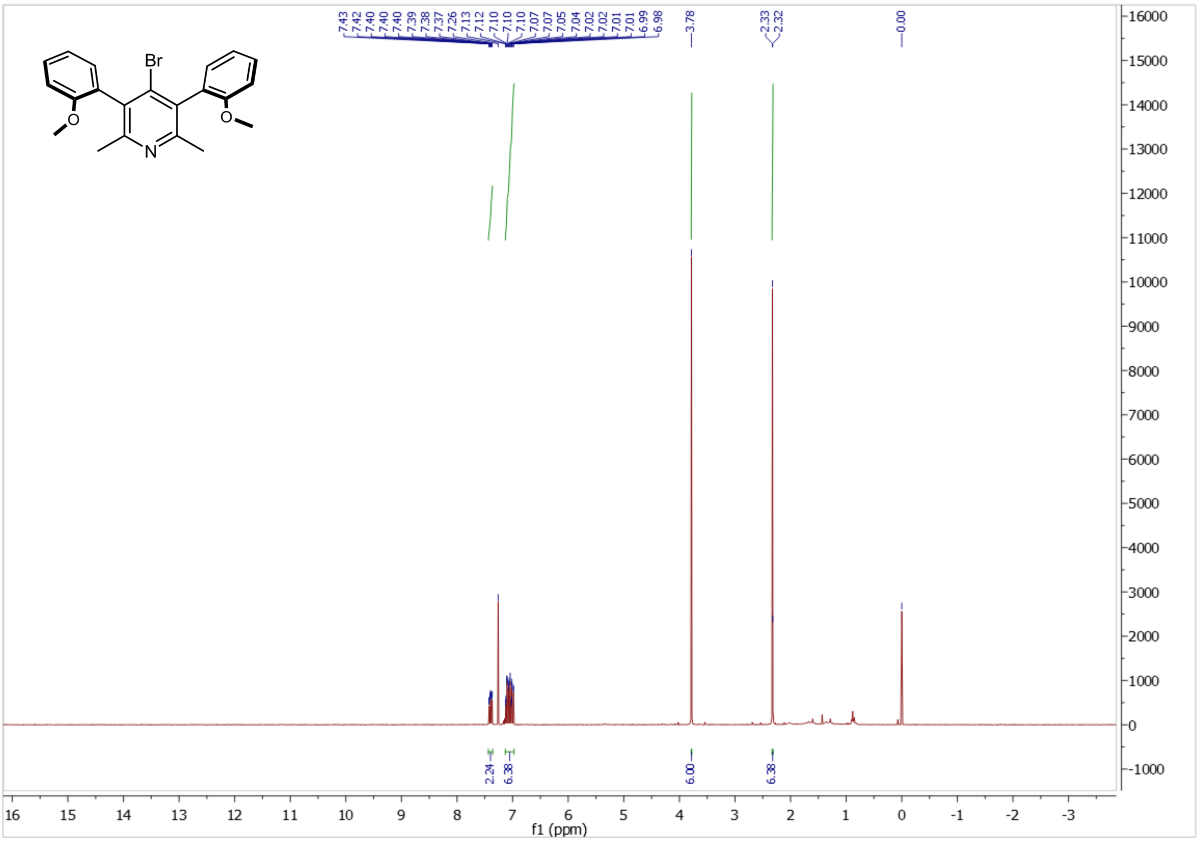

^{13}C NMR spectrum of **10** (75 MHz, $CDCl_3$)

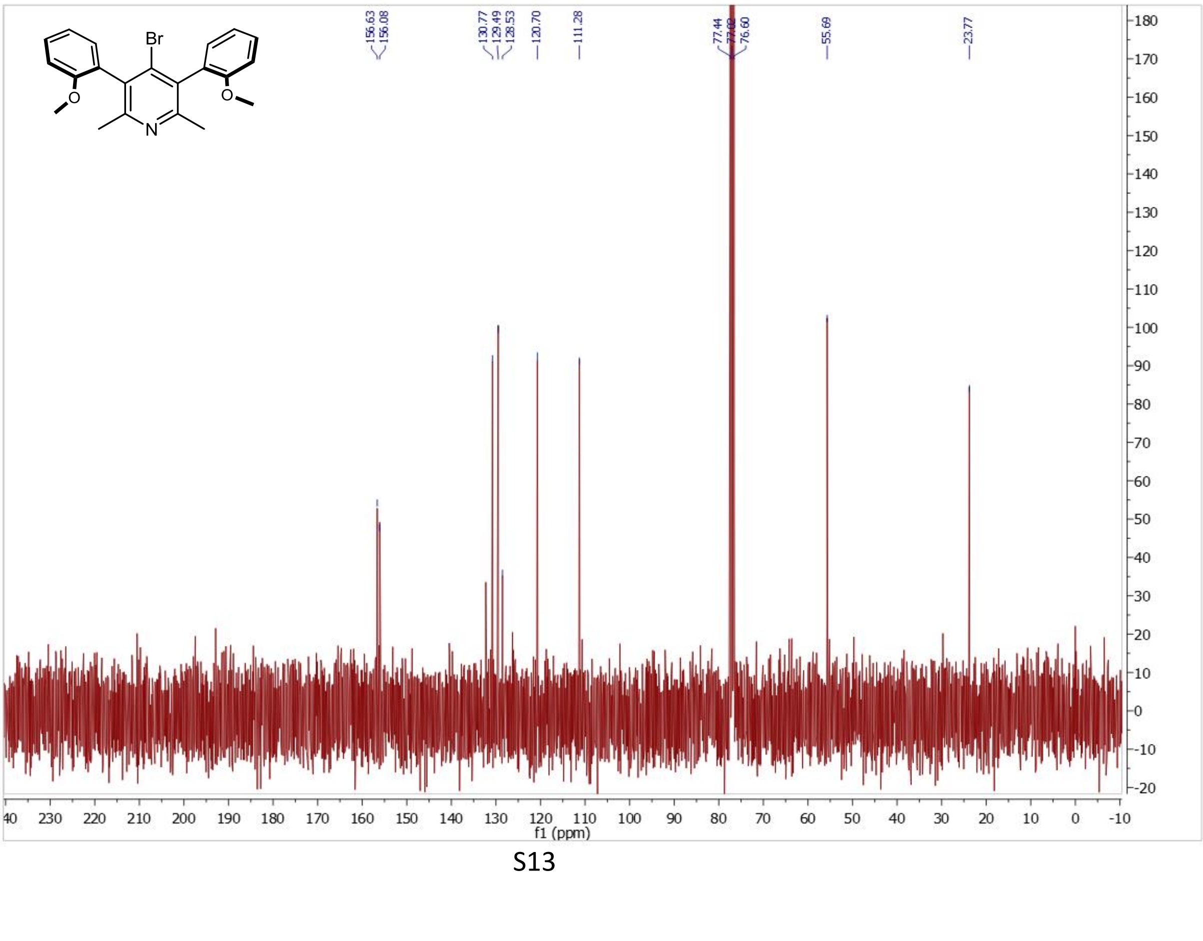

^{1}H NMR spectrum of **12** (300 MHz, $CDCl_3$)

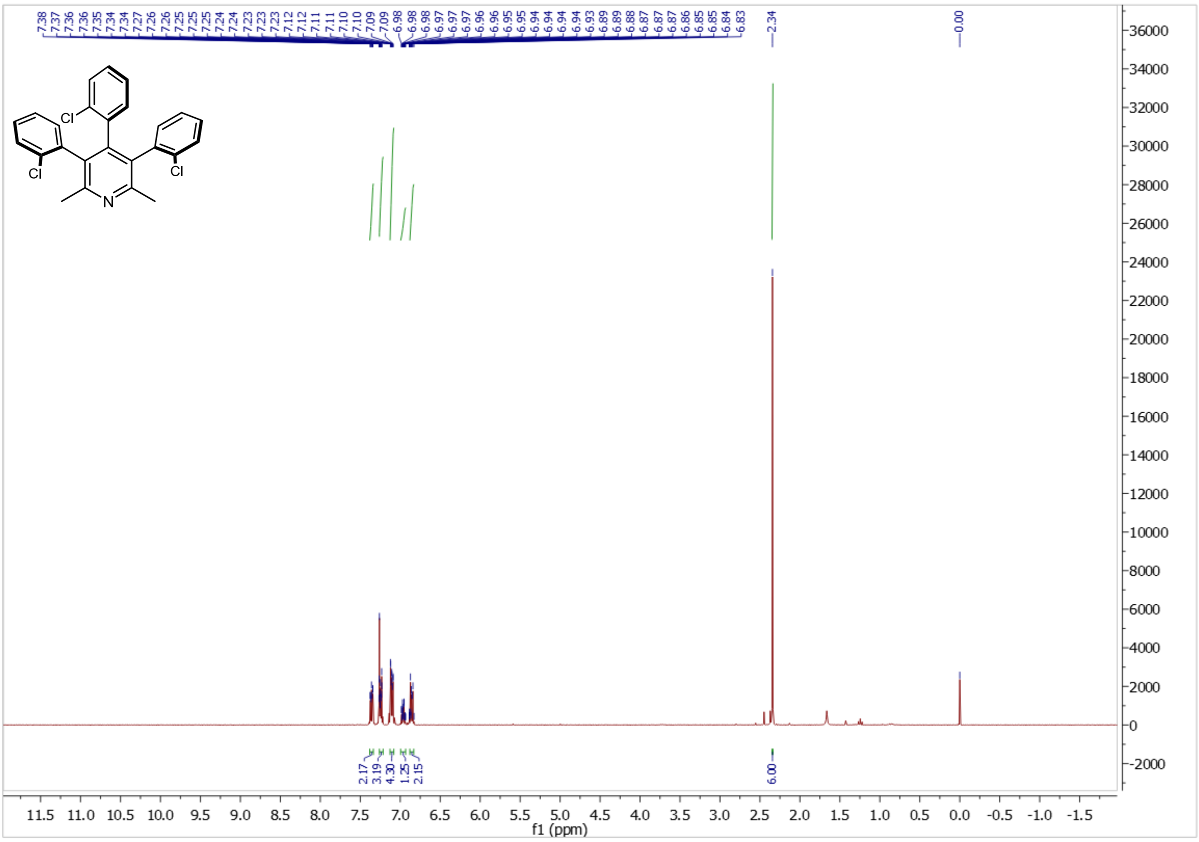

^{13}C NMR spectrum of **12** (75 MHz, $CDCl_3$)

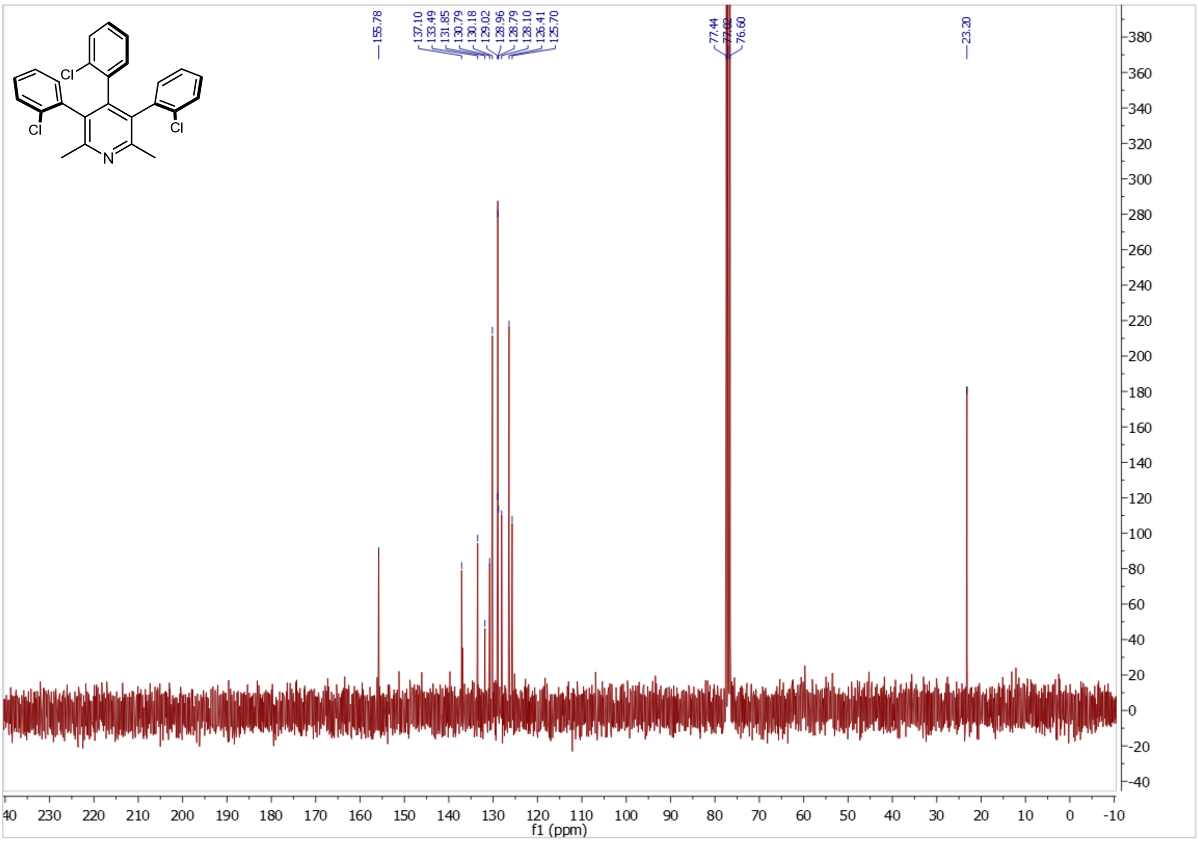

^{1}H NMR spectrum of **12** (300 MHz, DMSO) t=25°C

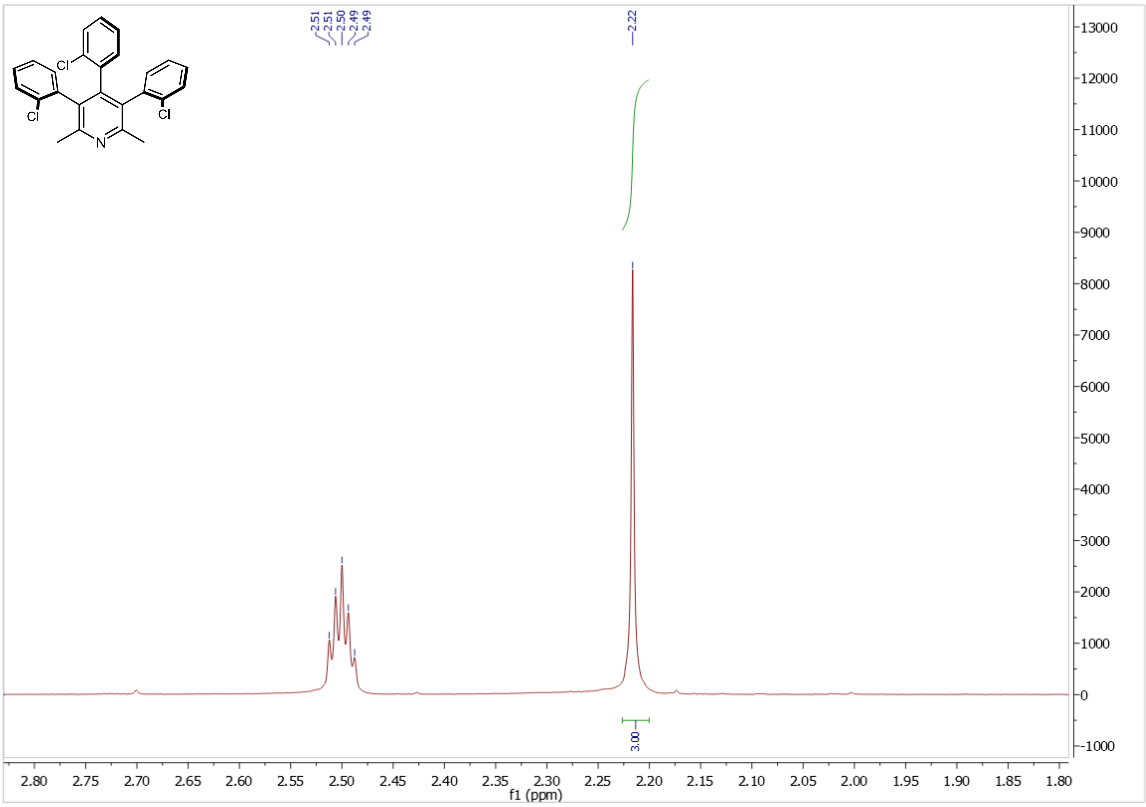

^{1}H NMR spectrum of **12** (300 MHz, DMSO) after 2 h heating at 120°C,

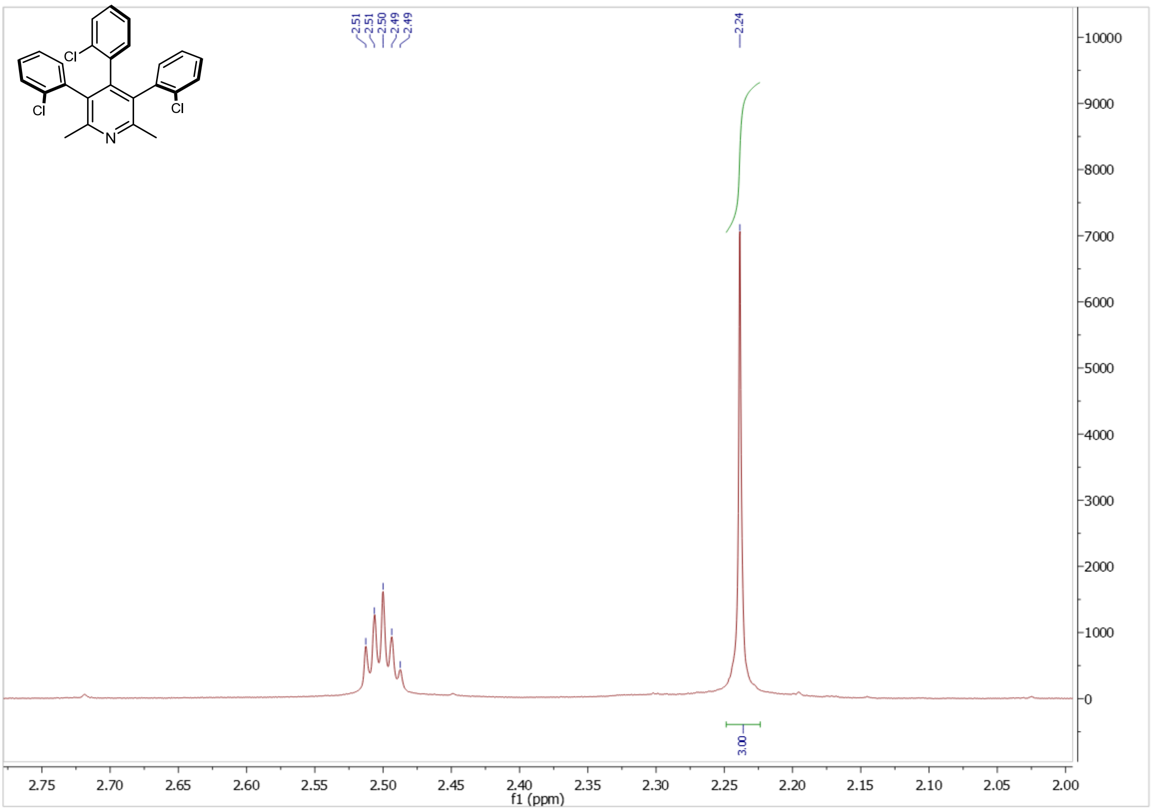

^{1}H NMR spectrum of **13** (300 MHz, $CDCl_3$)

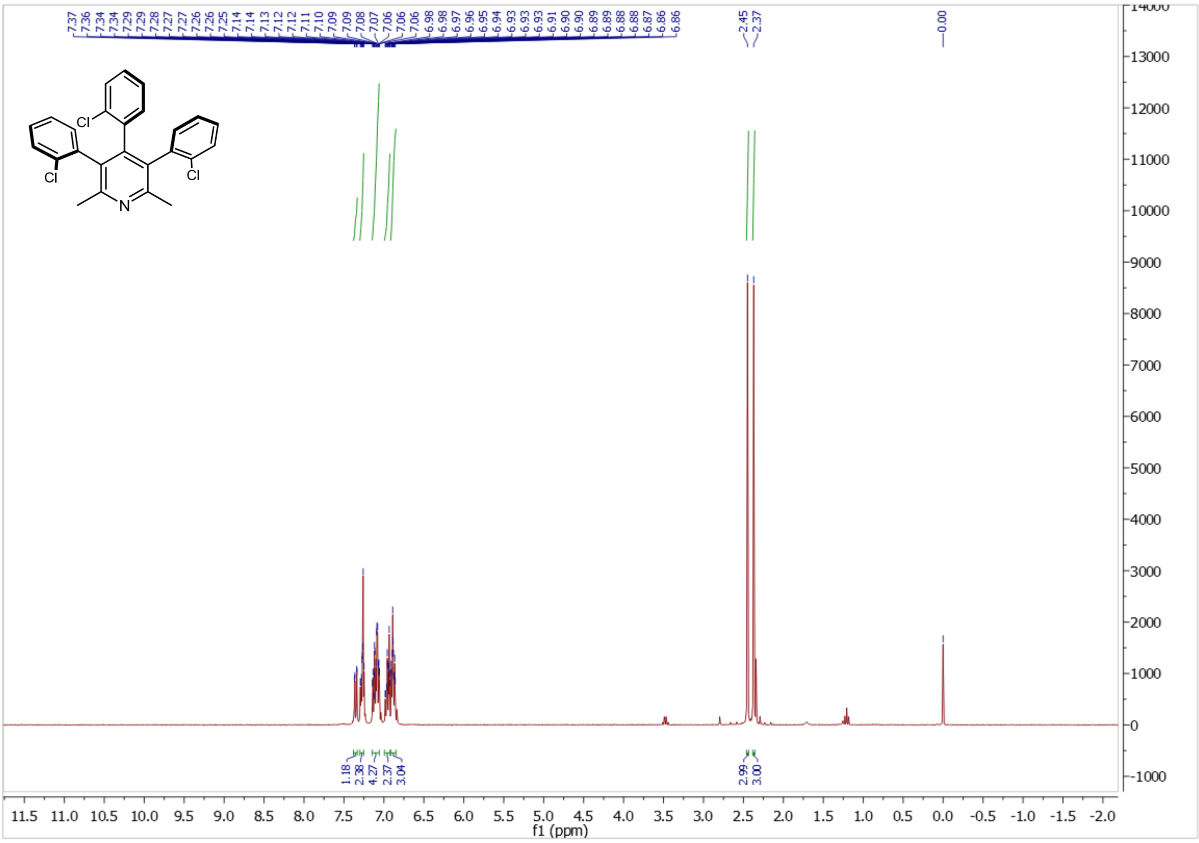

^{13}C NMR spectrum of **13** (75 MHz, $CDCl_3$)

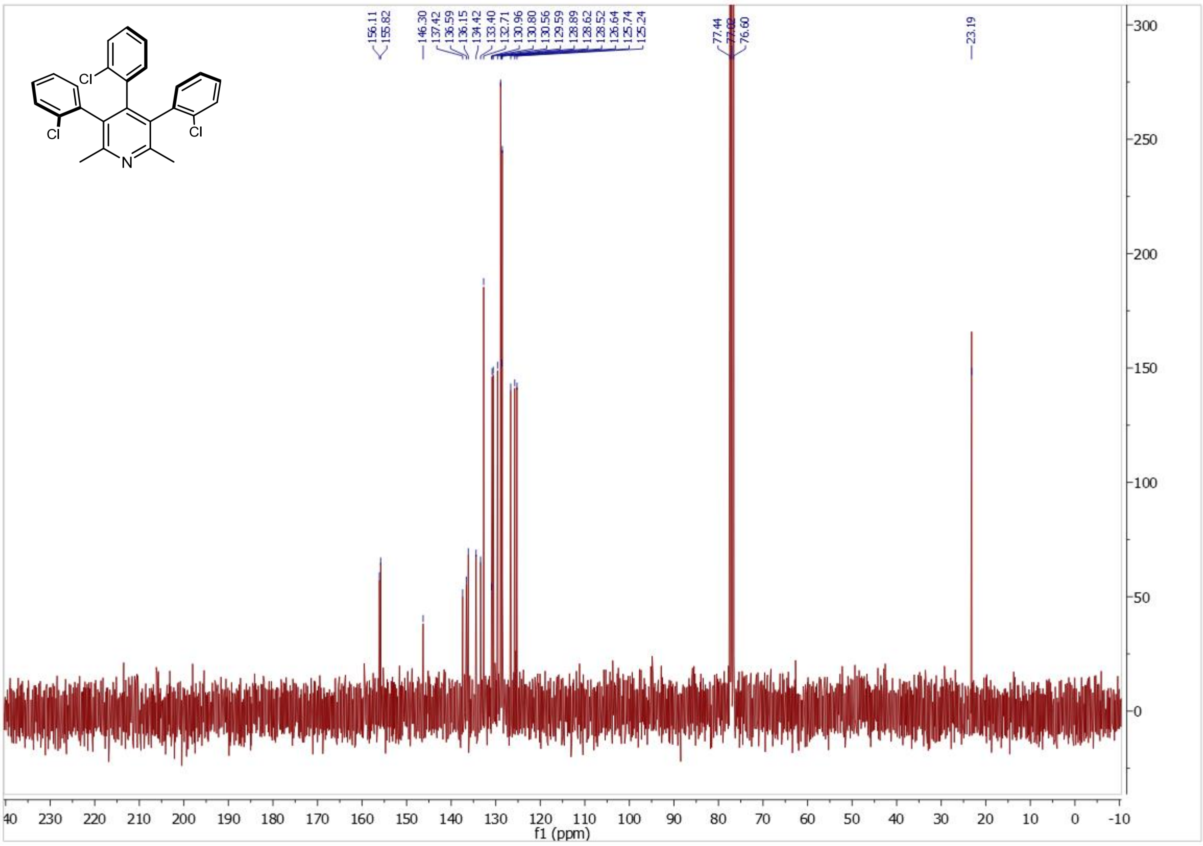

^{1}H NMR spectrum of **13** (300 MHz, DMSO) t=25°C

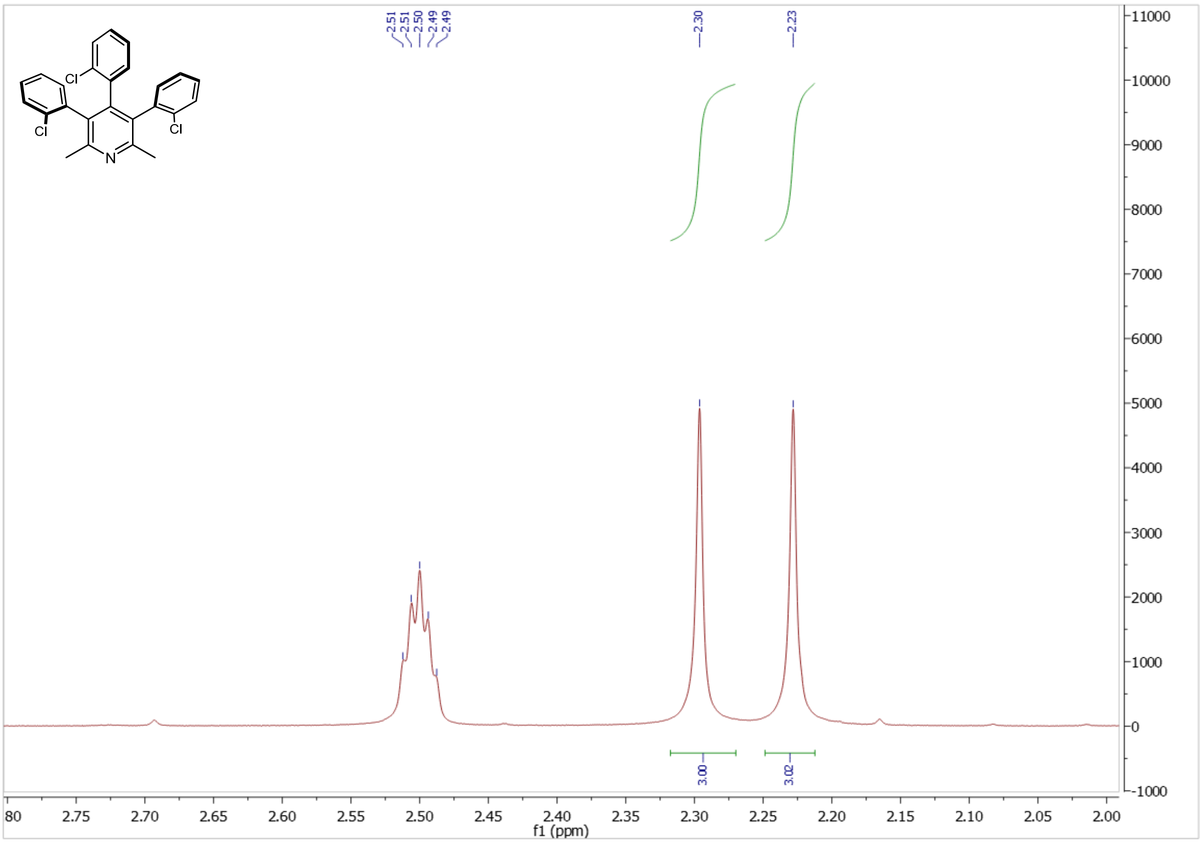

^{1}H NMR spectrum of **13** (300 MHz, DMSO) after 2 h heating at 120°C

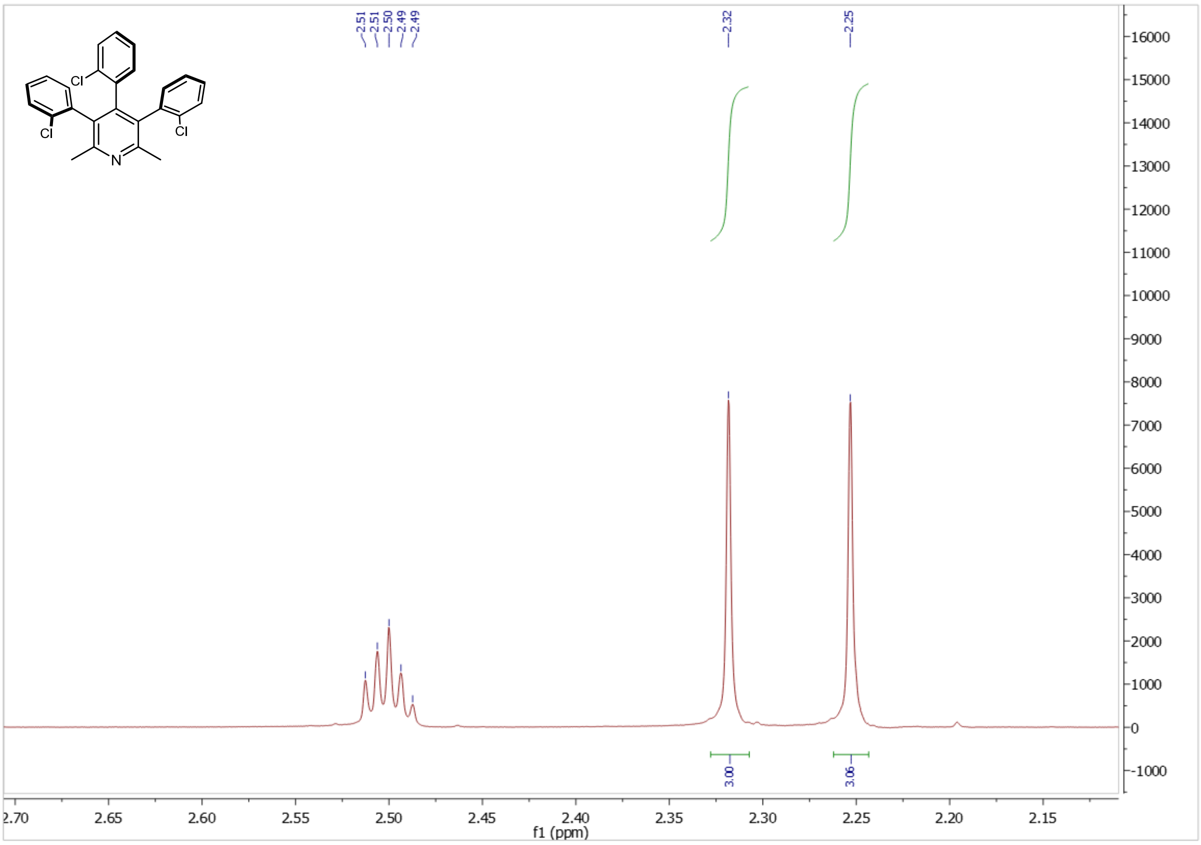

^{1}H NMR spectrum of **14** (300 MHz, $CDCl_3$)

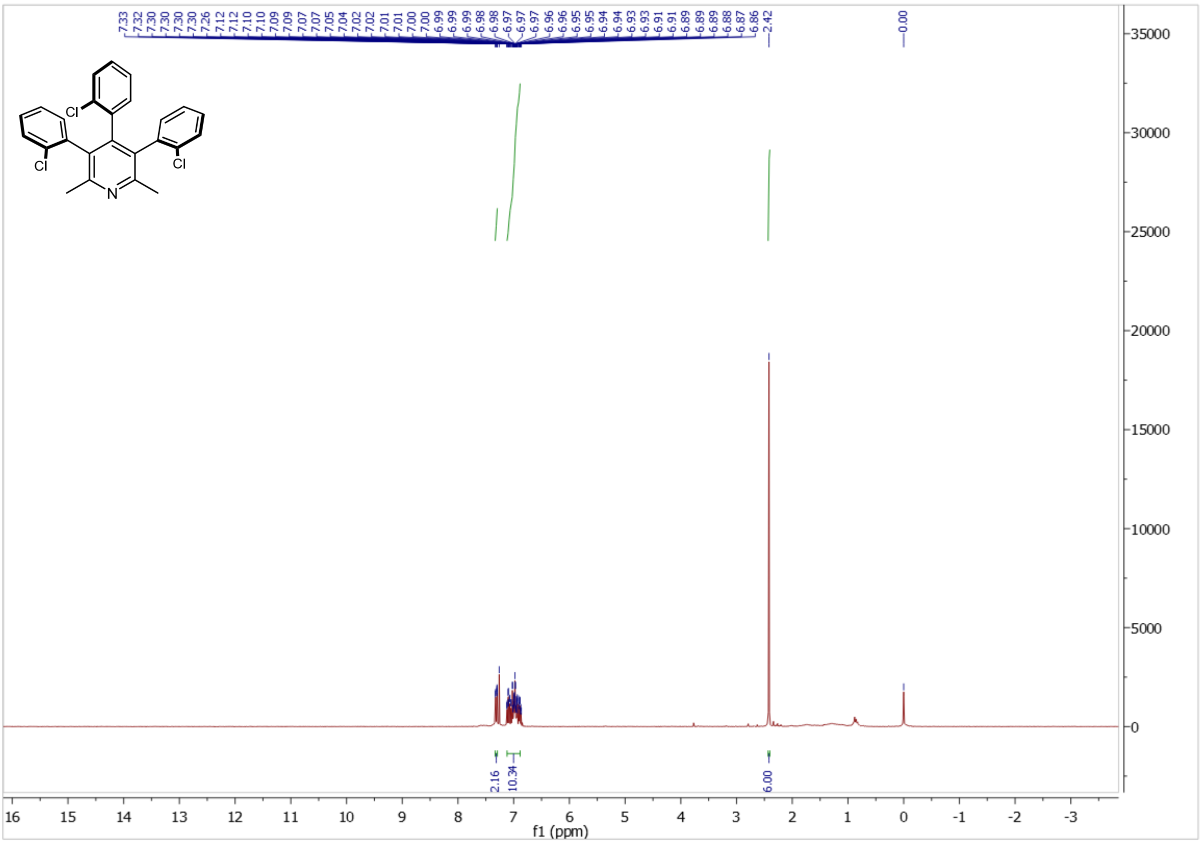

^{13}C NMR spectrum of **14** (75 MHz, $CDCl_3$)

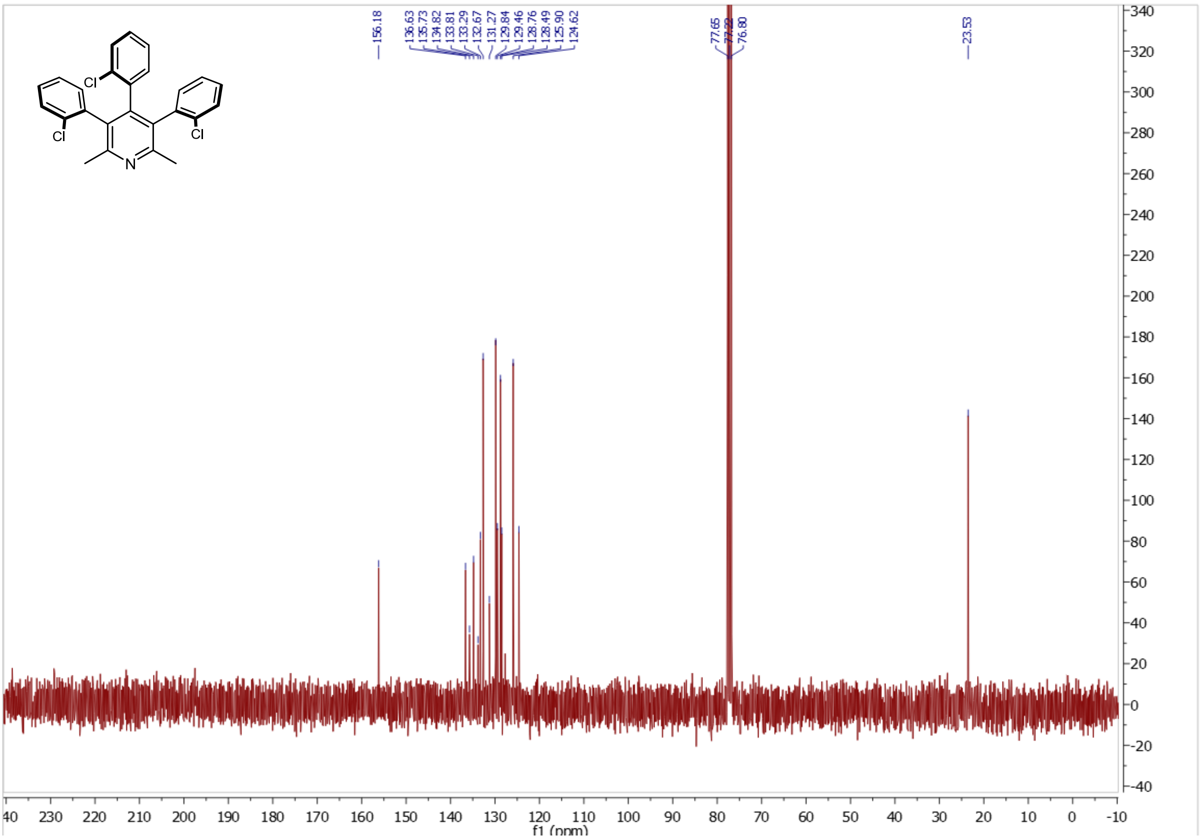

^{1}H NMR spectrum of **14** (300 MHz, DMSO) t=25°C

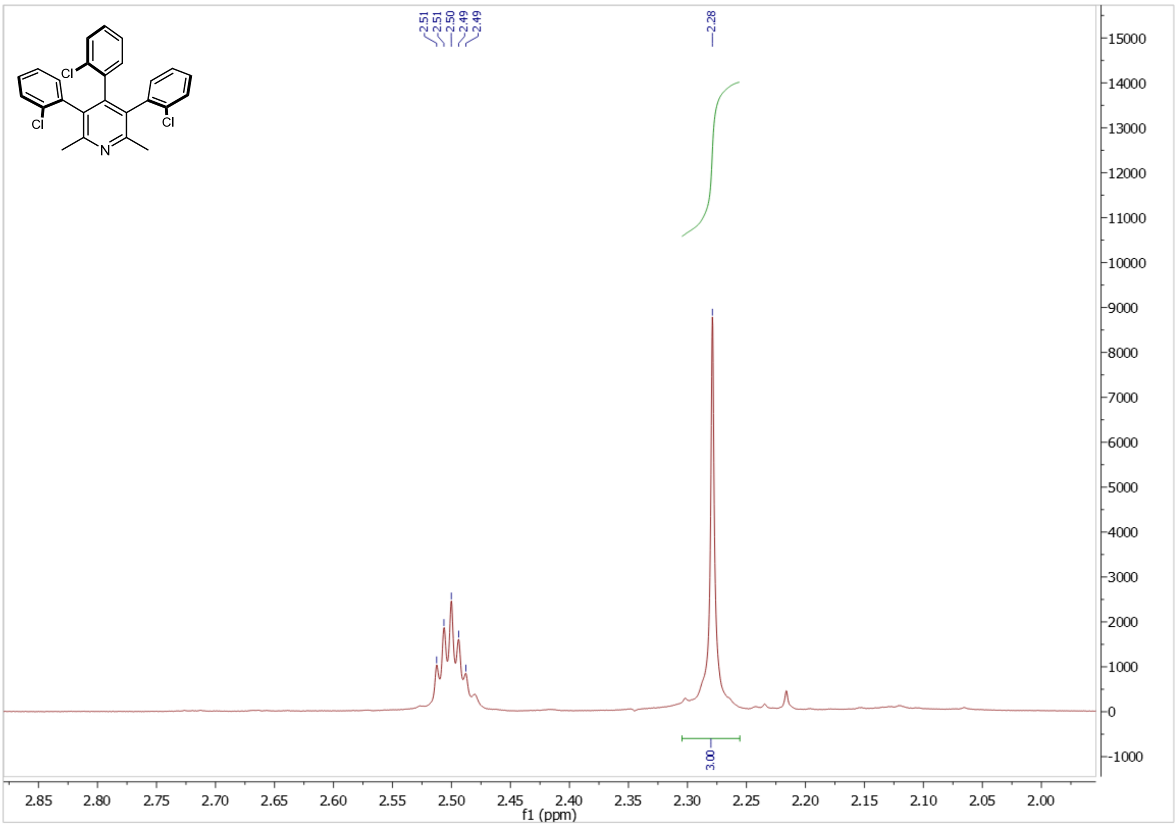

^{1}H NMR spectrum of **14** (300 MHz, DMSO) after 2 h heating at 120°C

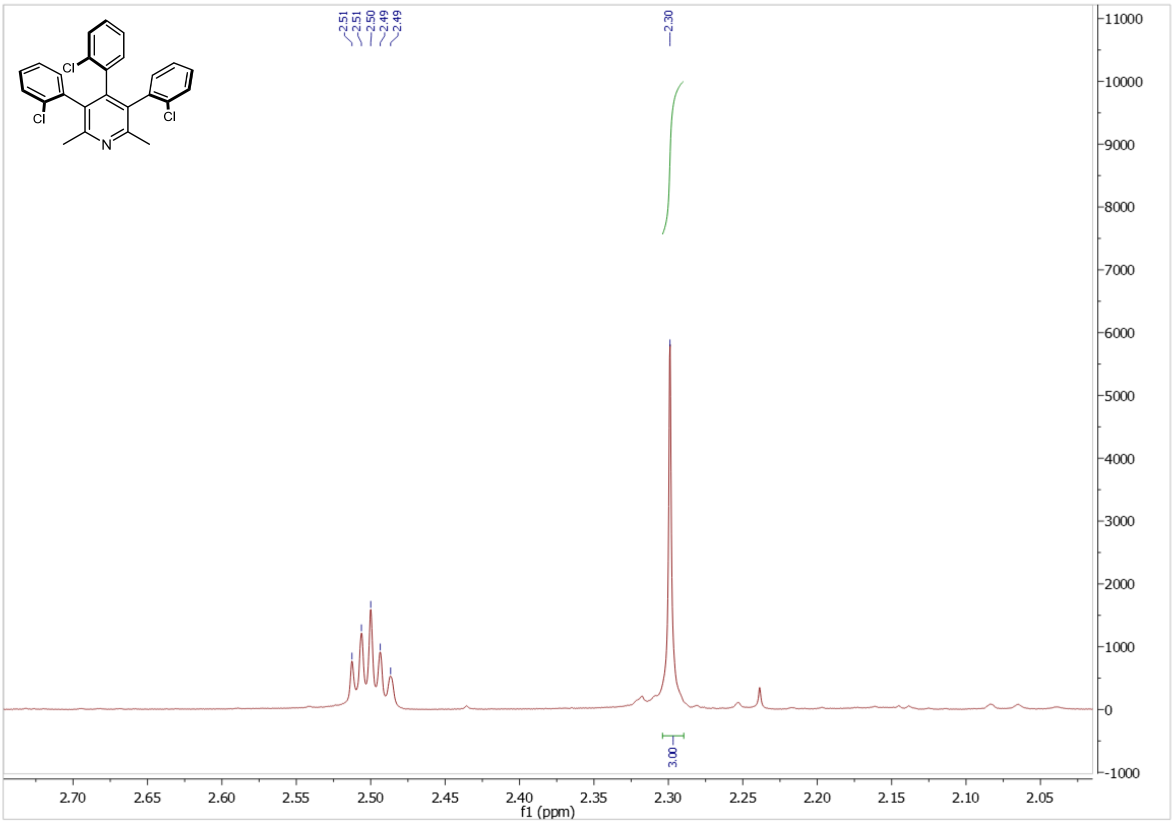

^{1}H NMR spectrum of **15** (300 MHz, $CDCl_3$)

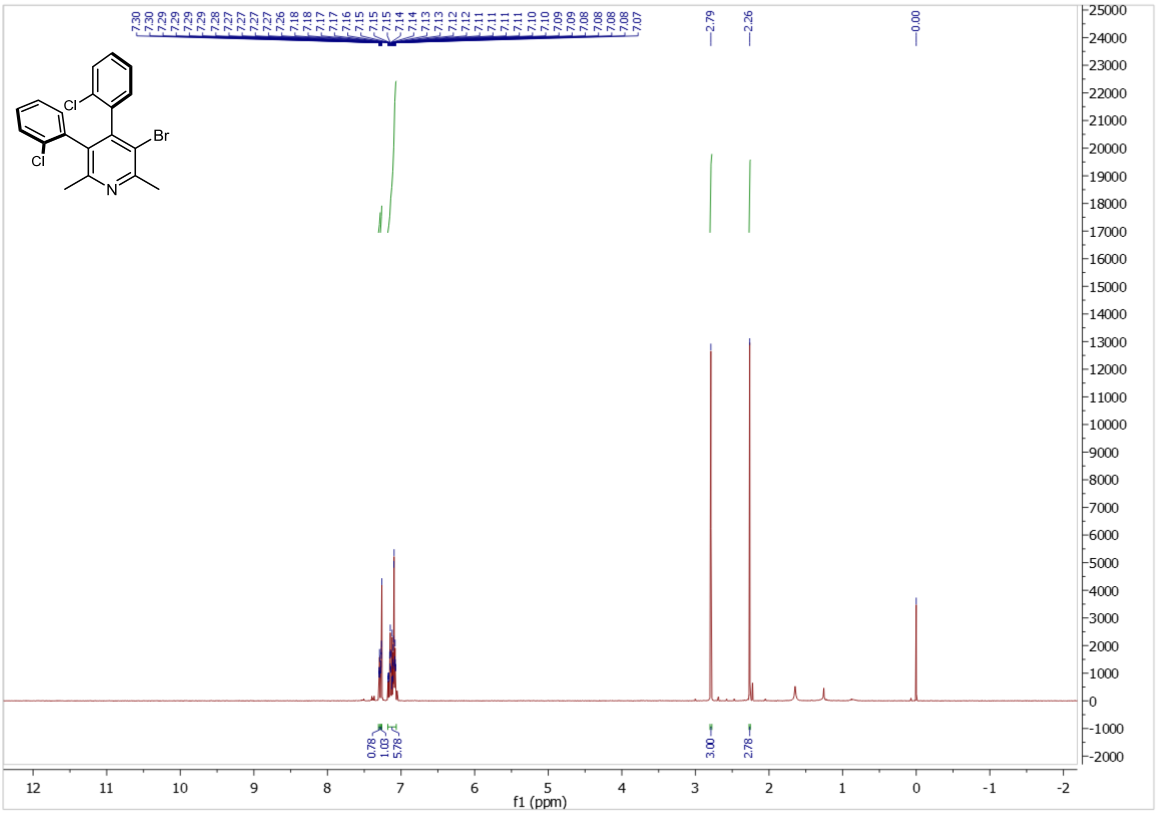

^{13}C NMR spectrum of **15** (75 MHz, $CDCl_3$)

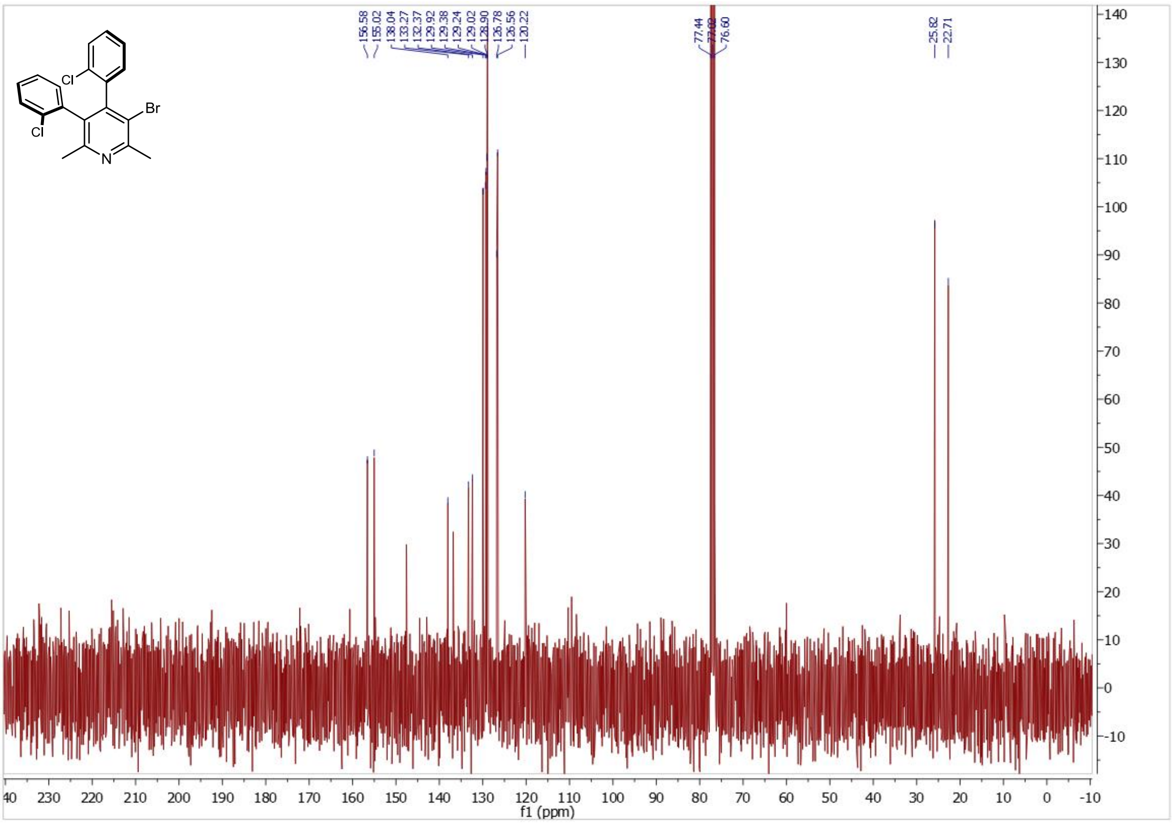

^{1}H NMR spectrum of **15** (300 MHz, DMSO) t=25°C

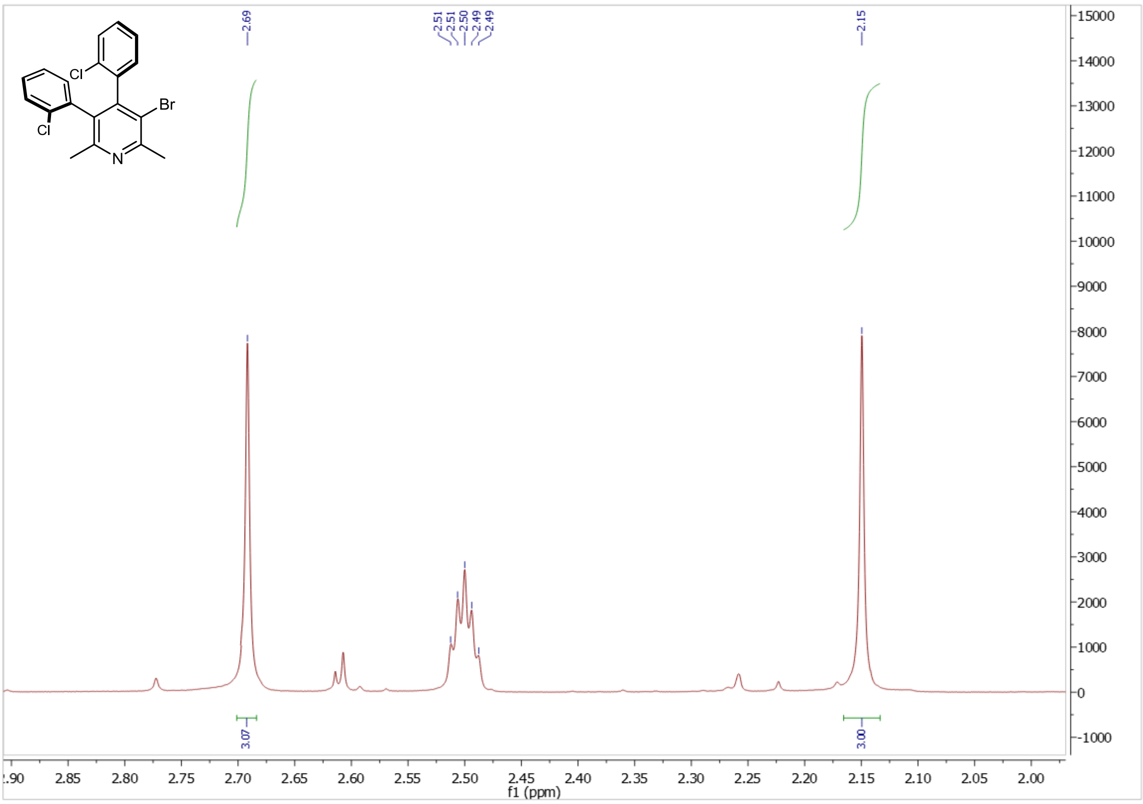

^{1}H NMR spectrum of **15** (300 MHz, DMSO) after 2 h heating at 120°C

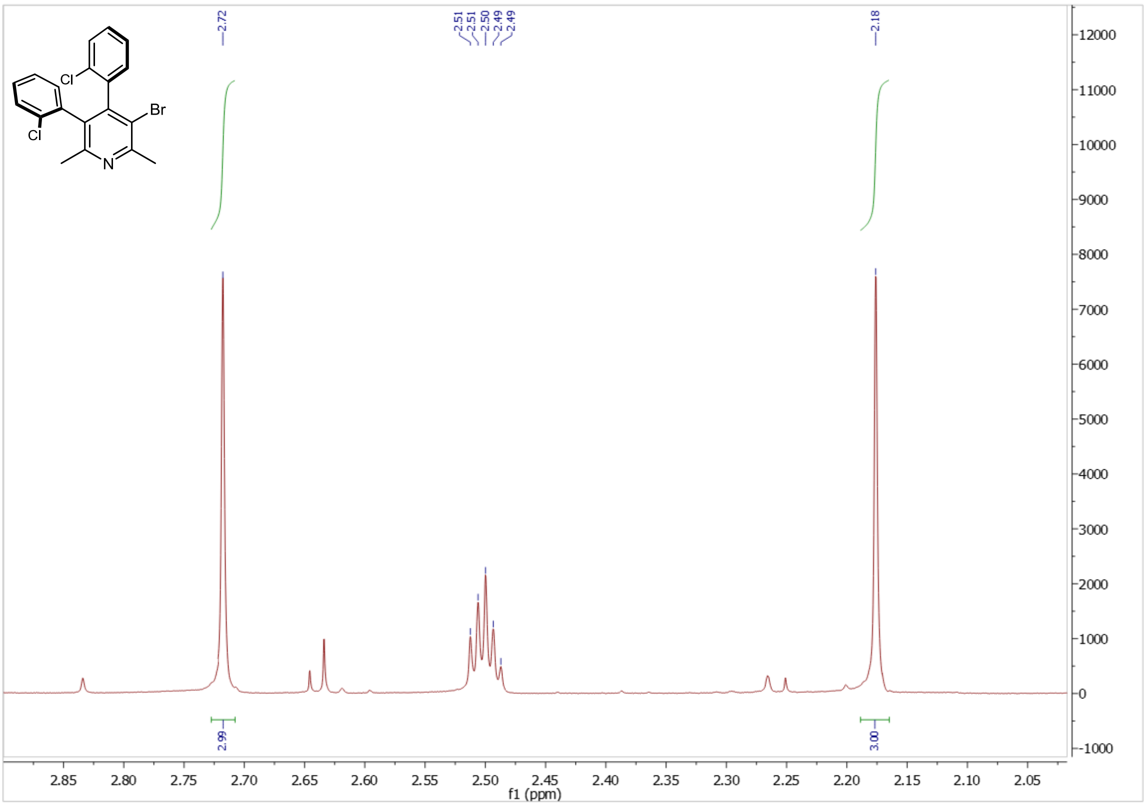

^{1}H NMR spectrum of **16** (300 MHz, $CDCl_3$)

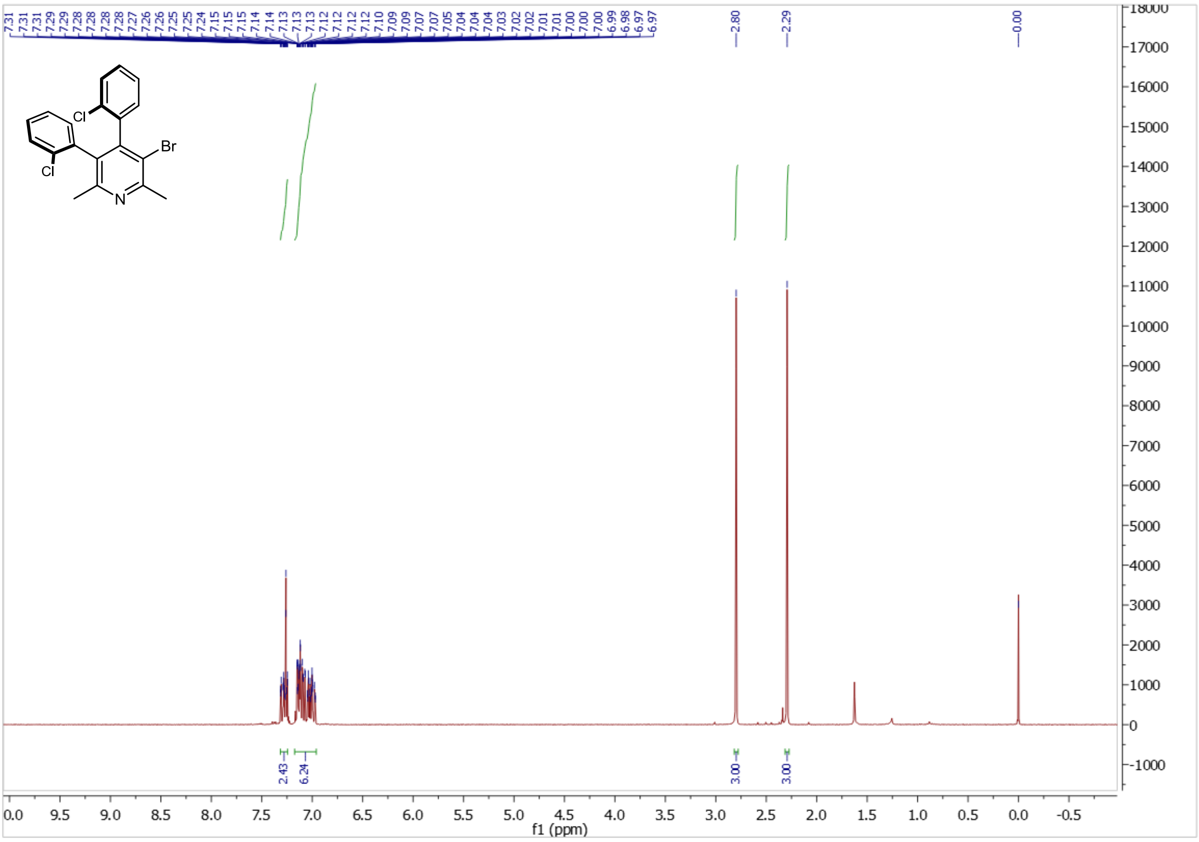

^{13}C NMR spectrum of **16** (75 MHz, $CDCl_3$)

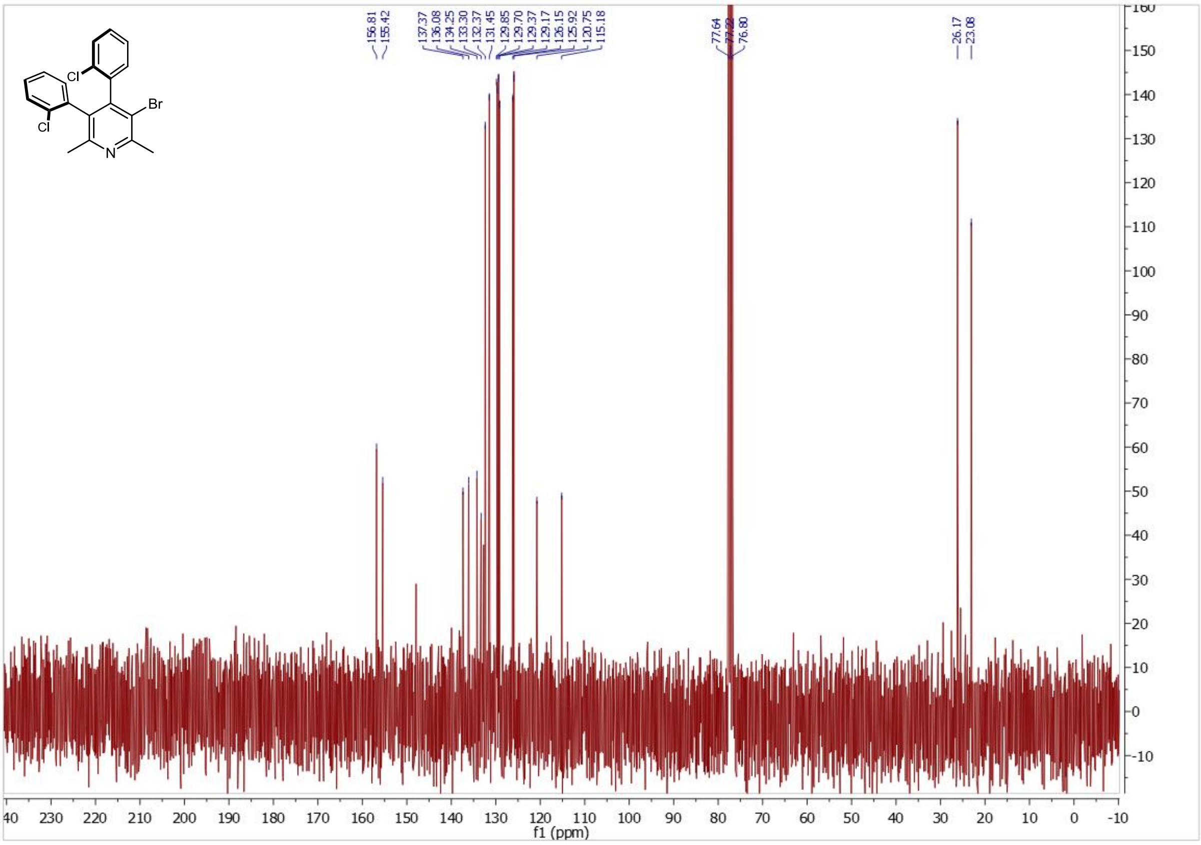

^{1}H NMR spectrum of **16** (300 MHz, DMSO) t=25°C

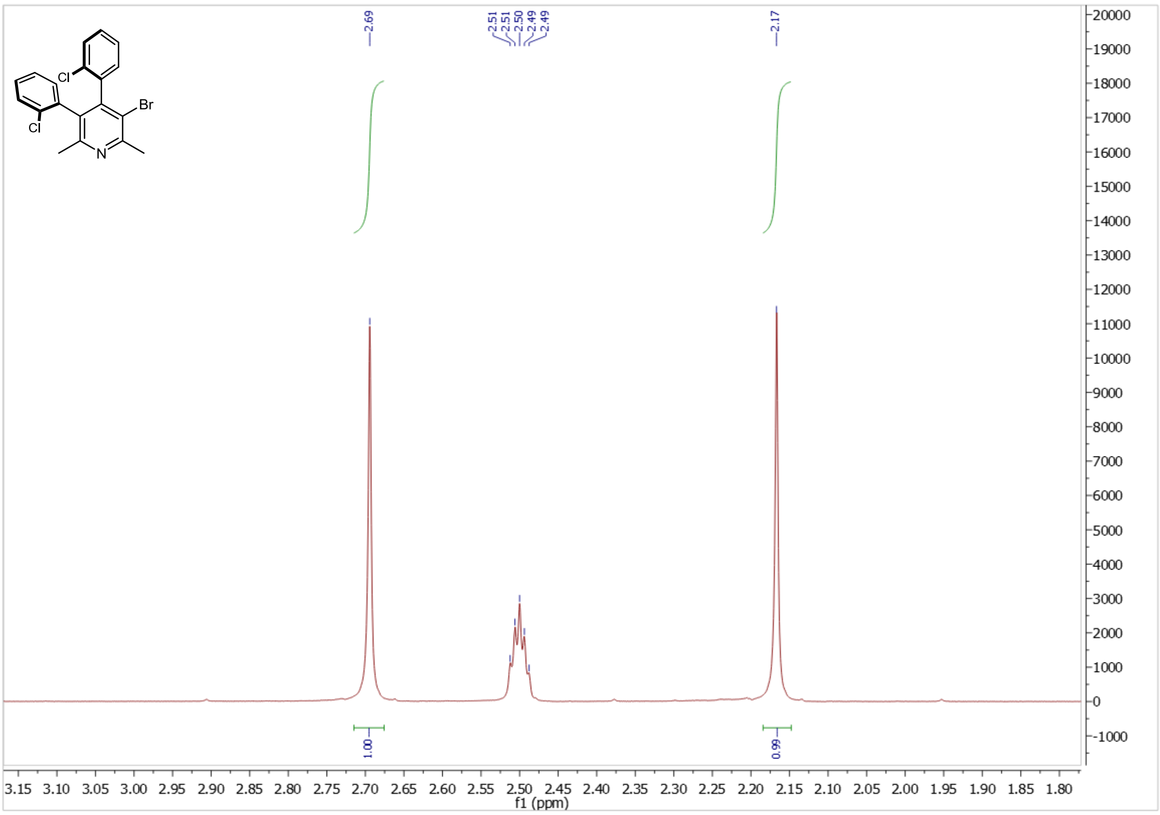

^{1}H NMR spectrum of **16** (300 MHz, DMSO) after 2 h heating at 120°C

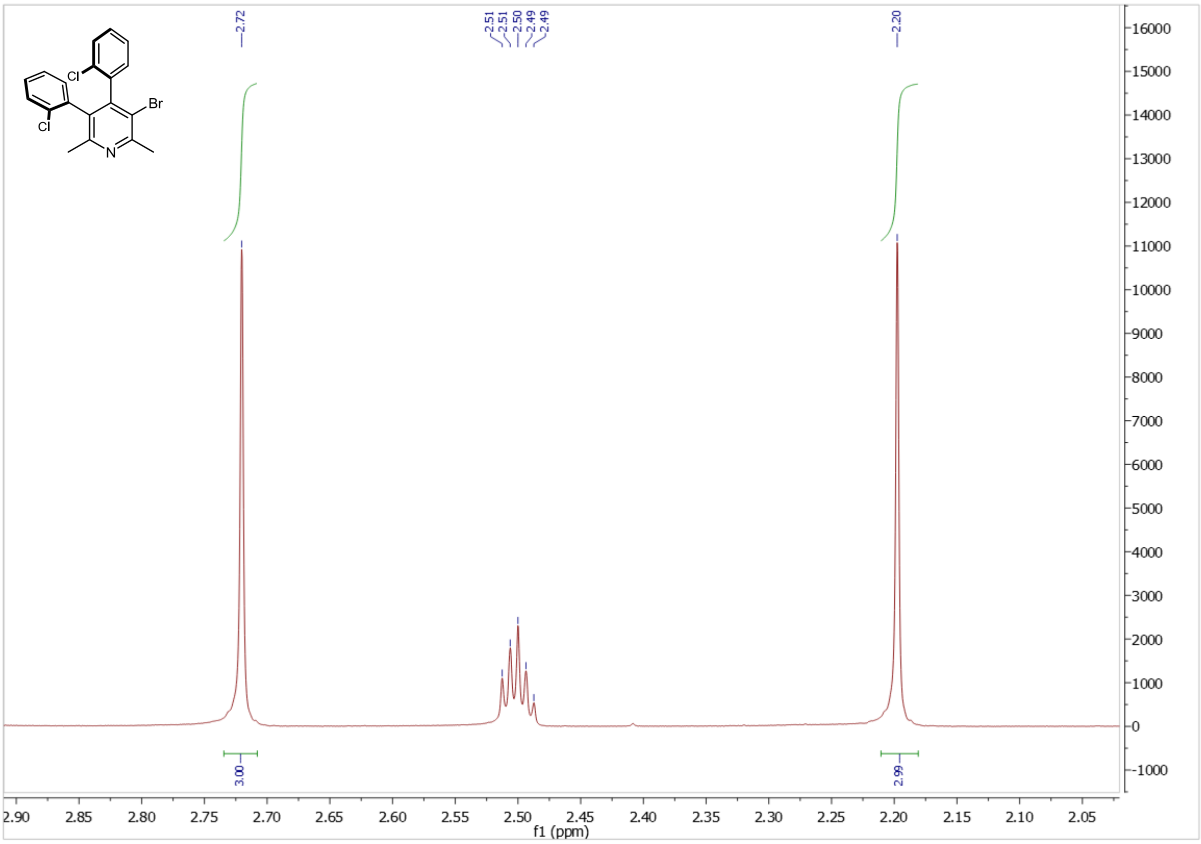

^{1}H NMR spectrum of **17** (300 MHz, $CDCl_3$)

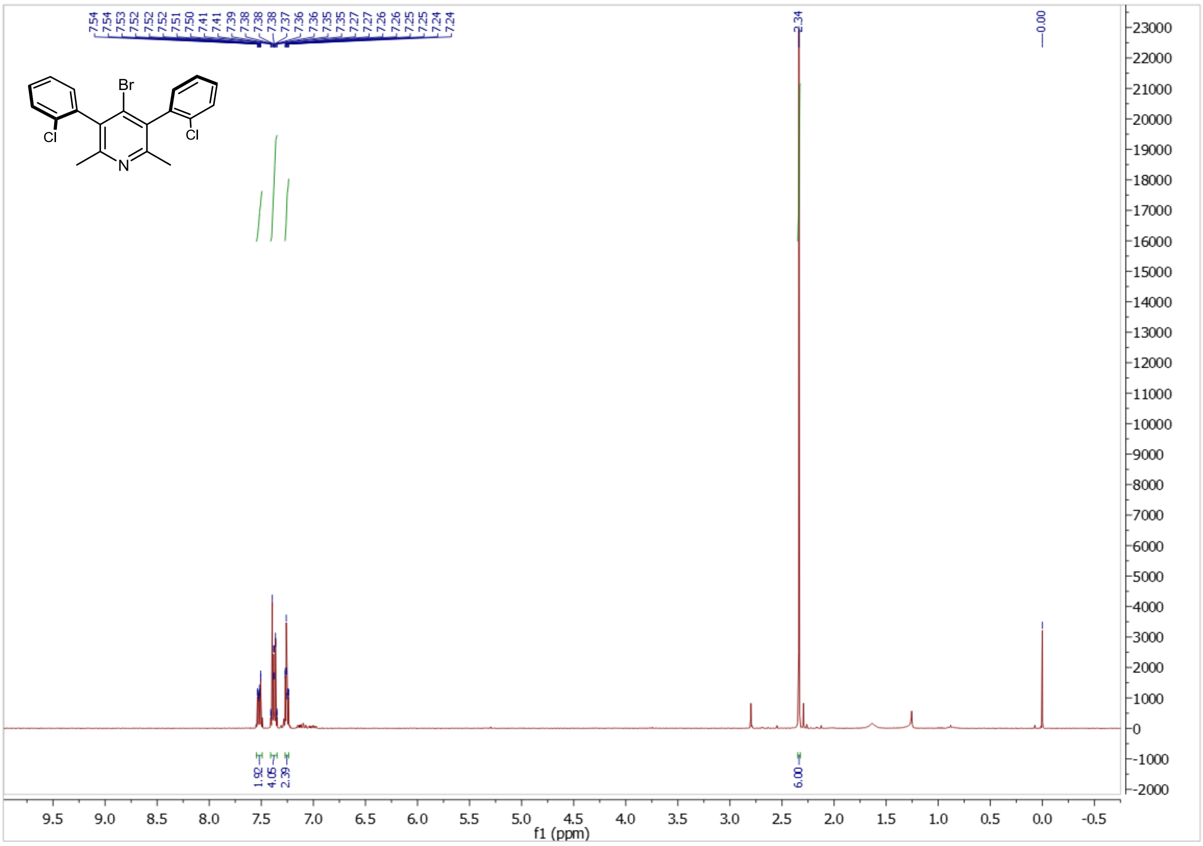

^{13}C NMR spectrum of **17** (75 MHz, $CDCl_3$)

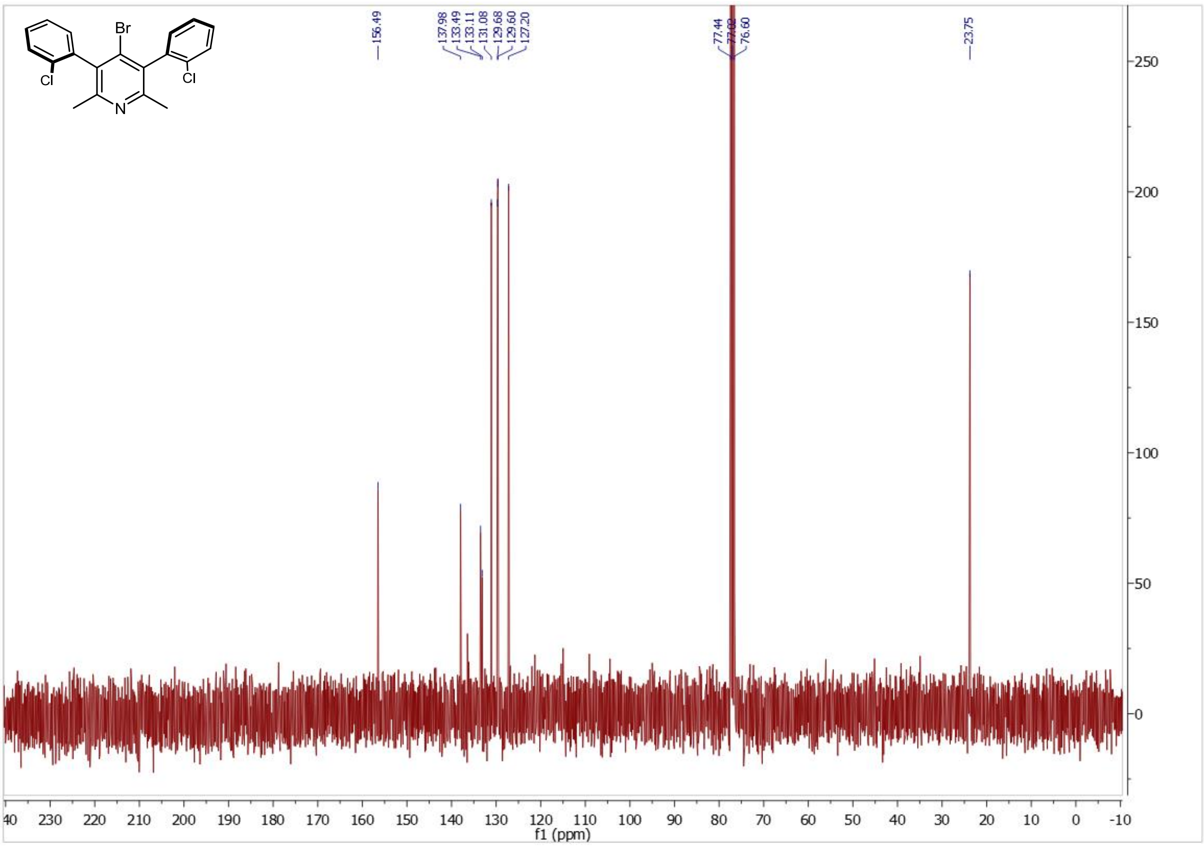

^{1}H NMR spectrum of **17** (300 MHz, DMSO) t=25°C

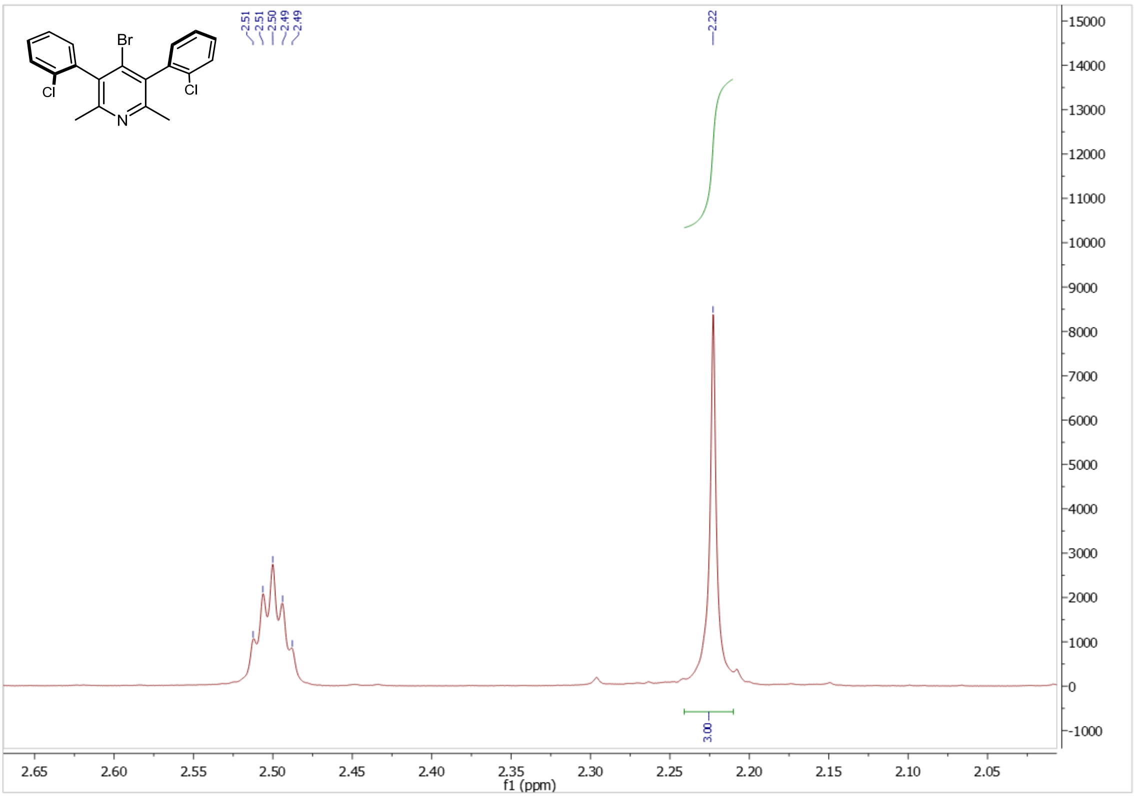

^{1}H NMR spectrum of **17** (300 MHz, DMSO) after 2 h heating at 120°C

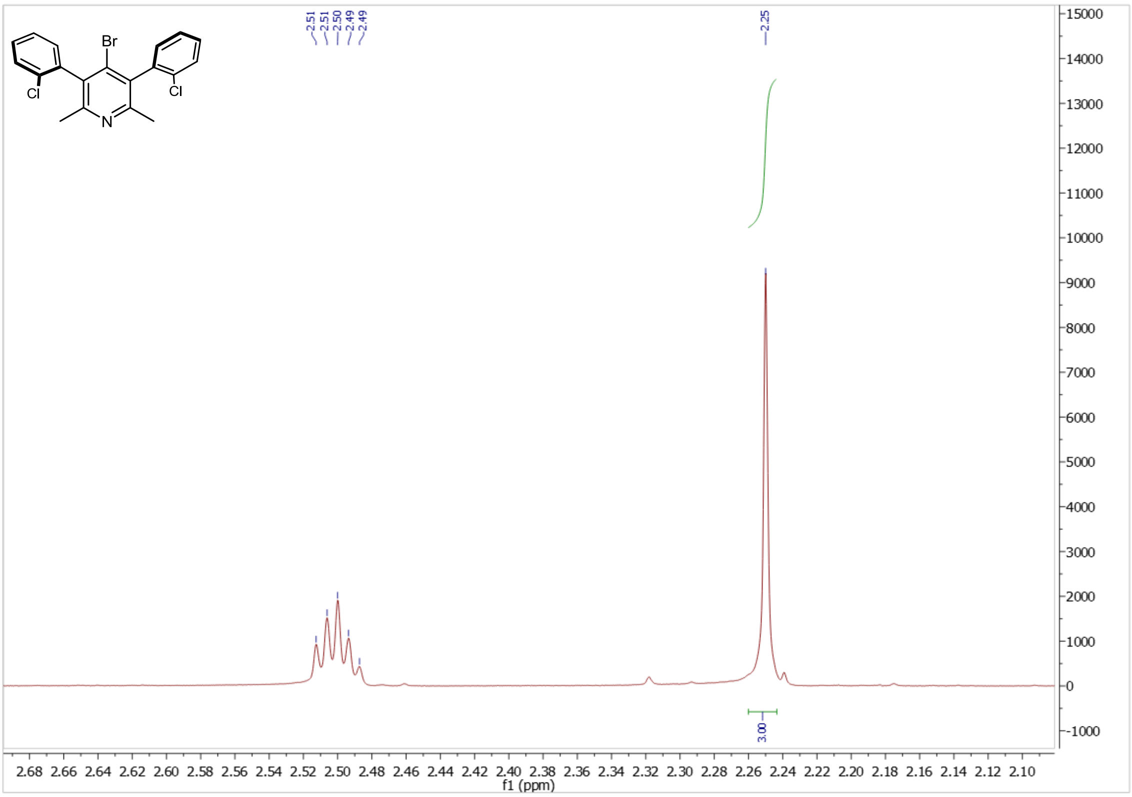

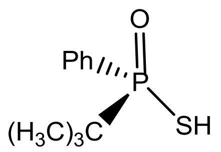

Structure of (+)-(*R*)-tert-butyl(phenyl) phosphinothioic acid

^{1}H NMR (CH_3 and OCH_3 protons) of racemic **2** in presence of (+)-(*R*)-tert-butyl(phenyl) phosphinothioic acid

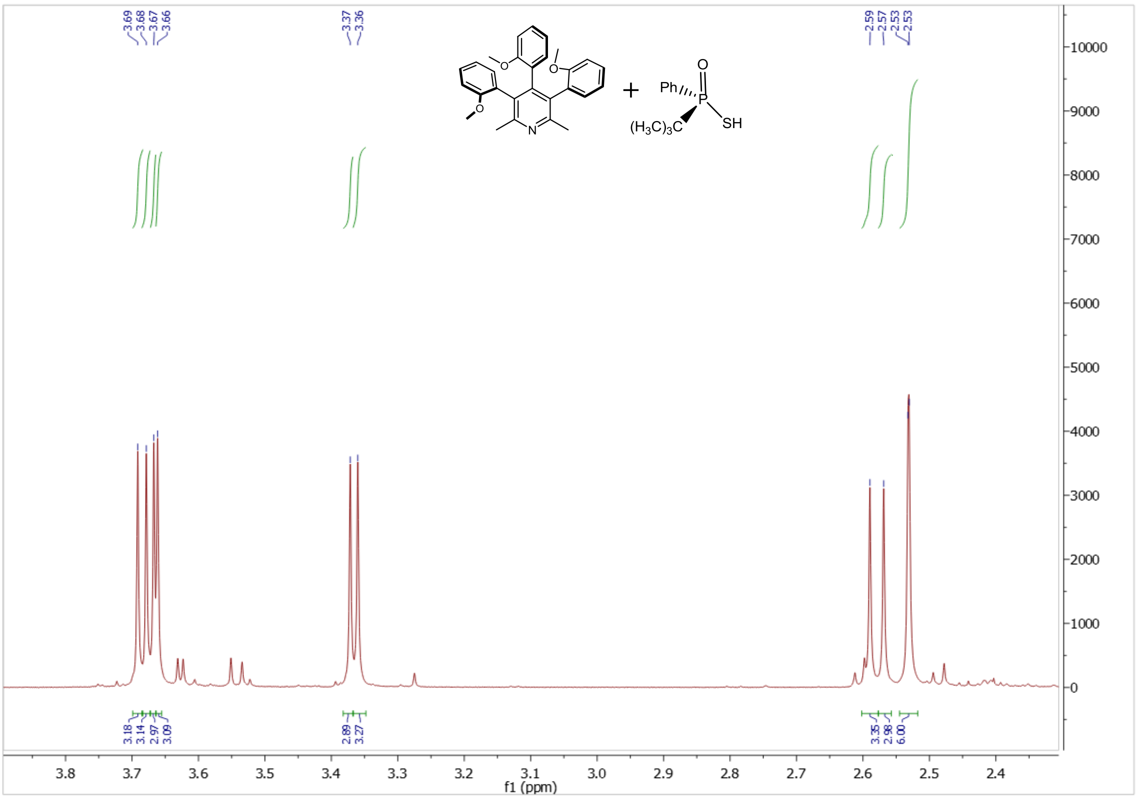

^{1}H NMR (CH_3 and OCH_3 protons) of racemic **6** in presence of (+)-(*R*)-tert-butyl(phenyl) phosphinothioic acid

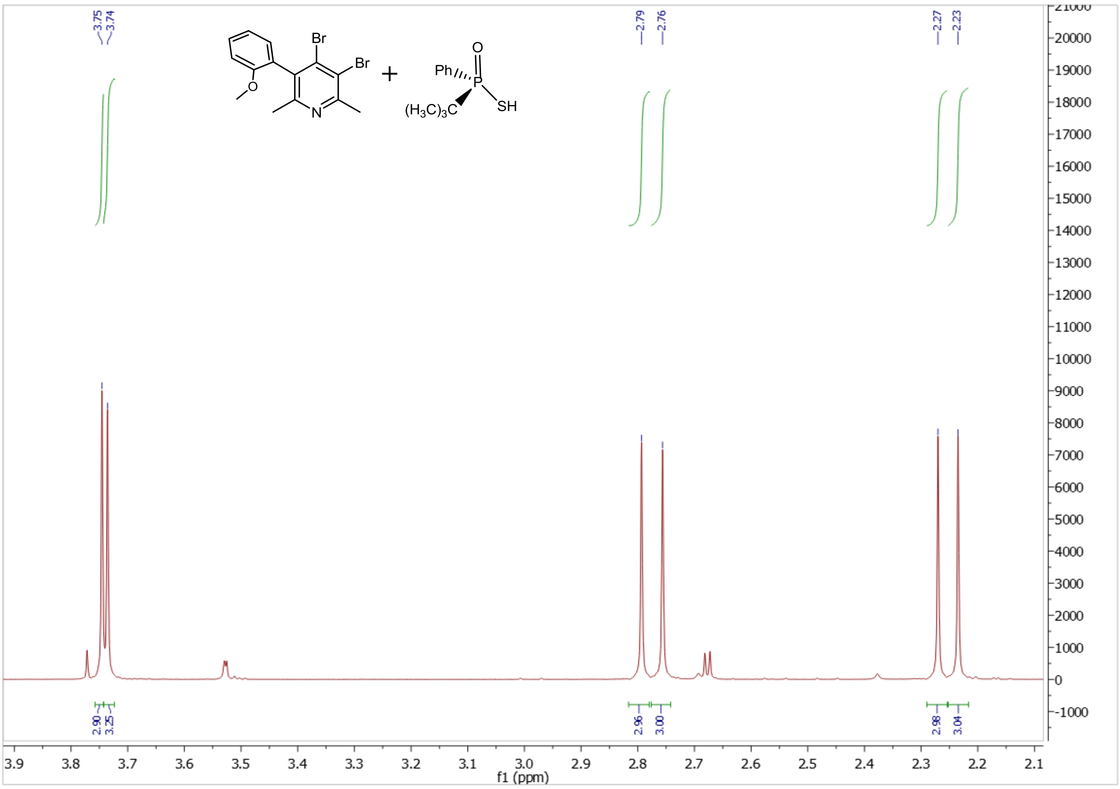

^{1}H NMR (CH_3 and OCH_3 protons) of racemic **7** in presence of (+)-(*R*)-tert-butyl(phenyl) phosphinothioic acid

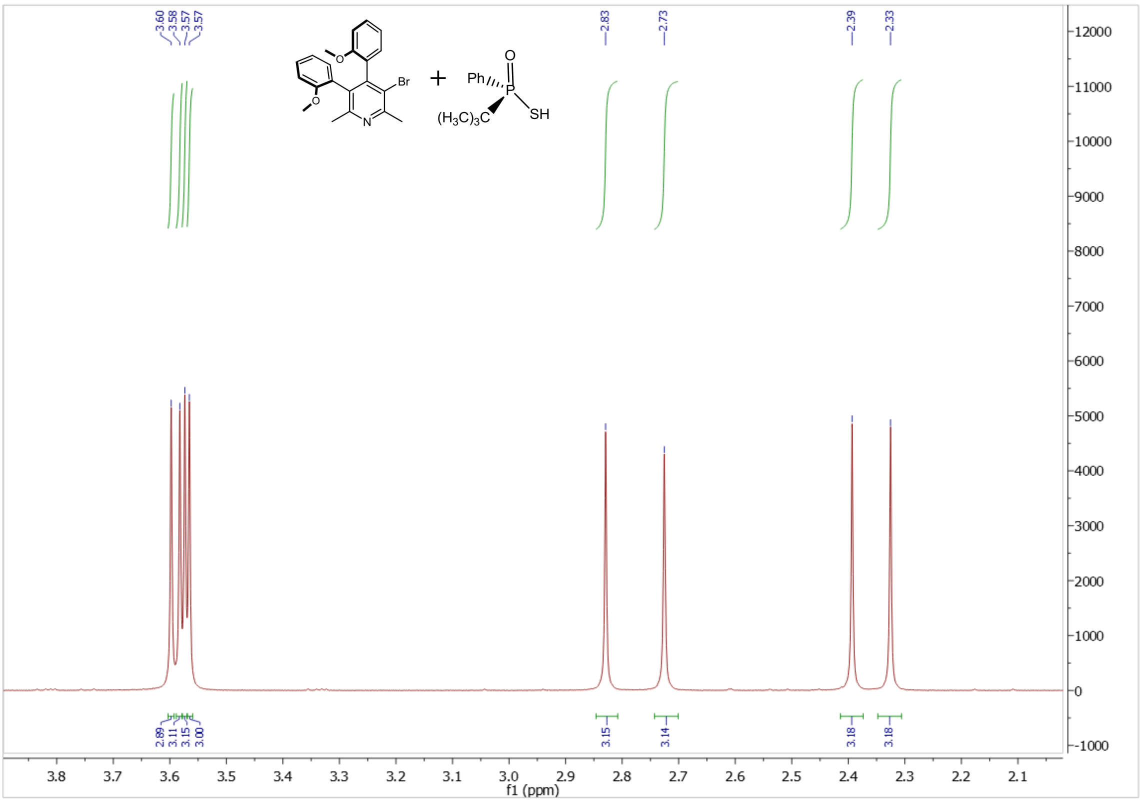

^{1}H NMR (CH_3 and OCH_3 protons) of racemic **8** in presence of (+)-(*R*)-tert-butyl(phenyl) phosphinothioic acid

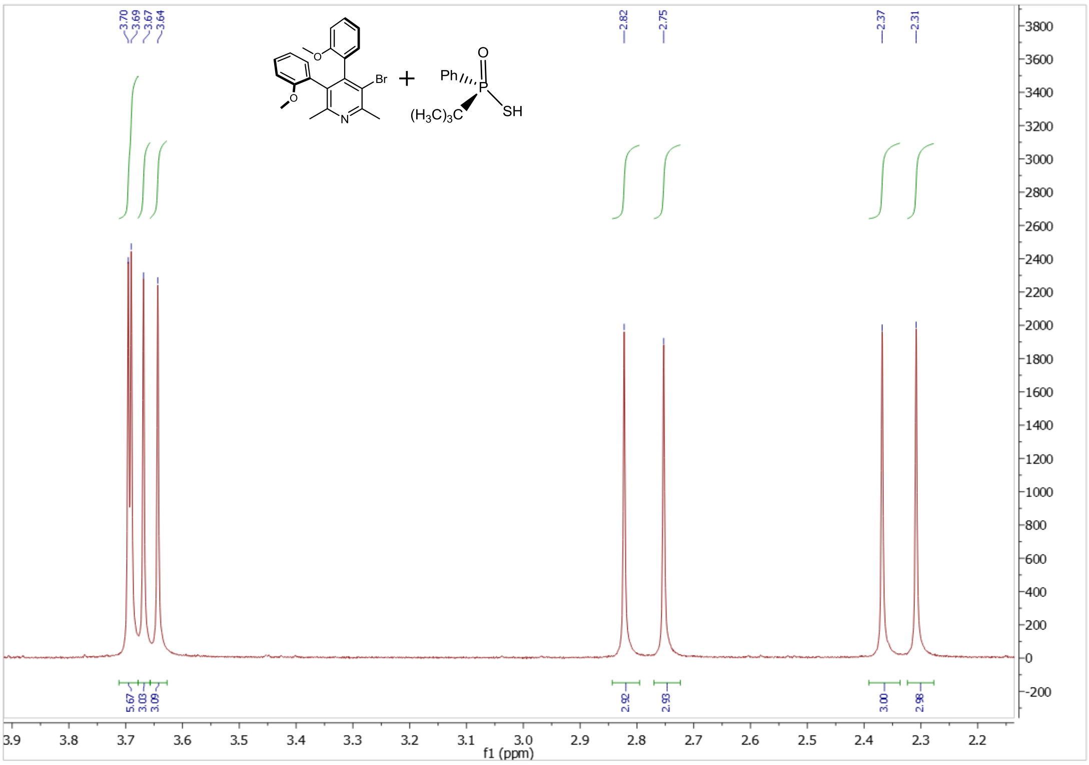

^{1}H NMR (CH_3 protons) of racemic **9** in presence of (+)-(*R*)-tert-butyl(phenyl) phosphinothioic acid

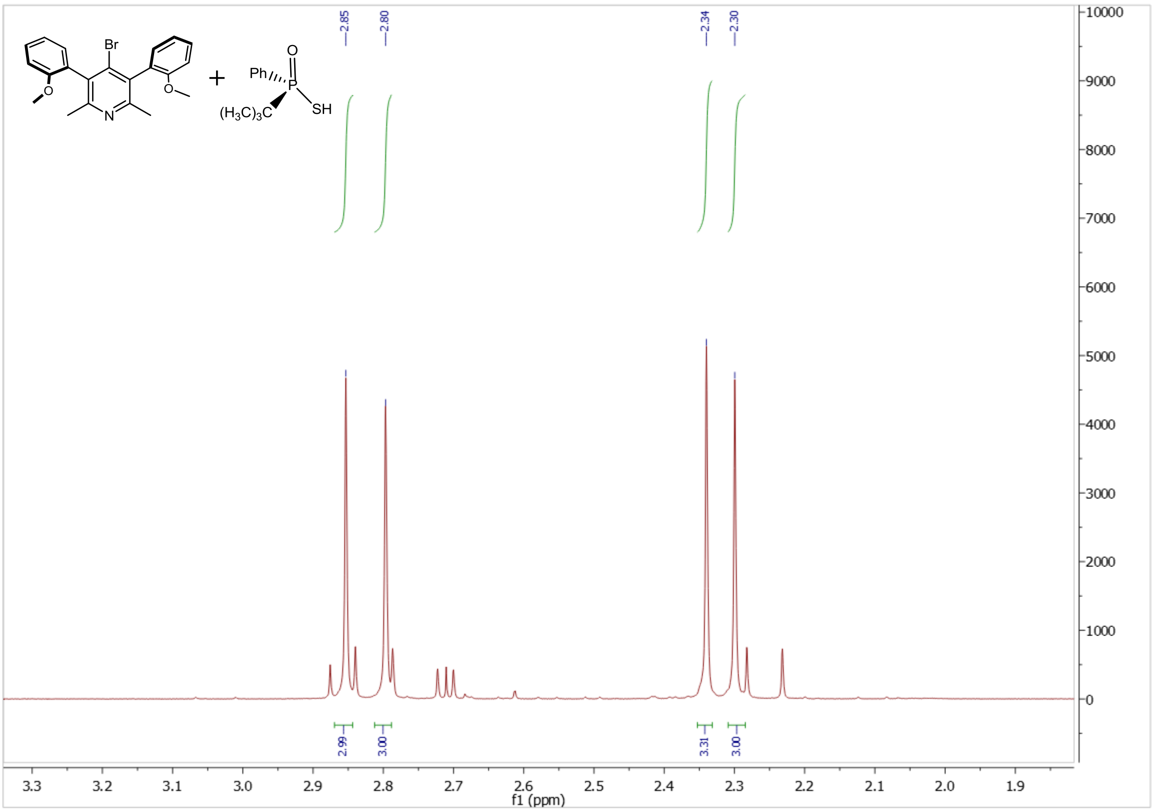

^{1}H NMR (CH_3 protons) of racemic **13** in presence of (+)-(*R*)-tert-butyl(phenyl) phosphinothioic acid

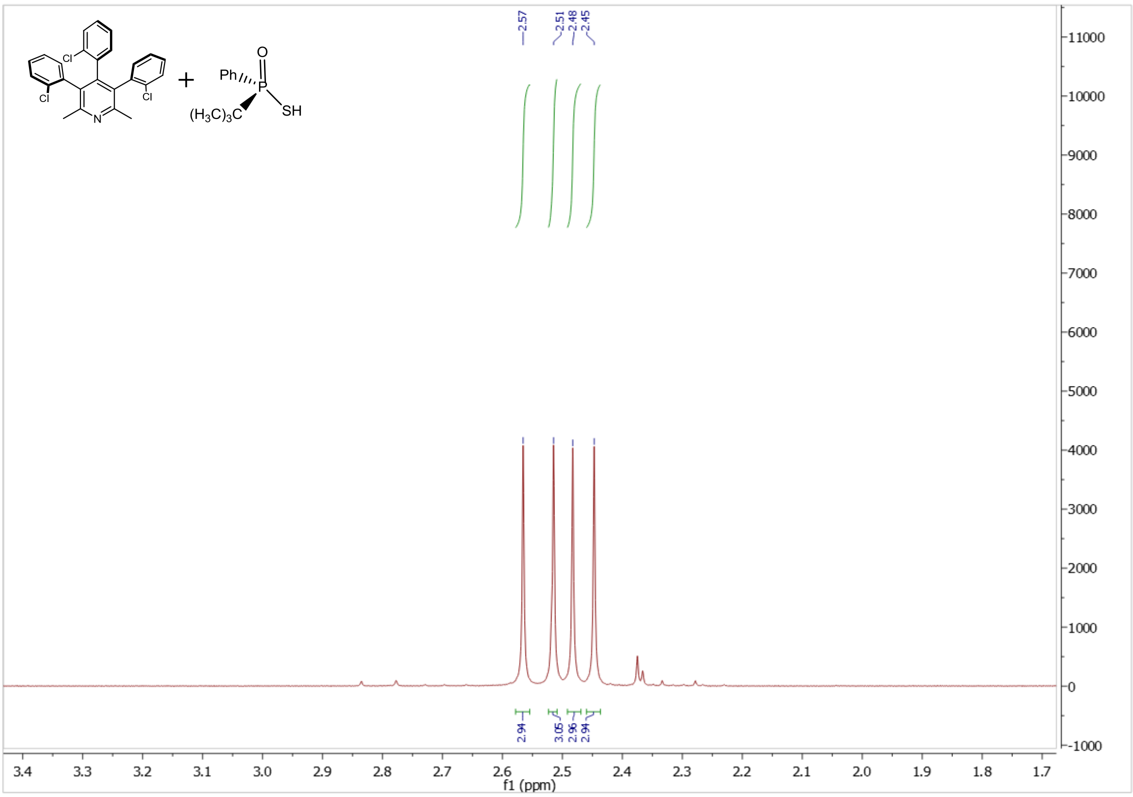

^{1}H NMR (CH_3 protons) of racemic **15** in presence of (+)-(*R*)-tert-butyl(phenyl) phosphinothioic acid

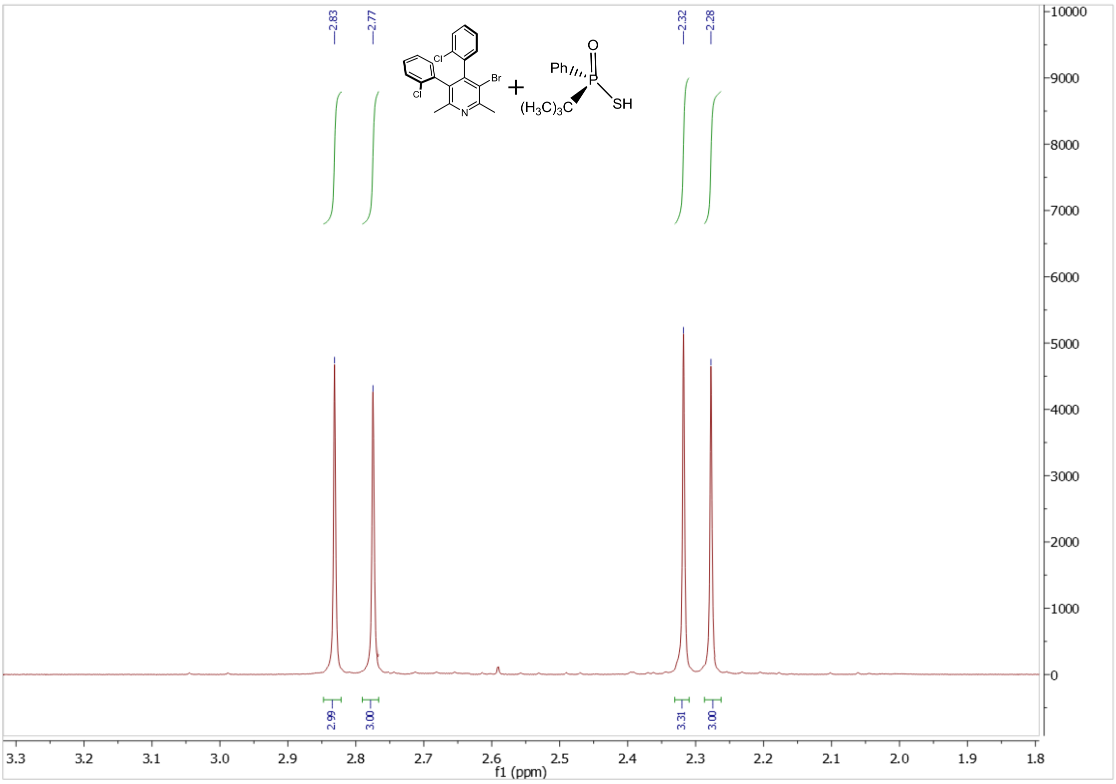

^{1}H NMR (CH_3 protons) of racemic **16** in presence of (+)-(*R*)-tert-butyl(phenyl) phosphinothioic acid

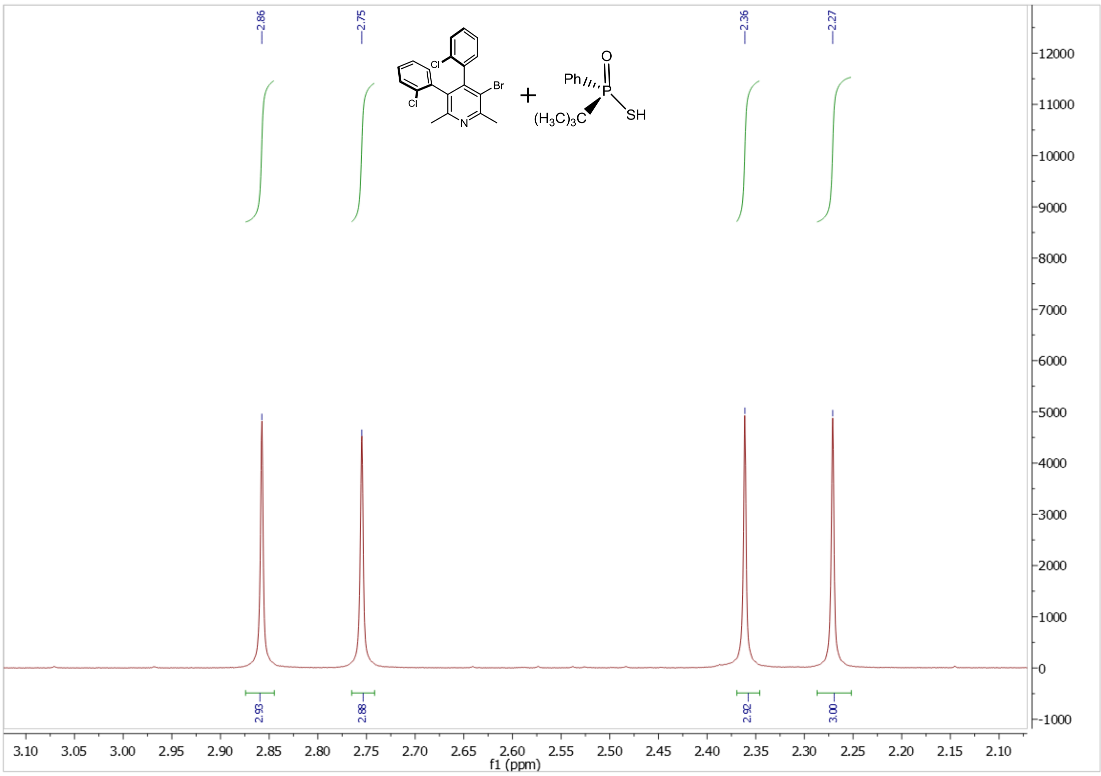

^{1}H NMR (CH_3 protons) of racemic **17** in presence of (+)-(*R*)-tert-butyl(phenyl) phosphinothioic acid

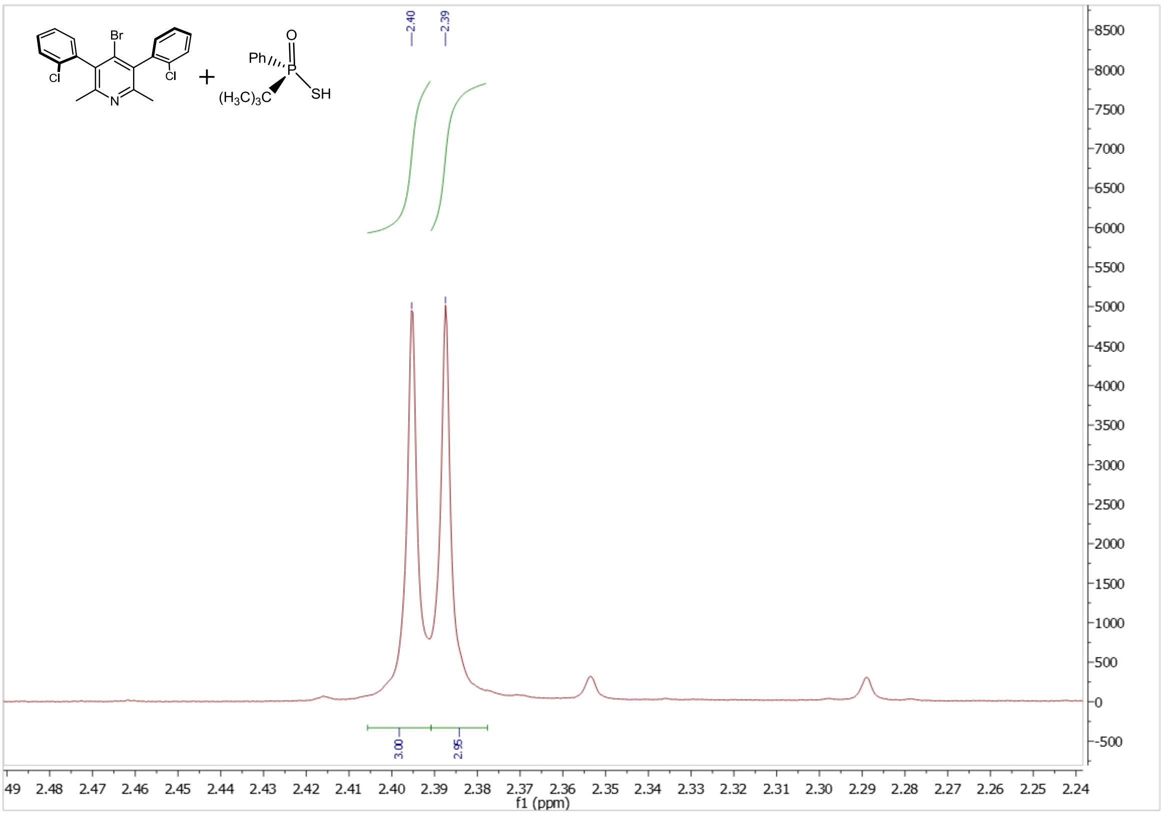

ORTEP diagram of compound **6**

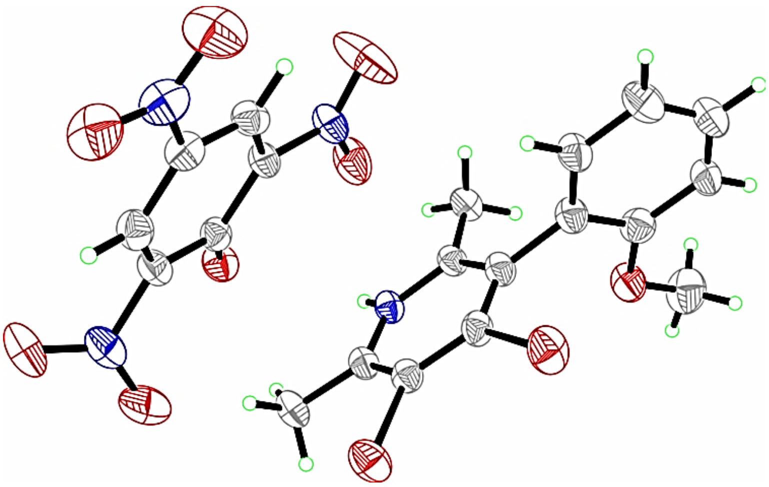

6 * picrate: yellow solid, mp 168.9-169.8°C, crystallization from CH_2Cl_2/hexane
CCDC-1528450 contains the supplementary crystallographic data for this paper. These data can be obtained free of charge from The Cambridge Crystallographic Data Centre via www.ccdc.cam.ac.uk/data_request/cif.

ORTEP diagram of compound **7**

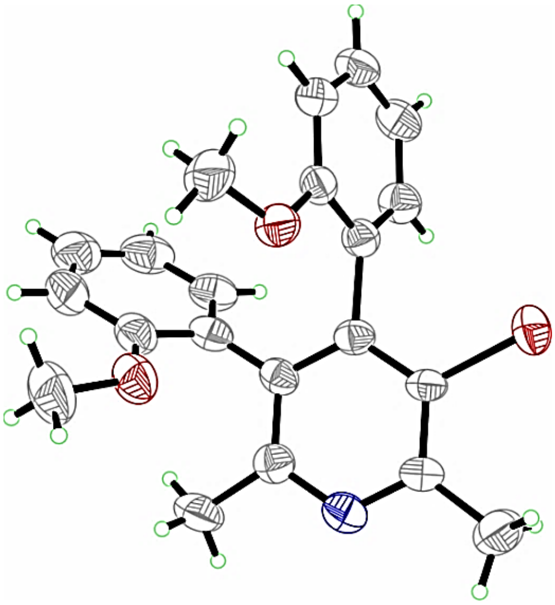

White solid, mp 158.5-159.5°C, crystallization from methanol.
CCDC-1528451 contains the supplementary crystallographic data for this paper. These data can be obtained free of charge from The Cambridge Crystallographic Data Centre via www.ccdc.cam.ac.uk/data_request/cif.

ORTEP diagram of compound **9**

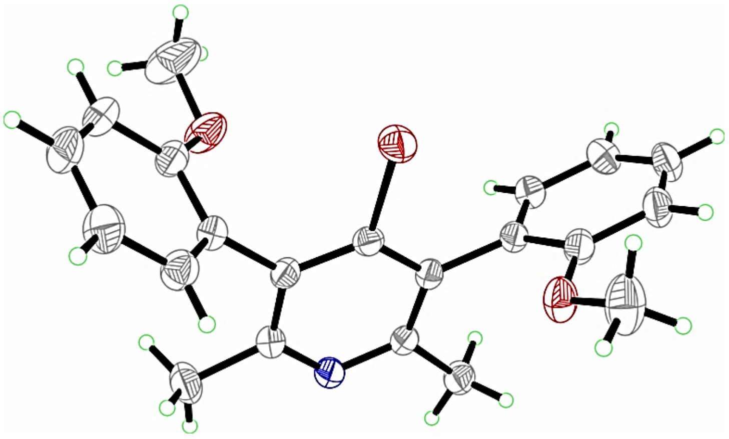

White solid, mp 186.5-187.5 °C, crystallization from methanol.
CCDC-1528454 contains the supplementary crystallographic data for this paper. These data can be obtained free of charge from The Cambridge Crystallographic Data Centre via www.ccdc.cam.ac.uk/data_request/cif.

ORTEP diagram of compound **10**

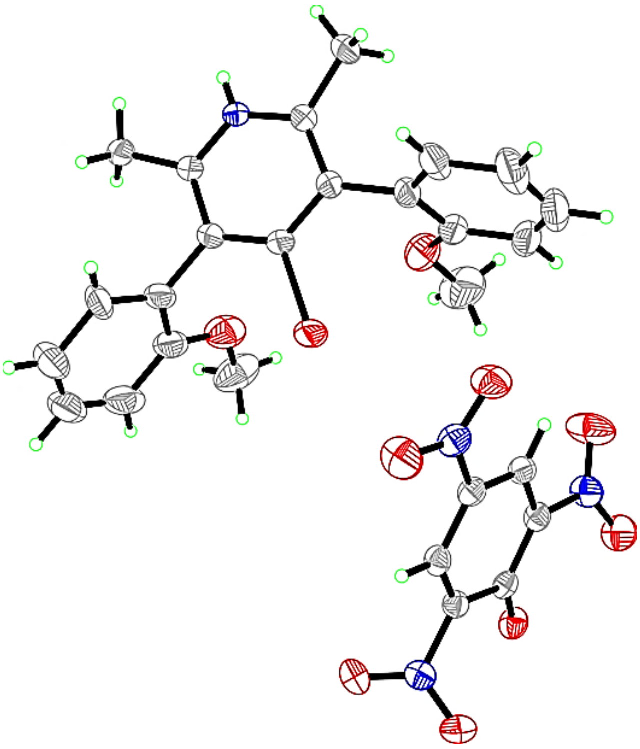

10 * picrate: yellow solid, mp 206.8-207.9°C, crystallization from dichloromethane/hexane.
CCDC-1528457 contains the supplementary crystallographic data for this paper. These data can be obtained free of charge from The Cambridge Crystallographic Data Centre via www.ccdc.cam.ac.uk/data_request/cif.

ORTEP diagram of compound **12**

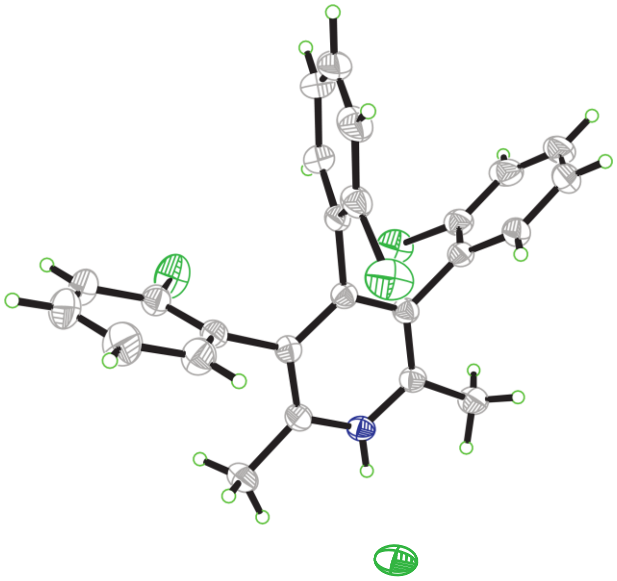

12 * HCl: white solid, mp 233.5-235°C, crystallization from methanol/ethyl acetate
CCDC-1528707 contains the supplementary crystallographic data for this paper. These data can be obtained free of charge from The Cambridge Crystallographic Data Centre via www.ccdc.cam.ac.uk/data_request/cif.

ORTEP diagram of compound **13**

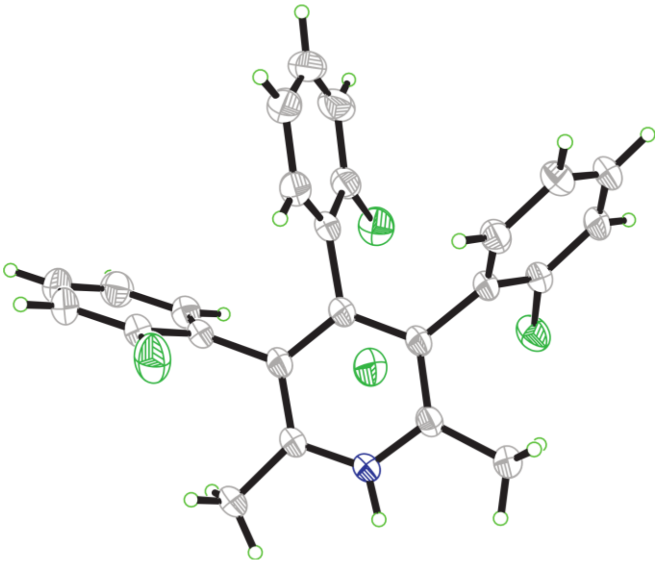

13 * HCl: white solid, mp 254-255.5°C, crystallization from dichloromethane/heptane
CCDC-1528679 contains the supplementary crystallographic data for this paper. These data can be obtained free of charge from The Cambridge Crystallographic Data Centre via www.ccdc.cam.ac.uk/data_request/cif.

ORTEP diagram of compound **14**

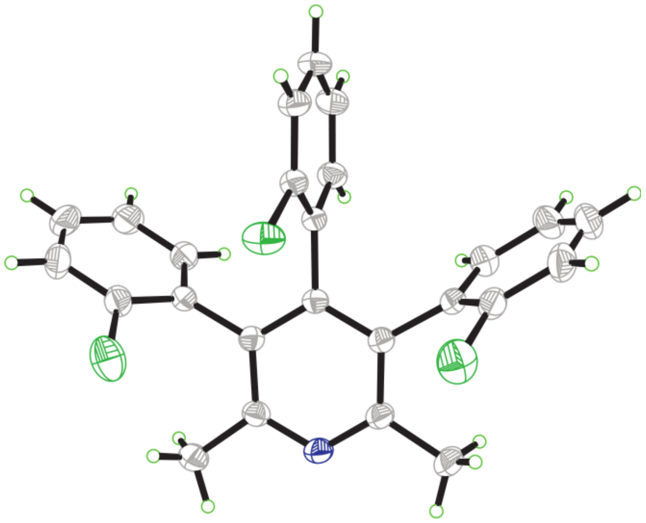

White solid, mp 107-109°C, crystallization from ethyl acetate.
CCDC-1528721 contains the supplementary crystallographic data for this paper. These data can be obtained free of charge from The Cambridge Crystallographic Data Centre via www.ccdc.cam.ac.uk/data_request/cif.

ORTEP diagram of compound **15**

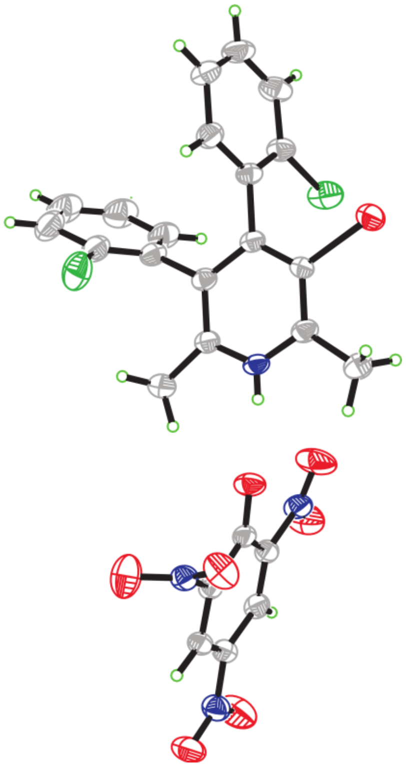

15 * picrate: yellow solid, mp 214-216°C, crystallization from dichloromethane/methanol

CCDC-1528655 contains the supplementary crystallographic data for this paper. These data can be obtained free of charge from The Cambridge Crystallographic Data Centre via www.ccdc.cam.ac.uk/data_request/cif.

ORTEP diagram of compound **16**

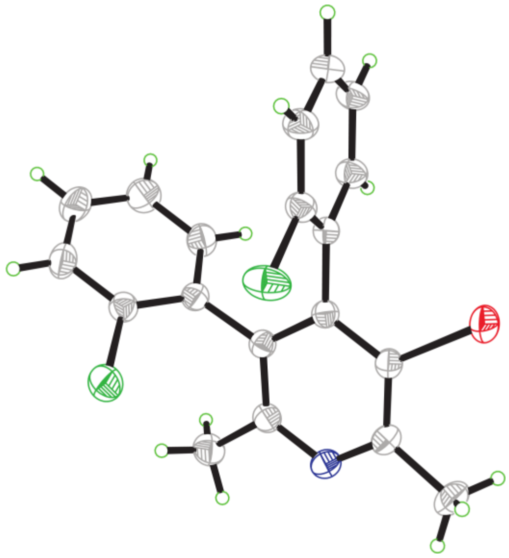

White solid, mp 153-155°C, crystallization from ethyl acetate.
CCDC-1528653 contains the supplementary crystallographic data for this paper. These data can be obtained free of charge from The Cambridge Crystallographic Data Centre via www.ccdc.cam.ac.uk/data_request/cif.

ORTEP diagram of compound **17**

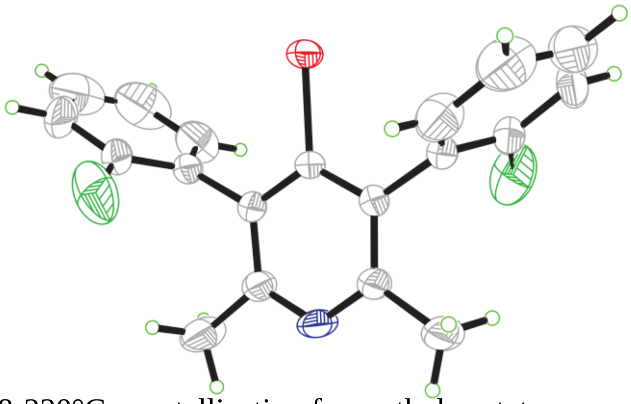

White solid, mp 228-230°C, crystallization from ethyl acetate.
CCDC-1528652 contains the supplementary crystallographic data for this paper. These data can be obtained free of charge from The Cambridge Crystallographic Data Centre via www.ccdc.cam.ac.uk/data_request/cif.
